# Leveraging opposition-based learning for solar photovoltaic model parameter estimation with exponential distribution optimization algorithm

**DOI:** 10.1038/s41598-023-50890-y

**Published:** 2024-01-04

**Authors:** Nandhini Kullampalayam Murugaiyan, Kumar Chandrasekaran, Premkumar Manoharan, Bizuwork Derebew

**Affiliations:** 1 Department of Electronics and Instrumentation Engineering, Bannari Amman Institute of Technology, Sathyamangalam, Erode, Tamil Nadu 638401 India; 2 Department of Electrical and Electronics Engineering, Karpagam College of Engineering, Coimbatore, Tamil Nadu 641032 India; 3 Department of Electrical and Electronics Engineering, Dayananda Sagar College of Engineering, Bangalore, Karnataka 560078 India; 4 Department of Statistics, College of Natural and Computational Science, Mizan-Tepi University, 260, Tepi Bushira, Ethiopia

**Keywords:** Engineering, Mathematics and computing

## Abstract

Given the multi-model and nonlinear characteristics of photovoltaic (PV) models, parameter extraction presents a challenging problem. This challenge is exacerbated by the propensity of conventional algorithms to get trapped in local optima due to the complex nature of the problem. Accurate parameter estimation, nonetheless, is crucial due to its significant impact on the PV system’s performance, influencing both current and energy production. While traditional methods have provided reasonable results for PV model variables, they often require extensive computational resources, which impacts precision and robustness and results in many fitness evaluations. To address this problem, this paper presents an improved algorithm for PV parameter extraction, leveraging the opposition-based exponential distribution optimizer (OBEDO). The OBEDO method, equipped with opposition-based learning, provides an enhanced exploration capability and efficient exploitation of the search space, helping to mitigate the risk of entrapment in local optima. The proposed OBEDO algorithm is rigorously verified against state-of-the-art algorithms across various PV models, including single-diode, double-diode, three-diode, and photovoltaic module models. Practical and statistical results reveal that the OBEDO performs better than other algorithms in estimating parameters, demonstrating superior convergence speed, reliability, and accuracy. Moreover, the performance of the proposed algorithm is assessed using several case studies, further reinforcing its effectiveness. Therefore, the OBEDO, with its advantages in terms of computational efficiency and robustness, emerges as a promising solution for photovoltaic model parameter identification, making a significant contribution to enhancing the performance of PV systems.

## Introduction

Addressing climate change and shaping effective energy policies have become urgent global priorities. In this context, the value of photovoltaic (PV) power generation cannot be overstated. It offers a pathway to harness electricity from solar radiation, all while bypassing greenhouse gas emissions^1,2^. The usage of PV systems has witnessed a substantial rise, despite their relative costliness, as part of the solution to these global issues. Recent research has concentrated on crafting precise models to maximize the productivity of renewable energy (RE) sources. These endeavours aim to provide viable alternatives to traditional fossil fuels, whose usage is tied to environmental pollution. Among various renewable sources, solar energy stands out. Its capacity to generate electricity through PV power is a green alternative that causes no environmental harm. PV systems, pivotal in renewable energy development, transform solar energy into electrical power^3–5^. However, they face challenges due to environmental influences like dust, weather changes, and temperature variations. These elements can decrease the efficiency of solar cells, making the need for accurate PV models crucial. Such models can help optimize the energy conversion process and mitigate these challenges. Solar energy is a significant renewable source, capable of generating electricity without excessive resource consumption or environmental pollution. Yet, the practical application of this source faces obstacles, including low photoelectric conversion and the need for precise PV cell modelling. Furthermore, the veracity of simulation results in various power systems relies heavily on correct PV module modelling. Therefore, developing robust mathematical models is essential for predicting solar cell parameters and understanding their behaviour^6–8^.

In PV equivalent circuits, the single-diode-model (SDM) is formed with one diode and two resistors^9^, while introducing two diodes results in the double-diode model (DDM)^10^, and so forth. As diodes increase, so do the uncertain variables within the photovoltaic models. For example, a single-diode PV model contains five unknown parameters, a DDM has seven, and a Triple-Diode Model (TDM) possesses nine^11,12^, and this progression continues. This illustrates that the complexity of the model grows with the increasing count of diodes. Parameters such as shunt-resistor, shunt-resistance current, series resistor, and saturation current, among others, are unaccounted for in these PV models. They must be computed and recovered from the PV characteristic curves. An accurate estimation of such parameters is vital for the optimal operation of solar cell models. Any misestimation can introduce significant discrepancies in the system output, leading to errors in the manufacturers' data. Therefore, determining these parameters is an essential task that significantly enhances the PV system's optimization and performance. The PV mathematical model equations are inherently implicit, nonlinear, multivariable, and multimodal. It is widely accepted among researchers that solving PV models presents substantial challenges. As such, multiple methods have been proposed to extract, evaluate, and simulate PV parameters in a precise, reliable, and timely manner^6,7,13,14^. The literature reveals that various PV models are typically utilized to examine the I–V characteristics of PV cells, with the SDM forming the fundamental basis. However, the SDM fails to account for recombination losses occurring in the depletion region. For this reason, besides SDM, this study considered the DDM and the TDM to model solar cells effectively. The DDM is acknowledged to provide more precise outcomes than the single-diode model because it factors in the effects of low current. Meanwhile, the TDM addresses the PV cell's intricate nonlinear behaviour and is perceived as a high precise model than the ideal, SDM, and DDMs. Furthermore, it depicts solar cell characteristics in situations involving leakage current. However, the TDM's mathematical resolution presents challenges due to its nonlinear equations. Therefore, the problems associated with the TDM are typically addressed by transforming them into optimization problems^15–17^.

Accuracy in parameter estimation for solar PV systems is crucial for several reasons: (i) Accurate parameter values are essential for optimizing the performance of PV systems. They ensure that the system operates at its maximum efficiency, thereby maximizing energy output; (ii) Precise parameters help in predicting the behaviour of PV systems under different conditions, which is vital for ensuring their reliability and longevity; (iii) Accurate estimation impacts the economic feasibility of PV installations. Overestimation or underestimation of system capabilities can lead to financial losses or underutilization of resources, and (iv) As solar technology evolves, the need for precise parameter estimation becomes even more critical to leverage advancements in PV materials and designs fully. Over the past several decades, researchers have made significant strides in understanding, optimizing, and estimating the parameters of various PV mathematical models. A range of methods has been proposed in the literature to handle and analyze the non-linearity of PV models, focusing on accuracy and efficiency. These solutions largely depend on the information sourced from the manufacturer's datasheet, which can be divided into two main categories: the I–V characteristic approach and the key point technique^18,19^. The key point-based technique extracts uncertain variables from the experimental samples offered by manufacturers. This approach simplifies the models by minimizing the expressions and incorporating a few experimental data. It identifies variables through significant points such as maximum power point (MPP) and data derived from the slopes of the experimental curves for the open-circuit voltage and short-circuit current points. However, this simplification of equations compromises the method's efficiency and accuracy. Recent research indicates that this method lags behind the I–V characteristic method in extracting parameters. The I–V characterization curve-based approach uses numerical optimization methods to determine the PV variables by minimizing the differences between experimental and calculated current data. Using this strategy, parameters can be identified in a variety of ways. Analytical, numerical, and technological approaches based on intelligent/metaheuristic methods have recently been classified as existent solutions in the scientific community for obtaining and determining PV characteristics from the I–V curves^20–22^.

In the analytical technique, a PV solar cell's precise mathematical model and its parameters are established using explicit equations. These equations use data from datasheets or significant points from the experimental I–V curve, such as open circuit voltage, short circuit current, and MPP current and voltage, to directly calculate the model parameters. Although straightforward to use, this approach is based on some simplifying presumptions that compromise the entire model's reliability and produce wildly exaggerated projections of economic returns^14,23^. Furthermore, these methods lack flexibility and are particularly sensitive to measurement noise. Another approach is numerical parameter identification methods, such as the widely-used Newton method^24^. These gradient-based algorithms have a straightforward procedure to accurately and quickly find the optimal model parameters. Nevertheless, they are highly sensitive to the initial parameter assumptions. If the technique starts from an initial solution distant from the optimal point, it may converge to a local optimal. Similar methods, like the Lambert W-based analytical method^25,26^, have been proposed for accurately determining the PV model parameters. This method is more effective in ease of implementation, robustness, efficiency, and accuracy than other methods. Yet, its application scope is limited and can easily fall into a local optimum point. Given that the PV cell model is generally nonlinear, the parameter estimation of the PV cell model exhibits multivariable and nonlinear characteristics. Consequently, numerical and analytical optimization methods may struggle to handle such nonlinear optimizations effectively. To address these weaknesses, intelligent algorithms have been introduced^27,28^. These aim to improve the parameter identification performance by minimizing the overall error between all experimental and simulated I–V curve data points.

Over the years, researchers have found that traditional methods, such as the I–V characteristic curve and key point approaches, may not be the most efficient means of deriving PV variables from equivalent PV models. In response, a shift towards adopting metaheuristic methods has been observed, as these methods demonstrate superior abilities in parameter extraction and analysis^6,29,30^. Unlike analytical and numerical methods subject to strict constraints and assumptions, metaheuristic methods offer a more flexible and accurate approach. The growing interest in meta-heuristic methods has led to the exploration of various algorithms, many of which are inspired by natural phenomena. These algorithms have consistently outperformed previous methods in terms of accuracy and efficiency. However, these methods are not without their challenges. High computation time is often required, and due to their stochastic nature, finding the optimal solution can still be elusive. Several techniques, such as genetic algorithm (GA)^31^, differential evolution (DE) algorithm^32,33^, cuckoo search (CS)^34,35^, artificial bee colony (ABC)^36^, teaching–learning-based optimization (TLBO)^37^, and particle swarm optimization (PSO)^38^, have been leveraged to minimize discrepancies between experimental and simulated current data in PV models. Despite their effectiveness, these techniques could benefit from enhancements in computational time efficiency. The GA, one of the most commonly used evolutionary algorithms, has been employed to solve many optimization problems, including extracting electrical parameters from various PV cells. Researchers have gone a step further by merging GA with other techniques, such as the Newton–Raphson or the interior point technique, to intensify the precision of PV variables^39,40^. Similarly, the DE and PSO algorithms, another popular evolutionary algorithm, have been modified and improved to cater to the PV model parameter extraction needs. However, like all stochastic algorithms, their accuracy and reliability can be unpredictable. Introducing hybrid algorithms has revolutionized extracting parameters from photovoltaic models in recent years. These innovative solutions include combinations of DE and reinforcement learning^41^, the gaining-sharing knowledge-based algorithm (GSK)^42^, the comprehensive learning Rao-1 algorithm^43^, hybrid PSO and grey wolf optimizer (HPSOGWO)^44^, the modified honey badger algorithm (HBA)^45^, and an adaptive harris hawk optimization (HHO) algorithm that employs sine–cosine transformations^46^. Each hybrid method is designed to strike a balance between exploration and exploitation in the extraction process. Moreover, an array of unique methods such as the chaotic tuna swarm algorithm (CTSA)^47^, gradient-based optimizer (GBO)^48–50^, slime mould algorithm (SMA)^51^, artificial hummingbird optimizer^52^, butterfly optimization algorithm (BOA)^53^, improved arithmetic optimization algorithm (IAOA)^15^, mountain gazelle optimizer (MGO)^54^, resistance–capacitance optimizer^55^, and a war strategy optimization (WSO) algorithm^56^ inspired by ancient warfare tactics have also been proposed for the extraction of unknown parameters of solar PV systems. Table 1 additionally encompasses pertinent information, including the specific performance criteria, enhanced method, electrical model, solar cell/panel type, and the data employed to estimate parameters. This table offers a thorough evaluation of numerous studies.

**Table 1:** Literature study.

References	PV models	Solar cell/module	Improved algorithm	Performance criteria	Results
^57^	SDM, DDM PV Module	R.T.C. France, Photowatt-PWP201, STM6–40/36, SM55, KC200GT, ST40	Classified perturbation mutation-based PSO (CPMPSO)	Root-mean-square error (RMSE)	The effectiveness of CPMPSO is estimated using PV module models
^47^	TDM	KC200GT PV module, SM55, RTC France Si	Chaotic tuna swarm optimizer (CTSO)	RMSE, integral absolute error (IAE), R^2^	The utilization of the CTSO algorithm, which incorporates a chaotic tent map and the Newton–Raphson method, yields enhanced exploitation and exploration phases, ultimately leading to the attainment of a global solution within a reduced timeframe
^58^	SDM, DDM, TDM	R.T.C France, Photowatt-PWP201	Rao algorithm	RMSE	The Rao method enhances the overall search capabilities of the original Rao algorithm by the use of a novel search strategy that relies on three distinct search equations
^59^	DDM	Calyxo CX3, JAP6–60–250 W/3BB, JAM6–60–260	Wind-driven-based fruit fly optimization (WDFO)	Normalized RMSE, mean absolute percentage error (MAPE), R2	Merging the features showed improved viability, convergence speed, and accuracy compared to the original algorithms
^60^	SDM, DDM, TDM, PV Module	Photowatt-PWP201, STM6–40/36, STP6–120/36	Population diversity-controlled DE (PDcDE)	RMSE	In the PDcDE algorithm, the basic DE algorithm's optimization process was enhanced using the diversity feature, showing superior efficiency and effectiveness for PV systems' parameter estimation
^61^	SDM, DDM, TDM	Photowatt-PWP201, STM6–40/36, STP6–120/36, KC200GT, SM55, ST40w	Memory-based gorilla troops optimizer (MGTO)	RMSE	The MGTO algorithm, which combines the gorilla memory-saving and explorative gorilla with adaptive mutation mechanism techniques, improves effectiveness by evading the local optima
^33^	SDM, DDM, PV module	Photowatt-PWP 201, STM6–40/36, RTC France cell	DE	RMSE	The DE algorithm, which estimated parameters using I–V samples structured using the Lambert-W function, showed greater precision and convergence speed compared to PSO, PSO alternatives and GA
^62^	SDM, DDM, TDM, PV Module	SM55, KC200GT, ST40, R.T.C. France	Laplacian Nelder-Mead hunger games search (LNMHGS)	RMSE, IAE, RE	The LNMHGS algorithm effectively achieved a balance between exploration and exploitation in the search process of the HGS method by incorporating the Laplacian approach and Nelder-Mead simplex approaches
^63^	SDM, DDM, TDM	STP6–120/36, STM6–40/36, Photowatt-PWP201 PV Modules, R.T.C. France	Heterogeneous differential evolution (HDE)	RMSE	The HDE method exhibited superior accuracy and stability in parameter estimation compared to the DE algorithm
^64^	SDM, DDM, TDM, PV Module	SM55, SW255, KC200GT, R.T.C. France	Robust niching chimp optimization (RN-ChOA)	RMSE	The RN-ChOA algorithm, which draws inspiration from the niche notion, has shown superior performance in predicting the parameters of the PV model compared to other alternative algorithms
^46^	SDM, DDM, TDM, PV Module	SM55, KC200GT, ST40, Photowatt-PWP201 PV modules, R.T.C. France	Adaptive harris hawk optimization (ADHHO)	Absolute error (AE), RMSE, normalized RMSE	The ADHHO algorithm has demonstrated effectiveness and efficiency in extracting unidentified variables within the PV model
^65^	DDM	TITAN-12–50 PV	Improve AOA	MAE, normalized root-mean-square deviation (NRMSD)	The IAOA enhanced the original algorithm's local search ability by using a new search operator, demonstrating superiority in terms of RMSD, MAE, and RMSE, MAE
^66^	SDM, DDM, TDM, PV Module	Photowatt-PWP201, KC200GT, ST40 PV modules, R.T.C. France	Opposition-based learning gradient-based optimization (OBGBO)	RMSE	The OBGBO showed significantly increased discovery and exploitation capacity compared to other algorithms in parameter definition
^67^	SDM, DDM, TDM	RTC France PV cell	Improved EO	RMSE	The use of the balance optimizer algorithm, in conjunction with the backpropagation and IEO methods, demonstrated superior performance compared to EO, PSO, GWO and ABC. This was achieved by efficiently leveraging the global and local search capabilities inherent in the algorithm
^68^	SDM, DDM, PV Module	R.T.C. France, SM55, Photowatt-PWP201, KC200GT, ST40	Performance-guided JAYA algorithm (PGJAYA)	RMSE	PGJAYA is suggested to find the PV cell/module model parameters
^69^	SDM, DDM, PV module	Photowatt-PWP201, R.T.C France	Hybrid seagull optimization algorithm (HSOA)	IAE, RMSE	The HSOA algorithm, which improved the basic SOA's optimization capability with three modified strategies, has been developed

Furthermore, various hybrid approaches have been suggested to address the limitations of single algorithms and enhance the efficiency of parameter estimation for photovoltaic models. These include the ABC algorithm with DE^70^, teaching–learning-based ABC (TLBABC)^71^, collaborative intelligence of different swarms^72^, Opposition-Based Flower Pollination Algorithm and Nelder-Mead simplex (OBFPA-NM)^73^, hybrid GWO and PSO^44,74^, Levenberg–Marquardt algorithm combined with simulated annealing algorithm (LMSAA)^75^, and hybrid firefly and pattern search algorithm (HFFPSA)^76^, hybrid GWO and CS algorithm^77^, etc. These approaches combine different techniques to achieve more effective results. The RMSE between the experimental and calculated current for the PV model is lower in OBFPA-NM compared to TLBABC and LMSAA. While the OBFPA-NM and HFFPSA methods yield the same RMSE value for both the SDM and DDM models, the estimated parameters obtained from these two approaches are distinct. When comparing the DDM, OBFPA-NM demonstrates superior performance over TLBABC. It was noted that the RMSE of OBFPA-NM was marginally lower than that of HFFPSA while predicting parameters for an SDM of the PV module. The effectiveness of these strategies varies depending on the task at hand, and their ability to produce accurate results quickly is heavily influenced by selecting the appropriate algorithm parameters. These approaches have recently garnered increased interest due to their lack of stringent requirements.

Despite the plethora of meta-heuristic algorithms available to researchers seeking to extract PV model parameters, achieving accurate and reliable results remains a complex and challenging task. This persistent challenge underscores the need for continual refinement and innovation in developing algorithms and methods for PV parameter extraction. This brings us to an essential conundrum: Can we effectively address this issue using current algorithms without sacrificing accuracy and stability? The “no-free-lunch (NFL)” theorem provides a fitting response to this query^78^. This theorem posits that no single algorithm can optimally resolve all optimization issues, as the superior performance of an algorithm in solving one specific problem does not guarantee an equivalent level of success in tackling other issues. Consequently, the quest for an ideally suitable meta-heuristic algorithm remains an ongoing research topic. The NFL theorem has laid the groundwork for numerous studies and allowed for the customization of existing algorithms to cater to novel problem classifications.

Historically, precise extraction of PV model parameters has been a complex task. This has motivated our development of the opposition-based exponential distribution optimizer (OBEDO). The original exponential distribution optimizer (EDO)—an algorithm known for its simplicity, efficiency, and fast convergence—demonstrated its strength in addressing global optimization issues^79^. However, despite its success in global optimization problems, the EDO faced limitations when dealing with local optima. To overcome these limitations, this study introduced OBEDO, an advanced EDO version incorporating an opposition-based learning approach. These strategies ensure a more accurate and precise extraction of parameters from various PV models. One pivotal strategy, opposition-based learning (OBL), is a memory repository that records prior positions^80^. These records are then compared with newly generated positions to inform positioning adjustments. The result is a system better equipped to navigate local optima and identify promising new positions. The main contributions of this paper can be summarized as follows:

- The introduction of OBEDO, engineered to extract PV model parameters effectively.
- Incorporating the OBL technique is designed to enhance the quality of positions by utilizing a history of prior positions.
- The comparison of OBEDO with other recognized algorithms using various PV models.
- Through comprehensive experimental results and statistical analyses, this study demonstrates the superior performance of OBEDO.

The rest of this document is organized as follows: Section “Photovoltaic modelling and problem formulation” delves into the various photovoltaic models, meticulously explaining their mathematical aspects, and explores the crafting of the objective function. Section “Proposed opposition-based exponential distribution optimizer” provides a succinct overview of the exponential distribution optimizer, thoroughly elaborating on the structure of the suggested OBEDO methodology. Section “Results and discussions” examines the results of various case studies, providing a profound analysis of the experiments. The final section wraps up the study, offering future research directions.

## Photovoltaic modelling and problem formulation

This section details the various photovoltaic modes and their respective mathematical modelling. Furthermore, the objective function construction is also deliberated.

### Photovoltaic modelling

Photovoltaic devices translate sunlight straight into electricity utilizing the photovoltaic effect. Accurately depicting these mechanisms is crucial to predicting their performance, designing systems, and undertaking thorough analysis. The three main models applied in PV system representation are SDM, DDM, and TDM^16,81–83^. Typically, a cell is represented through a singular current source, denoted as ${I}_{ph}$. The photocurrent, ${I}_{ph}$, is reliant on the intensity of solar radiation.

**Figure 1:**
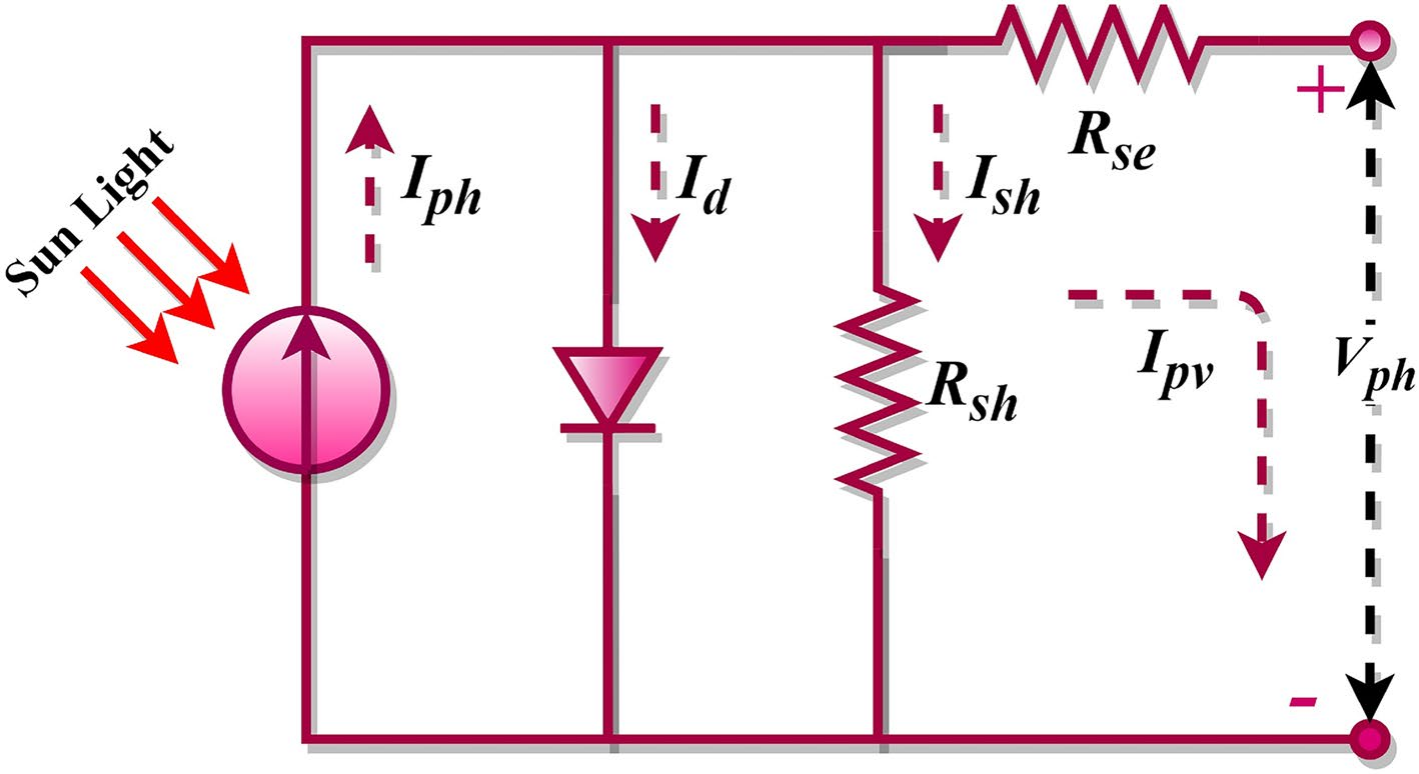
SDM of the photovoltaic cell.

**Figure 2:**
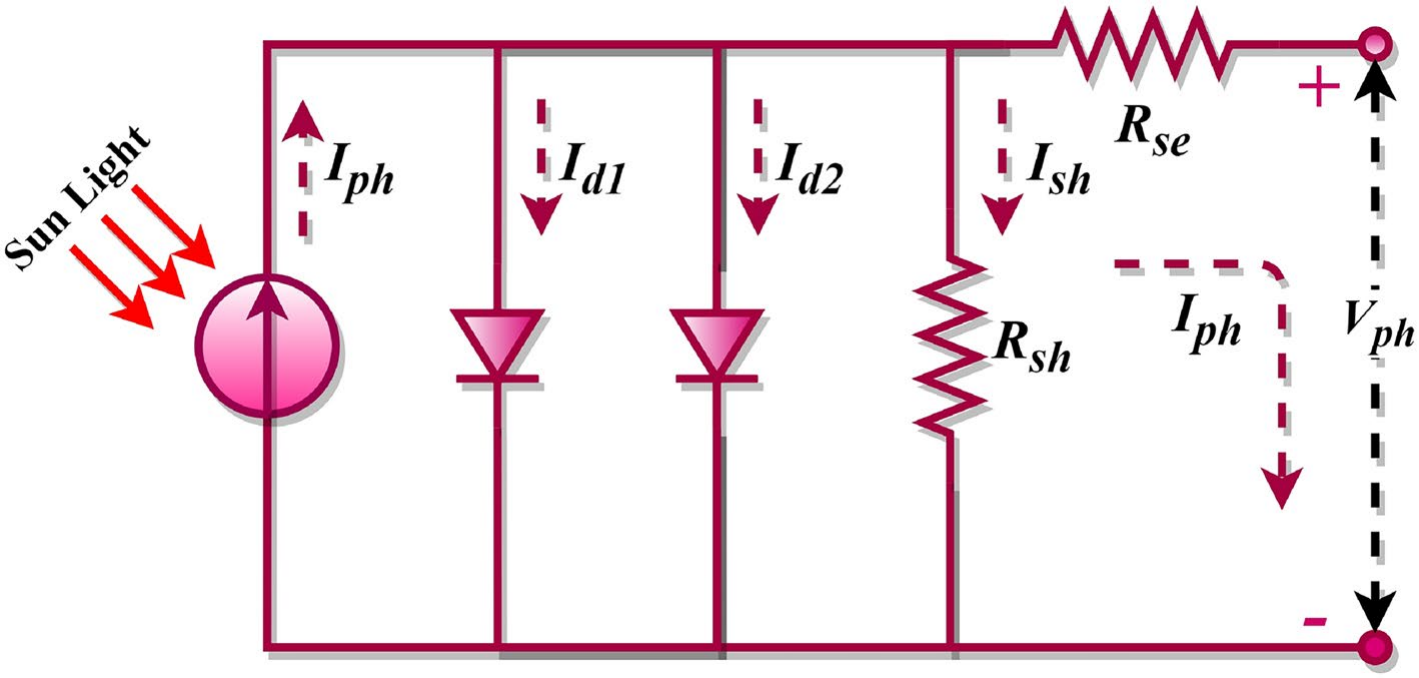
DDM of the photovoltaic cell.

The single-diode model is the most elementary and prevalently employed model for assessing PV systems. This model encompasses one semiconductor junction and the subsequent photovoltaic effect. Despite its simplicity, this model can effectively depict the function of a solar cell across diverse operational conditions. In the framework of the SDM, we engage a sole non-ideal diode, integrated into parallel alignment with the current source, a scenario visually represented in Fig. 1. As was broached earlier, ${R}_{sh}$ symbolizes the parallel resistance, ${R}_{se}$ stands for the series resistance, and the current traversing through the diode is designated as ${I}_{d}$. However, a contrasting approach is adopted in the DDM, wherein a pair of imperfect diodes are collaboratively connected in a parallel manner to one unique current source, a setup graphically elucidated in Fig. 2. In this configuration, ${I}_{d1}$ and ${I}_{d2}$ denote the currents that flow through the first and second diodes, respectively, with ${I}_{d2}$ in particular, corresponding to losses experienced within the space charge region. The fundamental mathematical formula for the single-diode model is as follows^84,85^.

1 $${I}_{pv}={I}_{ph}-{I}_{d}-{I}_{sh}$$

The diode current ${I}_{d}$ is deduced using the Shockley diode formula, and the leakage current ${I}_{sh}$ is attributed to the shunt resistance, which reflects power losses in the PV cell. By including the expressions for ${I}_{d}$ and ${I}_{sh}$, Eq. (1) is expanded as follows.

2 $${I}_{pv}={I}_{ph}-{I}_{sd}\left({\text{exp}}\left(\frac{{V}_{pv}+{I}_{pv}{R}_{se}}{n{V}_{t}}\right)-1\right)-\frac{{V}_{pv}+{I}_{pv}{R}_{se}}{{R}_{sh}}$$

3 $${V}_{t}=\frac{kT}{q}$$

where ${I}_{sd}$ represents the diode saturation current, ${V}_{pv}$ denotes the PV cell output voltage, ${I}_{pv}$ denotes the PV cell output current, $n$ denotes the ideality factor, ${V}_{t}$ is the thermal voltage, $k$ denotes Boltzmann’s constant, $q$ denotes the electron charge, and $T$ denotes the cell temperature.

Examining Eq. (2) reveals five undetermined variables: ${I}_{ph}$, $n$, ${I}_{sd}$, ${R}_{se}$, and ${R}_{sh}$. Accurate estimation of these parameters from the I–V characteristic of the PV cell is crucial for successful PV modelling. However, the SDM does not consider losses in the depletion region. This has led to the proposition of the DDM by researchers who have asserted that the DDM yields more precise results. The DDM is a more intricate model accounting for recombination losses in both the depletion and quasi-neutral regions of the solar cell, phenomena not represented in the SDM. The equivalent circuit of the DDM is shown in Fig. 2. Essentially, it embodies two single-diode models functioning in parallel, hence “double-diode”. The equation for the DDM becomes more sophisticated, incorporating two diode currents (${I}_{d1}$ and ${I}_{d2}$) is as follows^86^.

4 $${I}_{pv}={I}_{ph}-{I}_{d1}-{I}_{d2}-{I}_{sh}$$

By replacing the expression for ${I}_{d1}$ and ${I}_{d2}$ using the Shockley equation, Eq. (4) is rewritten as follows.

5 $${I}_{pv}={I}_{ph}-{I}_{sd1}\left({\text{exp}}\left(\frac{{V}_{pv}+{I}_{pv}{R}_{se}}{{n}_{1}{V}_{t}}\right)-1\right)-{I}_{sd2}\left({\text{exp}}\left(\frac{{V}_{pv}+{I}_{pv}{R}_{se}}{{n}_{2}{V}_{t}}\right)-1\right)-\frac{{V}_{pv}+{I}_{pv}{R}_{se}}{{R}_{sh}}$$

where ${I}_{sd1}$ and ${I}_{sd2}$ are the saturated currents of diode 1 and diode 2, respectively, and ${n}_{1}$ and ${n}_{2}$ are the ideality factors of diode 1 and diode 2, respectively.

**Figure 3:**
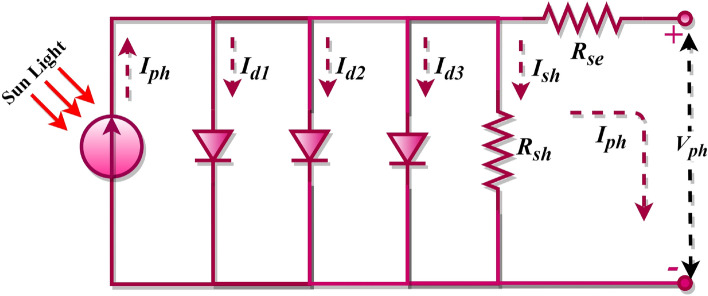
TDM of the photovoltaic cell.

Examining Eq. (5) reveals seven undetermined variables: ${I}_{ph}$, ${n}_{1}$, ${n}_{2}$, ${I}_{sd1}$, ${I}_{sd2}$, ${R}_{se}$, and ${R}_{sh}$. Accurate estimation of these parameters from the I–V characteristic of the PV cell is crucial for successful PV modelling. Each diode current in DDM possesses its ideality factor, which enhances the precision of this model and its complexity compared to the single-diode model. The TDM incorporates a third diode to signify the recombination losses in the space-charge region, consequently achieving an even more precise representation of the actual behaviour of the solar cell. The equivalent circuit of the TDM is shown in Fig. 3. However, it also significantly elevates the complexity of the model, with the formula involving three diode currents $({I}_{d1}$, ${I}_{d2}$ and ${I}_{d3}$) is as follows^87,88^.

6 $${I}_{pv}={I}_{ph}-{I}_{d1}-{I}_{d2}-{I}_{d3}-{I}_{sh}$$

By replacing the expression for ${I}_{d1}$, ${I}_{d2}$ and ${I}_{d3}$ using the Shockley equation, Eq. (6) is rewritten as follows.

7 $${I}_{pv}={I}_{ph}-{I}_{sd1}\left({\text{exp}}\left(\frac{{V}_{pv}+{I}_{pv}{R}_{se}}{{n}_{1}{V}_{t}}\right)-1\right)-{I}_{sd2}\left({\text{exp}}\left(\frac{{V}_{pv}+{I}_{pv}{R}_{se}}{{n}_{2}{V}_{t}}\right)-1\right)-{I}_{sd3}\left({\text{exp}}\left(\frac{{V}_{pv}+{I}_{pv}{R}_{se}}{{n}_{3}{V}_{t}}\right)-1\right)-\frac{{V}_{pv}+{I}_{pv}{R}_{se}}{{R}_{sh}}$$

where ${I}_{sd1}$, ${I}_{sd2}$ and ${I}_{sd3}$ are the saturated currents of diode 1, diode 2, and diode 3, respectively, and ${n}_{1}$, ${n}_{2}$, and ${n}_{3}$ are the ideality factors of diode 1, diode 2, and diode 3, respectively. Examining Eq. (7) reveals nine undetermined variables: ${I}_{ph}$, ${n}_{1}$, ${n}_{2}$, ${n}_{3}$, ${I}_{sd1}$, ${I}_{sd2}$, ${I}_{sd3}$, ${R}_{se}$, and ${R}_{sh}$. Accurate estimation of these parameters from the I–V characteristic of the PV cell is crucial for successful PV modelling. Each diode current in the TDM also has its ideality factor. Despite being the most precise model, its complexity can lead to more challenging parameter extraction and increased computational burden.

These models support the comprehension and prediction of PV cells' behaviour under diverse illumination and temperature conditions. The SDM is ideal for applications where simplicity and computational speed are key, whereas the DDM and TDM offer greater accuracy and detail and are more suited for in-depth research and comprehensive analysis of PV cell operations. Building a PV module around the principles of the SDM involves integrating SDM-based PV cells arranged in series and parallel networks as key components. A PV module is structured using multiple parallel strings of PV cells (${N}_{sh}$), each containing an equivalent number of series-aligned PV cells (${N}_{se}$). The I–V characteristics of the module are derived using Eq. (8).

8 $${I}_{pv}={I}_{ph}{N}_{sh}-{I}_{sd}{N}_{sh}\left({\text{exp}}\left(\frac{q\left(\frac{{V}_{pv}}{{N}_{se}}+\frac{{I}_{pv}{R}_{se}}{{N}_{sh}}\right)}{akT}\right)-1\right)-\frac{\frac{{V}_{pv}{N}_{sh}}{{N}_{se}}+{I}_{pv}{R}_{se}}{{R}_{sh}}$$

### Problem formulation

The PV system, with its nonlinear, intuitive, and transcendental traits, makes the PV cells/modules an appealing choice for optimization tasks. An error function that assesses the precision of parameter estimates can feasibly be established by synthesizing Eqs. (2, 5, 7, and 8). When employing optimization algorithms for this task, creating an error or fitness function is a must; this function must be minimized to secure the best parameter estimation values. The selection of an error function is crucial since it has a substantial impact on the general effectiveness of the final model. Root mean square error (RMSE), as outlined in Eq. (12), is used as the objective function, providing a comprehensive overview of the performance of the estimated model across all characteristics.

9 $$RMSE\left(x\right)=\sqrt{\frac{1}{N}{{\sum }_{i=1}^{N}f\left({V}_{pv},{I}_{pv},x\right)}^{2}}$$

The corresponding functions for the SDM, DDM, and TDM, along with the solution vector, denoted as $x$, for each PV model, are presented as follows.

10 $$\left\{\begin{array}{l}{f}_{SDM}\left({V}_{pv},{I}_{pv},x\right)={I}_{ph}-{I}_{sd}\left({\text{exp}}\left(\frac{{V}_{pv}+{I}_{pv}{R}_{se}}{n{V}_{t}}\right)-1\right)-\frac{{V}_{pv}+{I}_{pv}{R}_{se}}{{R}_{sh}}\\ x=\langle {I}_{ph}, n, {I}_{sd}, {R}_{se}, {R}_{sh}\rangle \end{array}\right.$$

11 $$\left\{\begin{array}{l}{f}_{DDM}\left({V}_{pv},{I}_{pv},x\right)={I}_{ph}-{I}_{sd1}\left({\text{exp}}\left(\frac{{V}_{pv}+{I}_{pv}{R}_{se}}{{n}_{1}{V}_{t}}\right)-1\right)-{I}_{sd2}\left({\text{exp}}\left(\frac{{V}_{pv}+{I}_{pv}{R}_{se}}{{n}_{2}{V}_{t}}\right)-1\right)\\ -\frac{{V}_{pv}+{I}_{pv}{R}_{se}}{{R}_{sh}}\\ x=\langle {I}_{ph}, {n}_{1}, {n}_{2}, {I}_{sd1}, {I}_{sd2}, {R}_{se}, {R}_{sh}\rangle \end{array}\right.$$

12 $$\left\{\begin{array}{l}{f}_{TDM}\left({V}_{pv},{I}_{pv},x\right)={I}_{ph}-{I}_{sd1}\left({\text{exp}}\left(\frac{{V}_{pv}+{I}_{pv}{R}_{se}}{{n}_{1}{V}_{t}}\right)-1\right)-{I}_{sd2}\left({\text{exp}}\left(\frac{{V}_{pv}+{I}_{pv}{R}_{se}}{{n}_{2}{V}_{t}}\right)-1\right)\\ -{I}_{sd3}\left({\text{exp}}\left(\frac{{V}_{pv}+{I}_{pv}{R}_{se}}{{n}_{3}{V}_{t}}\right)-1\right)-\frac{{V}_{pv}+{I}_{pv}{R}_{se}}{{R}_{sh}}\\ x=\langle {I}_{ph}, {n}_{1}, {n}_{2}, {n}_{3}, {I}_{sd1}, {I}_{sd2}, {I}_{sd3}, {R}_{se}, {R}_{sh}\rangle \end{array}\right.$$

By utilizing experimental data drawn from the I–V characteristic of the PV cell, it is possible to diminish the value of the RMSE. As a result, extracting parameters from the solar PV cell becomes a process geared towards reducing the RMSE value. This reduction is achieved by carefully adjusting the values of the solution variables. In other words, the goal of the extraction process is to find those values of the solution variables that would minimize the RMSE value, thus enhancing the accuracy of the PV model.

### Ethical approval

The authors have confirmed that no ethical approval is required.

## Proposed opposition-based exponential distribution optimizer

This section briefly introduces the concepts of the EDO algorithm and its mathematical modelling. Later, the discussion has been extended to formulating the proposed algorithm.

### Exponential distribution optimizer

The exponential distribution optimizer (EDO) is a metaheuristic algorithm rooted in mathematics that addresses intricate optimization problems^79^. The algorithm is founded on Exponential Distribution, a specific form of the continuous probability distribution that models the time between events in a Poisson point process. EDO effectively solves diverse optimization problems, including continuous, linear, nonlinear, and constrained problems. The idea of EDO is to employ a population of individuals (solutions) to explore the search space. Each solution represents a point in the search space, and the objective function value determines the quality of the solution. The algorithm exploits the balance between exploration (global search) and exploitation (local search) to direct the search procedure towards the global optima. In the phase of exploitation, the EDO leverages three fundamental constituents intrinsic to the exponential probability distribution (EPD). These constituents encompass the memoryless attribute, the directive solution, and the exponential dispersion, all essential to retain the contemporaneity of the innovative solution. Conversely, during the phase of exploration, a model optimized based on two solutions, both derived from the ED present within the initial population, is selected. Concurrently, the arithmetic average solution is utilized to effectuate an update to the promising solution.

The main inspiration of EDO, the ED, is a continuous probability distribution that describes the time between events in a Poisson point process, i.e., a process in which events occur continuously and independently at a constant average rate. The probability density function (PDF) of an exponential distribution is given by:

13 $$f\left(x\right)=\lambda {e}^{-\lambda x} {\text{for}} x\ge 0, \lambda =0$$

where $\lambda =\frac{1}{Mean}$. The ED has the property of being memoryless. In the context of the EDO algorithm, this implies that the quality of a solution does not depend on how the solution was obtained in previous iterations. In adherence to the memoryless property, there is no retention or consideration of the prior history of the solutions. This is due to the independence of past failures, rendering them empty of any impact on subsequent outcomes. Updated solutions are duplicated into the memoryless matrix to emulate the memoryless attribute intrinsic to the ED, irrespective of their fitness levels. This is premised on the understanding that historical data doesn’t influence future developments. Consequently, the memoryless matrix becomes a repository for two categories of solutions: successful ones and those that fall short. Initially, the memoryless matrix is assigned a value identical to the original population, designated as ${X}_{w}$. Like all metaheuristic algorithms, the initialization phase is done random population solution, i.e., the populations are randomly aligned, and the solutions are generated randomly using the random populations. Equation (14) can be employed to stochastically generate each exponential random parameter that is part of the candidate ED within the domain of the problem.

14 $${X}_{{w}_{i,j}}=lb+rand\cdot \left(ub-lb\right), i=\mathrm{1,2},\dots ,{N}_{p} {\text{and}} j=\mathrm{1,2},\dots ,dim$$

where $lb$ and $ub$ denote the lower and upper bounds of the control vectors, $rand$ denotes the uniform random number varies between [0,1], ${N}_{p}$ signifies the population size, and $dim$ denotes the problem dimension.

The exploitation stage leverages numerous features of the ED model, including the memoryless attribute, mean, standard deviation, and exponential rate. Furthermore, a directive solution is utilized to steer the exploration phase towards the global optima. The vicinity of a proficient solution often holds the potential for identifying the global optima. This is why several procedures delve into the search bounds near effective solutions by drawing in the lesser-performing ones. Hence, the quest for the global optima is centred on the guiding solution ${X}_{g}$. The ${X}_{g}$ is characterized as the average of the top three solutions from an organized population, designed as follows:

15 $${{X}_{g}}^{t}=\frac{{X}_{{w}_{best1}}^{t}+{X}_{{w}_{best2}}^{t}+{X}_{{w}_{best3}}^{t}}{3}$$

where $t$ represents the current iterations, the guiding solution is chosen over the best solution because it incrementally leads the solutions towards the optimum one. Even if the best solution is ensnared in local optimum, all other outcomes persistently gravitate towards this best solution. The area surrounding an effective solution often harbours the potential for uncovering the global optimum. Consequently, numerous algorithms take advantage of the search space near high-performing solutions by drawing in the less successful ones. The exploitation stage of the EDO practices several features of the ED model, such as the memoryless feature, mean, and standard variance, to update the solution as follows.

16 $${V}_{i}^{t+1}=\left\{\begin{array}{c}a.\left({ML}_{i}^{t}-{\sigma }^{2}\right)+b.{X}_{g}^{t}, if {X}_{{w}_{i}}^{t}={ML}_{i}^{t} \\ b.\left({ML}_{i}^{t}-{\sigma }^{2}\right)+{\text{log}}\left(\varphi \right).{X}_{{w}_{i}}^{t}, otherwise\end{array}\right.$$

17 $$b={f}^{5}$$

18 $$a={f}^{10}$$

19 $$f=2\times rand-1$$

where ${ML}_{i}^{t}$ denotes the *i*^th^ solution of the memoryless matrix, $\varphi$ signifies the uniform random number between [0, 1], $b$ and $a$ denote adaptive variables and $f$ denotes the random number in the range of [− 1, 1]. The expression for exponential variance is provided as follows.

20 $${\sigma }^{2}=\frac{1}{{\lambda }^{2}}$$

21 $$\lambda =\frac{1}{\mu }$$

22 $$\mu =\frac{{ML}_{i}^{t}+{X}_{g}^{t}}{2}$$

The exponential mean $\mu$ is determined as the mean value between the ${X}_{g}^{t}$ and the $i$th memoryless parameter, which could either be a winning or a losing solution.

The algorithm's exploration stage pinpoints areas within the search bound that are considered likely to contain the globally optimal results. The optimization framework for the EDO's exploration stage is constructed around two successful solutions from the initial agents in adherence to the ED. Following this, the solution is updated using Eqs. (23–27).

23 $${V}_{i}^{t+1}={X}_{{w}_{i}}^{t}-{M}^{t}+\left(c\cdot {Y}_{1}+\left(1-c\right)\cdot {Y}_{2}\right)$$

24 $${M}^{t}=\frac{1}{{N}_{p}}\sum_{i=1}^{{N}_{p}}{X}_{{w}_{j,i}}^{t}, j=\mathrm{1,2},\dots ,{\text{dim}}$$

25 $$c=\left(1-\frac{t}{T}\right)\times f$$

26 $$\left.\begin{array}{c}{Y}_{1}=M-{D}_{1}+{D}_{2}\\ {Y}_{2}=M-{D}_{2}+{D}_{1}\end{array}\right\}$$

27 $$\left.\begin{array}{c}{D}_{1}=M-{X}_{{w}_{rand1}}^{t}\\ {D}_{2}=M-{X}_{{w}_{rand2}}^{t}\end{array}\right\}$$

where ${M}^{t}$ denotes the mean solutions obtained in the actual population, $c$ denotes the tunable parameter signifying the ratio of information shared among ${Y}_{2}$ and ${Y}_{1}$ to the current solution, $T$ denotes the maximum number of iterations, and ${X}_{{w}_{rand1}}^{t}$ and ${X}_{{w}_{rand2}}^{t}$ denote the winners concerning the randomly selected ED from the population. An approach based on a greedy method is utilized for the solution update obtained during the exploration and exploitation stages within the initial population. Any updated solution is integrated into the original population only if it meets the criteria of being considered good. The pseudocode of the EDO algorithm exists in Algorithm 1.

**Algorithm 1:**
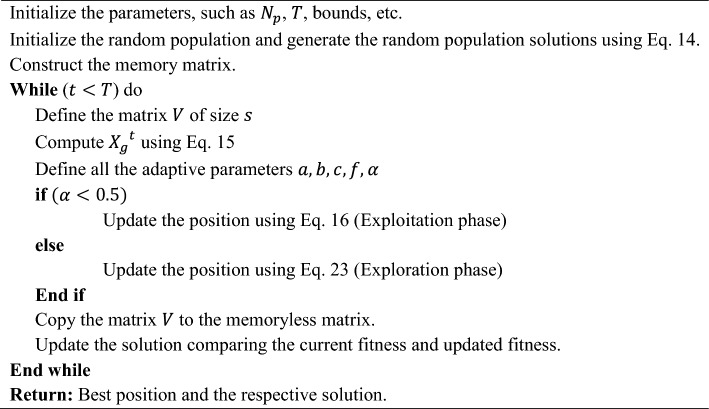
Pseudocode of the EDO algorithm.

### Opposition-based exponential distribution optimizer

The mutation operations in the EDO algorithm are based on the ED parameters and two control parameters ($a$ and $b$) derived from a uniformly distributed random variable f. This ensures diversity in the population and facilitates exploration and exploitation of the search space. Opposition-based learning (OBL) is an additional strategy that can be incorporated into EDO to enhance performance^80,89^. The foundational concept of OBL is based on De Morgan's laws in Boolean logic, which state that the complement of a conjunction is the disjunction of the complements, and the complement of a disjunction is the conjunction of the complements. It is used to calculate the “opposite” of a solution.

In optimization algorithms, a point and its opposite define an axis that bisects the solution space. The point lies on one side of the space, and its opposite is equidistant from the centroid but on the opposite side of the space. This geometric interpretation offers the key insight of OBL: for each point considered by the algorithm, there exists an unexplored point that is as far from the centroid as the original point but in the opposite direction. If a solution is a point in the search space, its opposite is another point in the space such that the line connecting the solution and its opposite passes through the centroid of the search space. The primary advantage of considering both a solution and its opposite is that it can double the useful information obtained from each fitness evaluation. Furthermore, because the opposite points are spread throughout the solution space, OBL can widen the search scope, enhancing exploration capabilities and accelerating convergence to the global optimum. Therefore, the proposed algorithm can explore the search space more effectively by evaluating both a solution and its opposite. The OBL is a strategy used to enhance the performance of optimization algorithms by exploring the search space more efficiently. The central concept of OBL is the simultaneous consideration of an estimate (a solution) and its opposition during the search process. In mathematical terms, for a problem defined in the $dim$-dimensional real space, a point ${X}_{w}$ in the search space is represented as a vector of $dim$ real numbers. Given a real-valued solution “${X}_{w}$” in the interval $[lb, ub]$, the opposite solution “${X}_{w,opp}$” is calculated as follows.

28 $${X}_{w}=\left[{{X}_{w}}_{1},{{X}_{w}}_{2},\dots ,{{X}_{w}}_{dim}\right]$$

29 $${X}_{w,opp}=lb+ ub-{X}_{w}$$

where $lb$ and $ub$ represent the lower and upper bounds of the search space, respectively. The fitness of both ${X}_{w}$ and ${X}_{w,opp}$ are evaluated, and the point with the better fitness is selected, i.e., ${\text{if}} f({X}_{w,opp}) < f({X}_{w}), {\text{then}} {X}_{w} = {X}_{w,opp}$.

**Figure 4:**
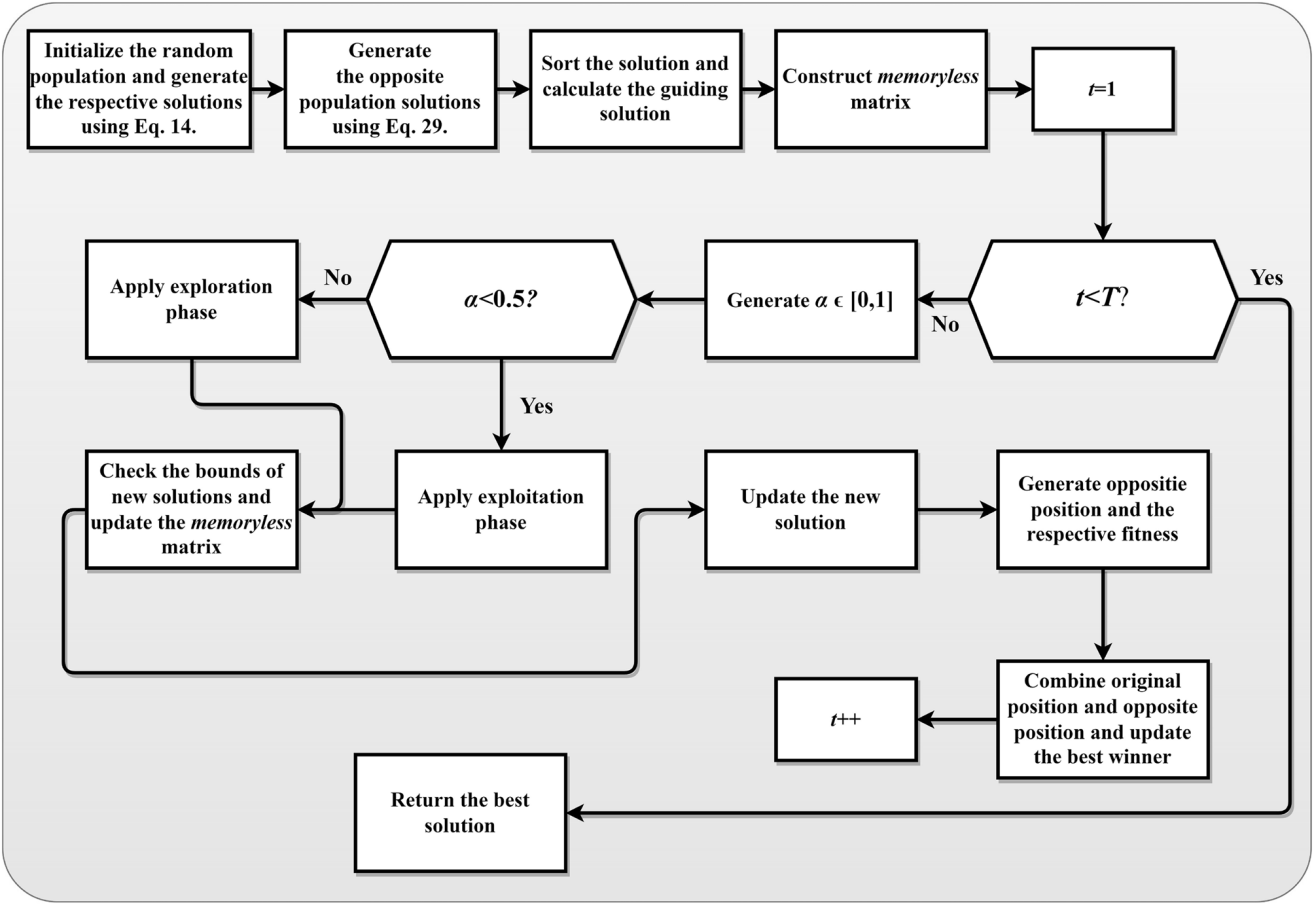
Flowchart of the proposed OBEDO algorithm.

The main idea of this strategy is to consider the current estimate and its opposite to obtain a better approximation for the global optimal solution. In other words, for each candidate solution, the algorithm generates its opposite solution and evaluates both. The algorithm tries to keep the one with better fitness for the next generation. This can be especially useful in the initial stages of the algorithm's run, where it can significantly increase the convergence speed. The reason is that, with OBL, the algorithm can simultaneously consider two “opposite” points in the solution space. This is akin to exploring two different directions simultaneously, potentially leading to a broader and more efficient search. This OBL strategy is incorporated into EDO during the initialization and mutation phases. As discussed, during initialization, each randomly generated solution ${X}_{w}$ is accompanied by its opposite ${X}_{w,opp}$, and the one with better fitness is selected for the initial population. An offspring solution is created, and its opposite is generated during mutation. Again, the one with the better fitness is selected to replace its parent. This way, in each iteration, the algorithm explores the vicinity of the solutions and their opposite points in the search space. This strategy significantly enhances the exploratory capability of the algorithm, making it more robust and potentially faster in finding the global optimal solution. By applying this strategy, the OBEDO can benefit from an enhanced exploration capability, potentially improving its effectiveness and efficiency in solving complex optimization problems. The pseudocode of the proposed OBEDO algorithm is presented in Algorithm 2. The flowchart of the OBEDO is shown in Fig. 4.

**Algorithm 2:**
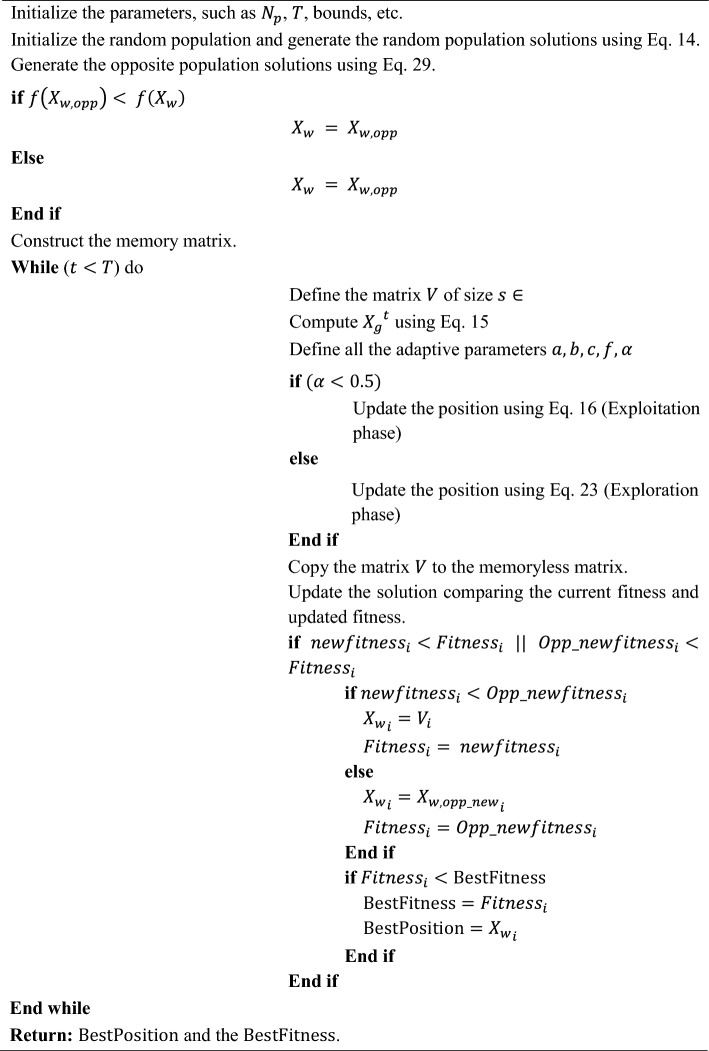
Pseudocode of the proposed OBEDO algorithm.

The OBEDO algorithm, as discussed, likely integrates two key concepts: OBL and EDO algorithm. OBL is a concept used in optimization algorithms to speed up the convergence rate. It works on the principle that considering a candidate solution and its opposite can provide a better approximation of the global optimum. In OBL, for every estimated solution, the opposite solution is also considered. This means if a solution is at a point $x$ in the search space, its opposite $-x$ is also evaluated. This approach increases the chances of finding better solutions in fewer iterations as it explores the search space more effectively. When integrated into the OBEDO framework, OBL could help in quickly identifying more promising regions of the search space for parameter estimation, thereby improving the efficiency and accuracy of the optimization process. OBL enhances the global search capability, ensuring diverse and comprehensive exploration, while the exponential distribution method fine-tunes the search, allowing for efficient exploitation of promising areas. This synergy could make OBEDO particularly effective in navigating complex, high-dimensional search spaces typical in solar PV model parameter estimation, where traditional methods might struggle due to local optima or slow convergence. The effectiveness of OBEDO in outperforming other methods could stem from its ability to balance exploration and exploitation in the optimization process. This balance is crucial in parameter estimation problems, where finding the global optimum in a complex search space is essential for accurate and reliable results.

### Complexity of the proposed OBEDO algorithm

The algorithm creates an initial population of solutions ${{X}_{w}}_{i}$. The complexity of the initialization phase should be $O({N}_{p}\times dim)$, where ${N}_{p}$ is the number of individuals, and $dim$ is the dimension of the solution space. The algorithm performs a series of computations for each individual in the population (${N}_{p}$), and this is done for each iteration. Thus, the complexity within the loop could be considered as $O({N}_{p}\times dim)$. This includes fitness computation, memory updates, and solution modifications. Assuming the complexity of the fitness function is $O(dim)$, this would still give a complexity of $O({N}_{p}\times dim)$ for the whole iteration. The sorting operations would add a complexity of $O({N}_{p}\times log({N}_{p}))$ per iteration. However, since ${N}_{p}\times log({N}_{p})$ is smaller than ${N}_{p}\times dim$ for large enough ${N}_{p}$ and $dim$, ignore it in the final complexity assessment. Therefore, the time complexity of the OBEDO can be approximated as $O({N}_{p}\times dim\times T)$, which is the same as the original EDO algorithm.

## Results and discussions

**Figure 5:**
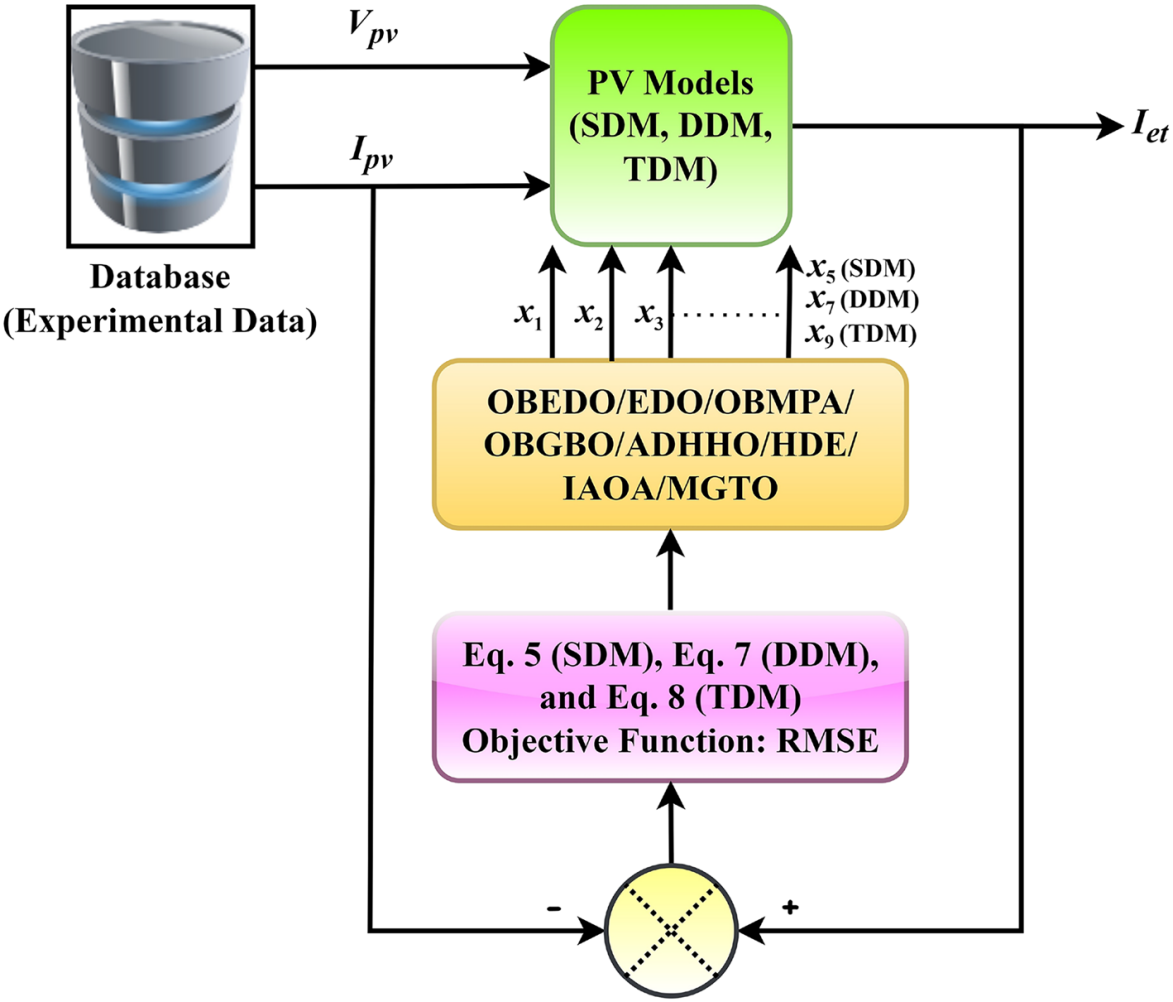
Application of the OBEDO and other algorithms for parameter estimation.

In this section of the paper, we present the experimental results obtained to assess the performance of the proposed OBEDO algorithm. The OBEDO determines the parameters of four benchmarked PV cell/module models: RTC France silicon cell, PVM752 GaAs PV cell, Photowatt PWM201 module, commercial Sharp ND-R250A5 PV module, and commercial SM55 PV module. To evaluate the effectiveness of the OBEDO, it is applied to three PV models, namely the SDM, DDM, and TDM. For each model, the OBEDO and other seven algorithms, such as opposition-based GBO (OBGBO), opposition-based marine predator algorithm (OBMPA), MGTO, ADHHO, IAOA, HDE, and original EDO, are employed to obtain the optimal parameters that best fit the experimental data. The application of the proposed OBEDO and other algorithms to estimate PV parameters is pictorially represented in Fig. 5.

For the RTC France silicon cell with a diameter of 57 mm, experimental data is collected under specific conditions, with a solar irradiance of 1000 W/m^2^ and a temperature of 33 °C. A total of 26 sets of current and voltage measurements are gathered to determine the cell variables by optimizing the RMSE, a common metric for evaluating the accuracy of models. The goal is to find the best-fitting parameters that minimize the RMSE and, thus, enhance the accuracy of the PV cell model. For the PVM752 GaAs PV cell, the experimental data is gathered under 1000W/m^2^ irradiation and 25 °C temperature. Similarly, for the Photowatt PWM201 module, experimental data is gathered under specific conditions with a solar insolation of 1000 W/m^2^ and a temperature of 45 °C. For the commercial Sharp ND-R250A5 PV module, the experimental data is gathered under 1040W/m^2^ irradiation and 59 °C temperature. In addition, the data for the commercial SM55 PV module under different operating conditions are also collected. To ensure realistic parameter estimates, $lb$ and $ub$ limits for the cells and the modules parameters are provided in Table 2. These limits constrain the optimization process during the execution of all algorithms and ensure that the resulting parameters fall within physically meaningful ranges.

**Table 2:** Limits of all the optimizing parameters of all PV cases.

Parameters	Scenario 1		Scenario 2		Scenario 3		Scenario 4		Scenario 5	
	$lb$	$ub$	$lb$	$ub$	$lb$	$ub$	$lb$	$ub$	$lb$	$ub$
${I}_{ph}$(A)	0	1	0	0.5	0	2	0	10	0	2 times of short-circuit current
$n$	1	2	1	2	1	50	1	120	1	5
${I}_{sd}$(µA)	0	1	0	1	0	50	0	10	0	100
${R}_{sh}$(Ω)	0	100	0	1000	0	2000	0	5500	0	5000
${R}_{se}$(Ω)	0	0.5	0	0.8	0	2	0	1	0	2
${I}_{sd1}$, ${I}_{sd2}$, and ${I}_{sd3}$ (µA)	0	1	0	1	0	50	–	–	–	–
${n}_{1}, {n}_{2}$, and ${n}_{3}$	1	5	1	5	1	50	–	–	–	–

The simulation tests were conducted on a laptop running Windows 11 with specific hardware specifications. The laptop has an Intel(R) Core (TM) i5-10300H CPU operating at a clock frequency of 2.44 GHz and 8 GB of RAM. For the simulation tests, this study considered a population size of 40, and the maximum number of iterations was set to 1000 for all the PV models under consideration. Additionally, other control parameters for all the algorithms utilized in the experiments are documented in Table 3, providing transparency and reproducibility of the study. To ensure a fair comparison among all the selected algorithms, each method was executed 30 times independently. This repetition helps account for any potential variability in the results and allows us to draw robust conclusions regarding the algorithms' performance. This study employed MATLAB R2020b software to conduct the simulation tests. By running the experiments on the specified laptop and using the common platform of MATLAB R2020b, this study ensures consistency and comparability of the results across all algorithms and PV models. This systematic approach allows us to make well-informed evaluations of the algorithms' efficiency and effectiveness in optimizing the PV model parameters.

**Table 3:** Parameters of all the algorithms.

Algorithms	Parameters	Ranges
OBEDO and EDO	$\alpha$	0.5
OBGBO	$\varepsilon$ $pr$	0.01 0.5
OBMPA	$FADs$ $p$	0.5 0.5
MGTO	$p$ $\beta$ $w$	0.03 3 0.8
ADHHO	$h$ $Pmax$ $Pmin$ $U$	6 5 2 [2,5]
HDE	$p$ $H$	0.1 $5$
IAOA	$\mu$ $\alpha$ ${MOP}_{Max}$ ${MOP}_{Min}$	0.499 5 1 0

In this study, the performance comparison among all the selected algorithms is carried out using various statistical measures and performance metrics to assess their effectiveness in optimizing the PV model parameters. The statistical measures considered include Minimum (Min), Maximum (Max), Mean, and Standard Deviation (STD). These measures help us understand the range and distribution of the results obtained by each algorithm. On the other hand, performance metrics provide specific quantitative evaluations of the algorithms' accuracy in predicting the PV model parameters. The metrics used in this comparison are Relative Error (RE), Integral Absolute Error (IAE), and Root Mean Square Error (RMSE). The runtime (RT) of each algorithm is also analyzed, which represents the average running time of all 30 independent runs for each case study. This information is crucial for understanding the computational efficiency of the algorithms and their feasibility for practical applications. The metrics RE and IAE are computed as follows: (i) IAE represents the integral absolute error for a particular trial, which is calculated as the absolute difference between the estimated current ${I}_{es}$ and the measured current ${I}_{ex}$ value for that trial, and (ii) RE denotes the relative error, which quantifies the percentage difference between the estimated current and the measured current value. It is computed as the ratio of the difference between the measured and estimated current to the measured current value. The IAE is designed to penalize errors equally, regardless of the direction, while the RE provides insight into the magnitude of the absolute error with respect to the measured data. When an exact measurement is unavailable, using the measured value to calculate relative inaccuracy is common. Finally, a Friedman's Ranking Test (FRT) is performed to validate the statistical significance of the results. This test allows for comparing multiple algorithms across different metrics and identifies whether any algorithm significantly outperforms the others. By employing these statistical measures, performance metrics, runtime analysis, and Friedman's Ranking Test, the study comprehensively evaluates the selected algorithms' performance and provides valuable insights into their capabilities in accurately estimating the PV model parameters. Such detailed comparisons are essential for researchers and practitioners to decide on the most suitable algorithm for specific PV modelling applications.

### Scenario 1—RTC France Si PV cell

This sub-section details the results obtained by the proposed algorithm and other algorithms for scenario 1, i.e., SDM, DDM, and TDM of the RTC France Si PV cell. The bounds for all the PV models are provided in Table 2. As deliberated earlier, all algorithms are executed 30 times for a fair comparison.

**Figure 6:**
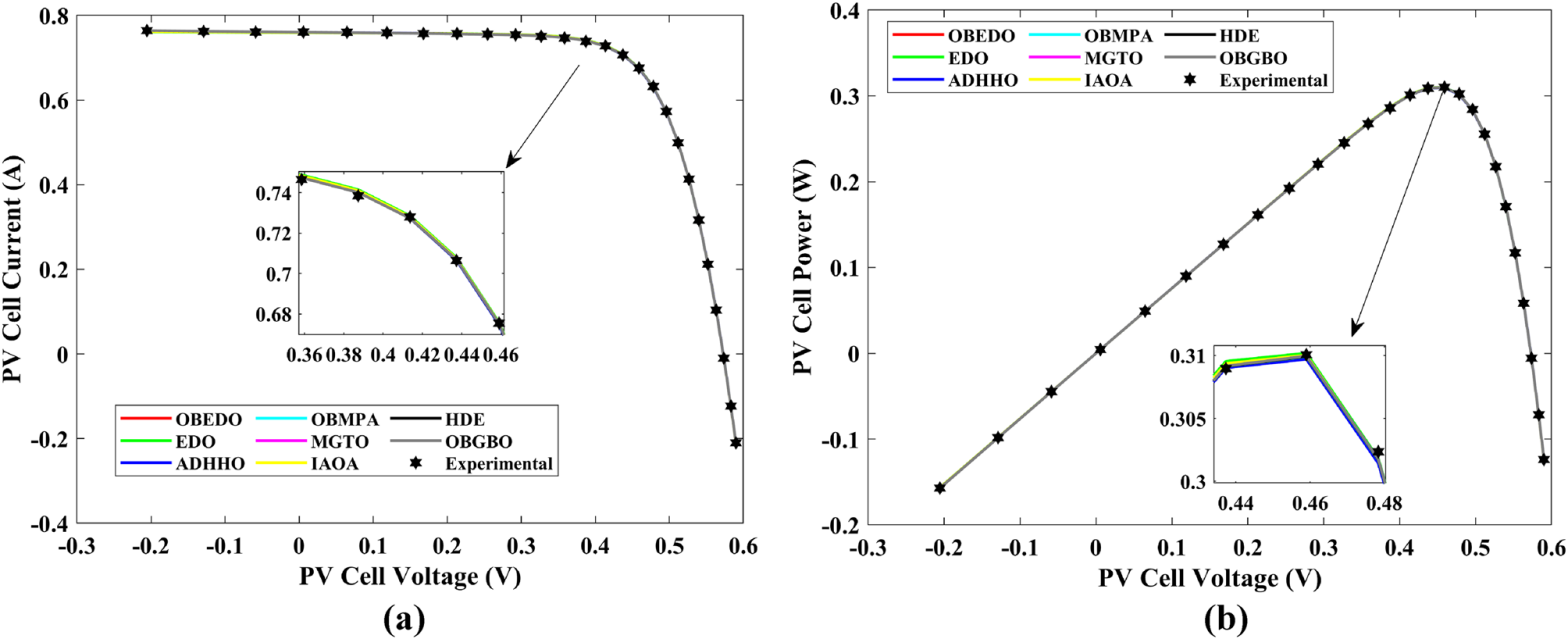
Characteristics curves obtained by algorithms for Scenario 1 (SDM); (**a**) I–V curves, (**b**) P–V curves.

**Figure 7:**
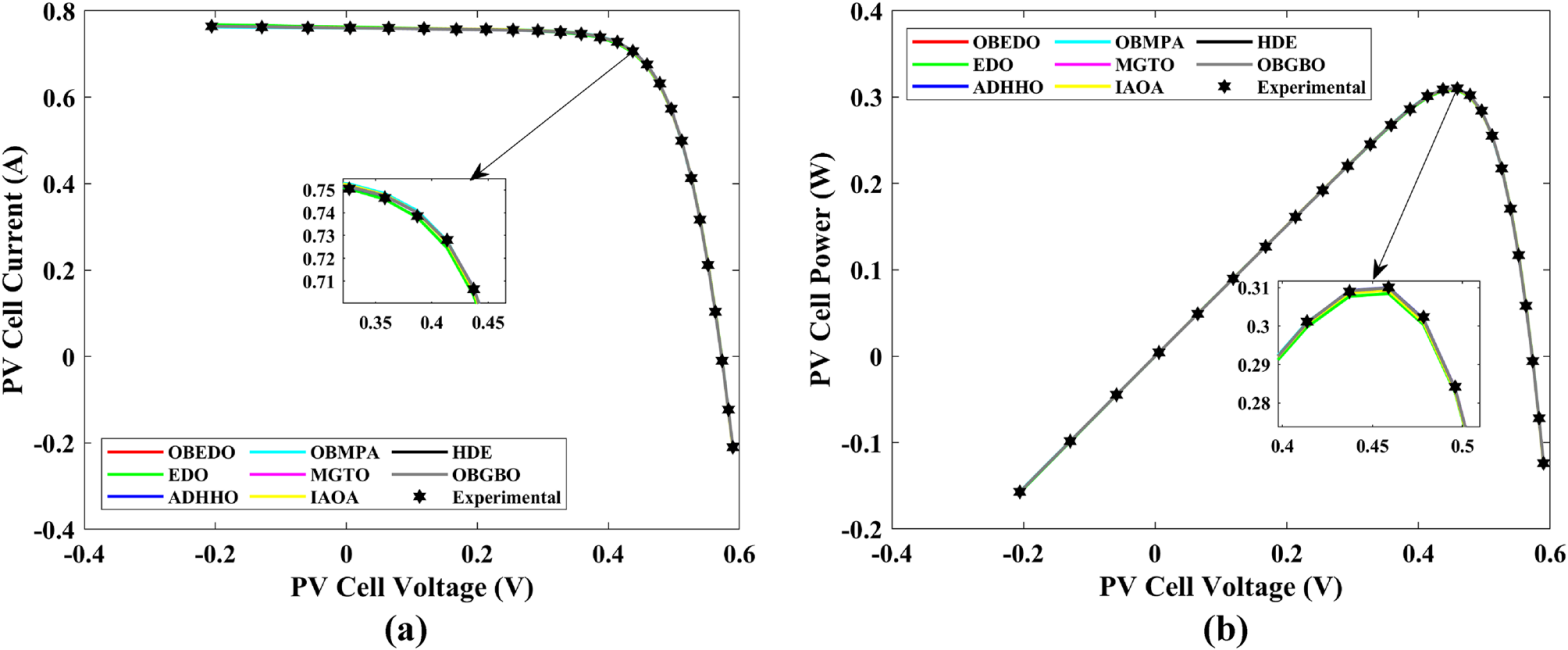
Characteristics curves obtained by algorithms for Scenario 1 (DDM); (**a**) I–V curves, (**b**) P–V curves.

**Figure 8:**
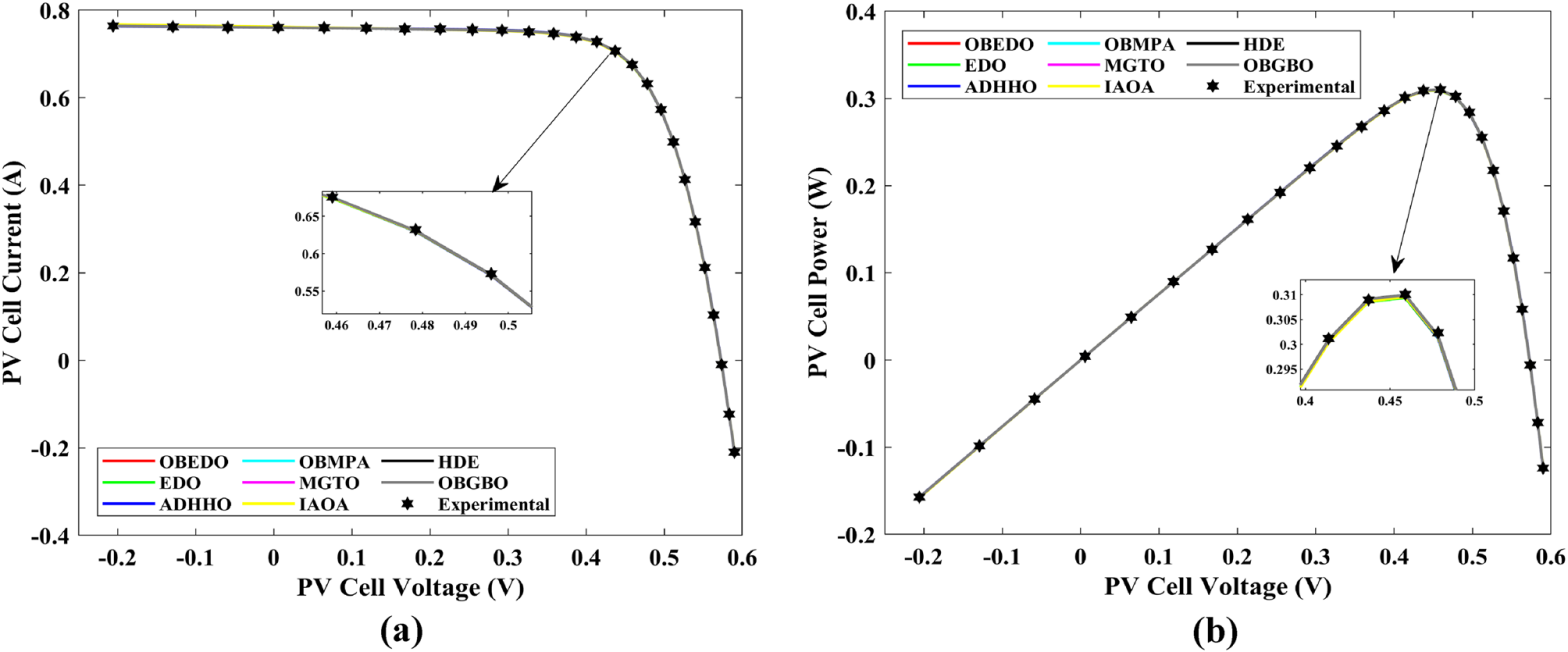
Characteristics curves obtained by algorithms for Scenario 1 (TDM); (**a**) I–V curves, (**b**) P–V curves.

**Figure 9:**
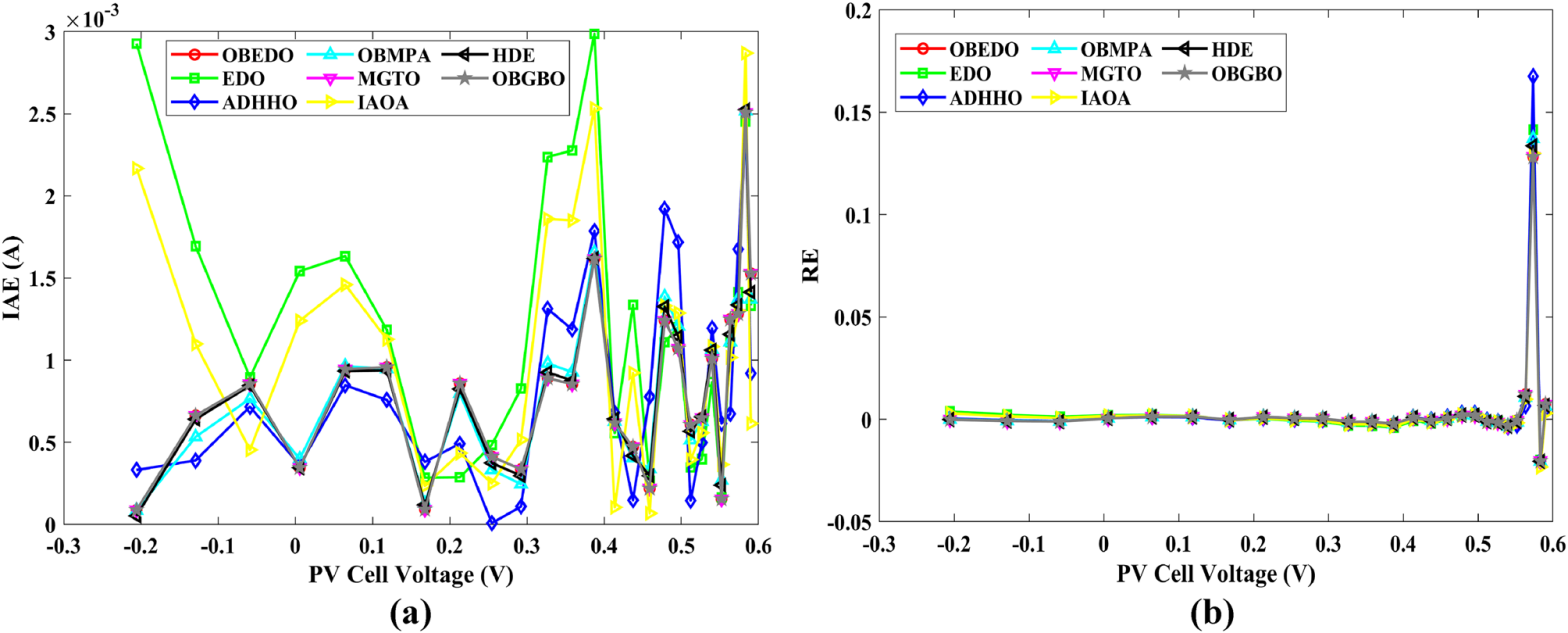
Error curves obtained by algorithms for Scenario 1 (SDM); (**a**) IAE, (**b**) RE.

**Figure 10:**
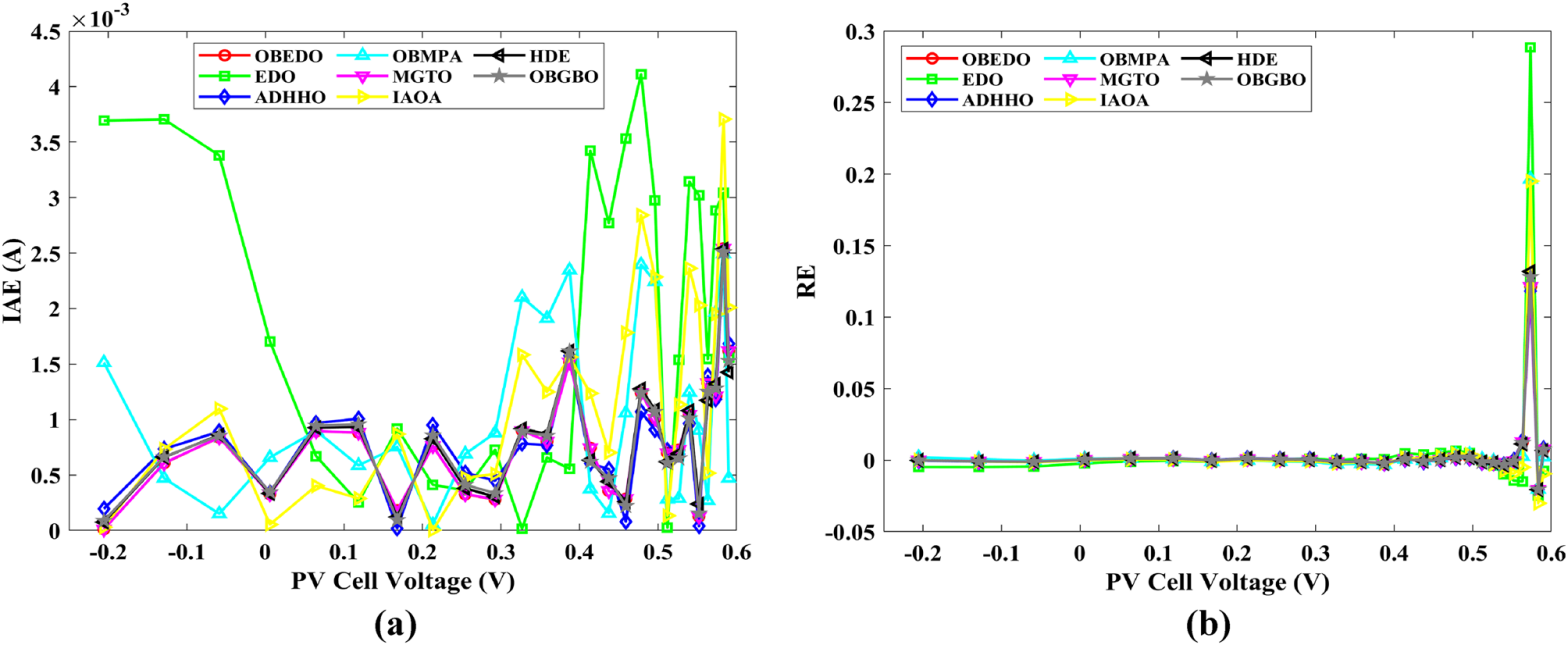
Error curves obtained by algorithms for Scenario 1 (DDM); (**a**) IAE, (**b**) RE.

**Figure 11:**
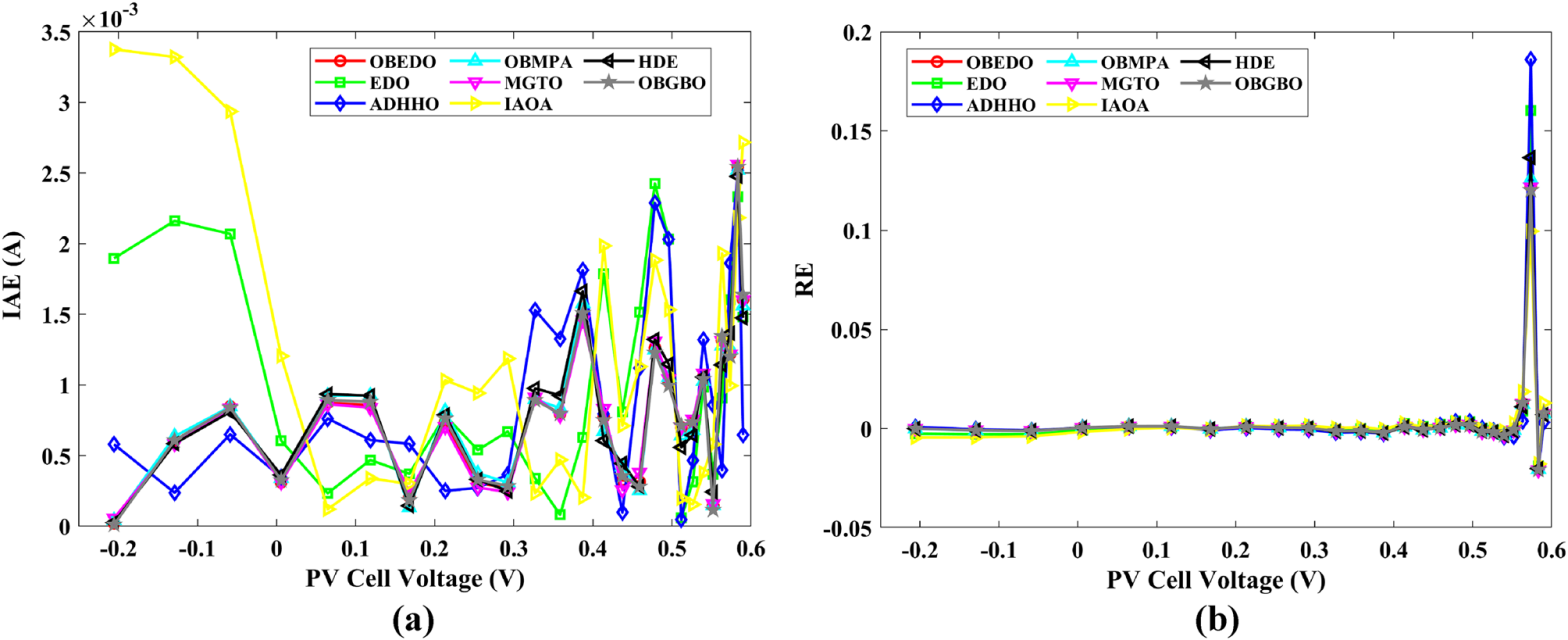
Error curves obtained by algorithms for Scenario 1 (TDM); (**a**) IAE, (**b**) RE.

Tables 4, 5, and 6 display a comprehensive overview of the five, seven, and nine parameters estimated through the SDM, DDM, and TDM employment pertaining to the RTC of France Si solar cell. It is imperative to emphasize that these parameters were determined within the precise confines, as elucidated in Table 2. A graphical representation of the I–V (Current–Voltage) and P–V (Power–Voltage) characteristic curves pertinent to the SDM, DDM, and TDM of the RTC France Si solar cell obtained by all algorithms have been expounded in Figs. 6, 7, and 8. These elucidate the outcomes engendered by the proposed OBEDO that was instituted as part of this study. It is crucial to underline that the quantitative values encapsulating the IAE and the RE have been meticulously documented in Table 7. To visually portray the paramount significance of the RE and IAE values ascertained through the instrumentality of the OBEDO and other algorithms, this study has deviously created error graphs as showcased in Figs. 9, 10, and 11. A cursory examination of Figs. 6, 7, 8, 9, 10, and 11 unfolds a noticeably harmonious alignment between the projected estimated and empirically derived experimental curves. This alignment strengthens the inference that the curve fitting has yielded a commendable equivalence. To offer a comprehensive assessment, meticulous cataloguing of statistical indicators including, but not limited to, Min, Mean, Max, RT, and STD values has been meticulously undertaken. These values have been scrupulously logged within Table 8 to facilitate in-depth data comprehension. Furthermore, it is noteworthy that salient achievements, exemplified by the most remarkable outcomes gleaned from the diverse tables, have been judiciously highlighted through the distinctive formatting of boldfaces.

**Table 4:** Parameters estimated for Scenario 1 (SDM).

Algorithms	${I}_{ph}$(A)	${I}_{sd}$(A)	${R}_{se}$(Ω)	${R}_{sh}$(Ω)	$n$	RMSE
OBEDO	0.7608	3.23E−07	0.0364	53.7185	1.4812	**9.8602E−04**
EDO	0.7593	4.04E−07	0.0358	99.9891	1.5036	1.4649E−03
ADHHO	0.7607	3.95E−07	0.0356	59.8812	1.5017	1.0585E−03
OBMPA	0.7607	3.38E−07	0.0362	55.4146	1.4859	9.9045E−04
MGTO	0.7608	3.23E−07	0.0364	53.7185	1.4812	**9.8602E**−**04**
IAOA	0.7597	4.01E−07	0.0356	82.1571	1.5030	1.2758E−03
HDE	0.7608	3.31E−07	0.0363	54.2152	1.4837	9.8731E−04
OBGBO	0.7608	3.23E−07	0.0364	53.7185	1.4812	**9.8602E**−**04**

Significant values are in [bold].

**Table 5:** Parameters estimated for Scenario 1 (DDM).

Algorithms	${I}_{ph}$(A)	${I}_{sd1}$(A)	${R}_{se}$(Ω)	${R}_{sh}$(Ω)	${n}_{1}$	${I}_{sd2}$(A)	${n}_{2}$	RMSE
OBEDO	**0.7608**	**2.31E−07**	**0.0367**	**55.3995**	**1.4529**	**7.08E−07**	**2.0000**	**9.8250E−04**
EDO	0.7630	8.76E−08	0.0344	38.5036	1.4050	8.51E−07	1.7015	2.3699E−03
ADHHO	0.7608	1.20E−13	0.0366	52.3503	1.5000	3.07E−07	1.4760	9.9082E−04
OBMPA	0.7602	1.95E−07	0.0349	79.9286	1.4771	3.63E−07	1.6015	1.3081E−03
MGTO	**0.7608**	**2.31E−07**	**0.0367**	**55.3762**	**1.4530**	**7.04E**−**07**	**2.0000**	**9.8250E**−**04**
IAOA	0.7611	3.26E−07	0.0341	61.8652	1.6646	3.09E−07	1.4993	1.5329E−03
HDE	0.7608	3.22E−07	0.0363	54.0502	1.4812	4.06E−08	2.0000	9.8650E−04
OBGBO	0.7608	3.21E−07	0.0364	53.7417	1.4806	1.08E−08	1.8916	9.8593E−04

Significant values are in [bold].

**Table 6:** Parameters estimated for Scenario 1 (TDM).

Algorithms	${I}_{ph}$(A)	${I}_{sd1}$(A)	${R}_{se}$(Ω)	${R}_{sh}$(Ω)	${n}_{1}$	${I}_{sd2}$(A)	${n}_{2}$	${I}_{sd3}$(A)	${n}_{3}$	RMSE
OBEDO	**0.7608**	**5.88E−07**	**0.0367**	**55.7780**	**1.9995**	**2.34E−07**	**1.4537**	**9.79E−07**	**2.7316**	**9.8082E−04**
EDO	0.7619	2.79E−07	0.0362	44.1202	1.4698	1.69E−07	1.9986	5.03E−07	2.1237	1.2949E−03
ADHHO	0.7607	3.67E−07	0.0353	64.6790	1.8994	3.48E−07	1.4934	4.04E−12	3.4918	1.1473E−03
OBMPA	0.7608	2.81E−07	0.0365	54.4616	1.4695	0.00E+00	1.9747	3.07E−07	2.0000	9.8381E−04
MGTO	0.7608	9.96E−07	0.0368	56.2894	1.9998	2.02E−07	1.4419	2.09E−12	4.9960	9.8353E−04
IAOA	0.7626	5.17E−07	0.0376	37.2626	1.7823	1.30E−07	1.4081	6.24E−09	3.1122	1.5865E−03
HDE	0.7607	5.80E−10	0.0363	55.0944	1.7439	3.32E−07	1.4839	1.00E−06	4.1292	9.8732E−04
OBGBO	0.7608	2.23E−07	0.0368	55.4366	1.4501	6.97E−07	1.9772	1.00E−06	4.9548	9.8244E−04

Significant values are in [bold].

**Table 7:** RE and IAE values attained by OBEDO for Scenario 1.

${V}_{ex}$	${I}_{ex}$	SDM	DDM	TDM	SDM	DDM	TDM	SDM	DDM	TDM
		${I}_{es}$(A)			$RE$			$IAE$(A)		
− 0.2057	0.7640	0.7641	0.7640	0.7640	− 1.15E−04	1.58E−05	2.72E−05	8.77E−05	1.21E−05	2.08E−05
− 0.1291	0.7620	0.7627	0.7626	0.7626	− 8.70E−04	− 7.96E−04	− 7.97E−04	6.63E−04	6.06E−04	6.07E−04
− 0.0588	0.7605	0.7614	0.7613	0.7613	− 1.12E−03	− 1.10E−03	− 1.11E−03	8.55E−04	8.38E−04	8.47E−04
0.0057	0.7605	0.7602	0.7602	0.7602	4.55E−04	4.31E−04	4.10E−04	3.46E−04	3.28E−04	3.12E−04
0.0646	0.7600	0.7591	0.7591	0.7591	1.24E−03	1.18E−03	1.15E−03	9.45E−04	8.95E−04	8.74E−04
0.1185	0.7590	0.7580	0.7581	0.7581	1.26E−03	1.16E−03	1.13E−03	9.58E−04	8.83E−04	8.57E−04
0.1678	0.7570	0.7571	0.7572	0.7572	− 1.21E−04	− 2.43E−04	− 2.78E−04	9.17E−05	1.84E−04	2.11E−04
0.2132	0.7570	0.7561	0.7562	0.7563	1.13E−03	1.01E−03	9.72E−04	8.59E−04	7.61E−04	7.36E−04
0.2545	0.7555	0.7551	0.7552	0.7552	5.47E−04	4.32E−04	4.06E−04	4.13E−04	3.27E−04	3.06E−04
0.2924	0.7540	0.7537	0.7537	0.7537	4.46E−04	3.71E−04	3.55E−04	3.36E−04	2.80E−04	2.68E−04
0.3269	0.7505	0.7514	0.7514	0.7514	− 1.19E−03	− 1.20E−03	− 1.20E−03	8.91E−04	9.00E−04	9.01E−04
0.3585	0.7465	0.7474	0.7473	0.7473	− 1.14E−03	− 1.08E−03	− 1.07E−03	8.54E−04	8.06E−04	7.95E−04
0.3873	0.7385	0.7401	0.7400	0.7400	− 2.19E−03	− 2.05E−03	− 2.03E−03	1.62E−03	1.52E−03	1.50E−03
0.4137	0.7280	0.7274	0.7273	0.7272	8.49E−04	1.02E−03	1.06E−03	6.18E−04	7.45E−04	7.74E−04
0.4373	0.7065	0.7070	0.7069	0.7068	− 6.69E−04	− 5.05E−04	− 4.60E−04	4.73E−04	3.57E−04	3.25E−04
0.4590	0.6755	0.6753	0.6752	0.6752	3.25E−04	4.25E−04	4.67E−04	2.20E−04	2.87E−04	3.16E−04
0.4784	0.6320	0.6308	0.6308	0.6307	1.96E−03	1.96E−03	2.00E−03	1.24E−03	1.24E−03	1.26E−03
0.4960	0.5730	0.5719	0.5720	0.5720	1.87E−03	1.76E−03	1.78E−03	1.07E−03	1.01E−03	1.02E−03
0.5119	0.4990	0.4996	0.4997	0.4997	− 1.22E−03	− 1.40E−03	− 1.40E−03	6.07E−04	6.99E−04	6.99E−04
0.5265	0.4130	0.4136	0.4137	0.4137	− 1.57E−03	− 1.76E−03	− 1.79E−03	6.49E−04	7.28E−04	7.38E−04
0.5398	0.3165	0.3175	0.3175	0.3176	− 3.19E−03	− 3.30E−03	− 3.35E−03	1.01E−03	1.04E−03	1.06E−03
0.5521	0.2120	0.2122	0.2121	0.2121	− 7.31E−04	− 5.98E−04	− 6.80E−04	1.55E−04	1.27E−04	1.44E−04
0.5633	0.1035	0.1023	0.1022	0.1022	1.21E−02	1.28E−02	1.27E−02	1.25E−03	1.33E−03	1.31E−03
0.5736	− 0.0100	− 0.0087	− 0.0088	− 0.0088	1.28E−01	1.21E−01	1.22E−01	1.28E−03	1.21E−03	1.22E−03
0.5833	− 0.1230	− 0.1255	− 0.1255	− 0.1255	− 2.04E−02	− 2.07E−02	− 2.07E−02	2.51E−03	2.54E−03	2.55E−03
0.5900	− 0.2100	− 0.2085	− 0.2084	− 0.2084	7.27E−03	7.71E−03	7.64E−03	1.53E−03	1.62E−03	1.60E−03
Mean values					4.74E−03	4.50E−03	4.51E−03	8.28E−04	8.19E−04	8.18E−04

**Table 8:** Performance comparison for Scenario 1.

Model	Algorithms	$Min$	$Max$	$Mean$	$Median$	$STD$	$RT$
SDM	**OBEDO**	**9.8602E−04**	**9.8602E−04**	**9.8602E−04**	**9.8602E−04**	**4.7451E−17**	8.55
	EDO	1.4649E−03	7.9048E−03	3.0831E−03	2.1984E−03	2.2341E−03	**8.26**
	ADHHO	1.0585E−03	2.4392E−03	1.6272E−03	1.6022E−03	4.8327E−04	8.60
	OBMPA	9.9045E−04	1.5908E−03	1.3958E−03	1.4585E−03	2.1103E−04	8.93
	MGTO	**9.8602E−04**	**9.8602E−04**	**9.8602E−04**	**9.8602E−04**	2.2061E−14	8.79
	IAOA	1.2758E−03	4.0880E−02	7.8761E−03	4.1836E−03	1.1954E−02	9.37
	HDE	9.8731E−04	1.1025E−03	1.0436E−03	1.0448E−03	4.4832E−05	8.64
	OBGBO	**9.8602E−04**	9.8874E−04	9.8631E−04	9.8602E−04	8.5478E−07	78.29
DDM	**OBEDO**	**9.8250E−04**	1.4090E−03	1.0282E−03	**9.8505E−04**	1.3384E−04	7.77
	EDO	2.3699E−03	3.9190E−02	1.6836E−02	5.6876E−03	1.6494E−02	**7.02**
	ADHHO	9.9082E−04	2.5436E−03	1.9030E−03	1.9059E−03	4.7352E−04	7.97
	OBMPA	1.3081E−03	3.3392E−02	4.9581E−03	1.5680E−03	1.0007E−02	7.96
	MGTO	**9.8250E−04**	**1.1343E−03**	**1.0040E−03**	9.8551E−04	**4.7675E−05**	8.33
	IAOA	1.5329E−03	3.2453E−02	6.5294E−03	2.5323E−03	9.3430E−03	7.88
	HDE	9.8650E−04	1.8749E−03	1.1717E−03	1.0634E−03	2.7112E−04	7.92
	OBGBO	9.8593E−04	1.4188E−03	1.0429E−03	9.8602E−04	1.3590E−04	73.52
TDM	**OBEDO**	**9.8082E−04**	**1.1471E−03**	**9.9957E−04**	**9.8368E−04**	**5.1873E−05**	7.87
	EDO	1.2949E−03	3.8163E−02	1.0482E−02	4.0250E−03	1.4783E−02	**7.63**
	ADHHO	1.1473E−03	3.2969E−03	2.3185E−03	2.6095E−03	8.6274E−04	7.95
	OBMPA	9.8381E−04	3.1874E−02	4.4511E−03	1.5720E−03	9.6392E−03	7.95
	MGTO	9.8353E−04	1.3903E−03	1.0264E−03	9.8565E−04	1.2790E−04	8.41
	IAOA	1.5865E−03	3.6817E−02	1.0027E−02	4.0693E−03	1.3771E−02	7.94
	HDE	9.8732E−04	1.8554E−03	1.3337E−03	1.2150E−03	2.9969E−04	7.92
	OBGBO	9.8244E−04	1.3404E−03	1.0356E−03	9.8556E−04	1.1566E−04	73.91

Significant values are in [bold].

The IAE value resulting from using the OBEDO in the context of the SDM, DDM, and TDM for scenario 1 is notably below the threshold of 3.01E−03. This indicates a remarkably precise alignment between the predicted and observed values. Similarly, the RE value, which gauges the degree of dissimilarity between the predicted and actual values, demonstrates significant conformity as it remains under the stringent limit of 1.51E−01. A broader assessment of the performance metrics sheds light on the collective behaviour for scenario 1 under the purview of the OBEDO algorithm. The average RE and IAE values of SDM, DDM, and TDM are 4.74E−03, 4.50E−03, 4.51E−03, 8.28E−04, 8.19E−04, and 8.18E−04respectively, further reinforcing the efficacy of the optimization algorithm in achieving a robust convergence between the simulated and empirical outcomes. The comprehensive data showcased in Table 5 and Figs. 9, 10, and 11 corroborates the capability of the OBEDO to precisely extract the intricate characteristics of the PV cell within the specific framework. This affirmation of accuracy underscores the algorithm's competence in capturing the nuanced behaviour and performance attributes of the PV cell, thereby contributing to a deeper and more comprehensive understanding of its indefinite characteristics.

Table 8 summarizes the performance of various algorithms across three models: SDM, DDM, and TDM. The metrics assessed include Min, Max, Mean, Median, STD, and RT for each algorithm. Across all three models, the OBEDO algorithm consistently achieves low RMSE values, indicative of accurate predictions. Notably, it maintains a narrow RMSE range, resulting in stable and reliable outcomes. The EDO algorithm displays more variability in RMSE, particularly in the DDM and TDM models, where it reaches higher maximum RMSE values. This suggests that EDO performs well on average but may produce less precise predictions occasionally. ADHHO demonstrates generally consistent performance, maintaining moderate RMSE values and standard deviations. Its RMSE range remains relatively stable across the different models. OBMPA consistently produces moderate RMSE values with low standard deviations, indicating dependable performance. Its RMSE range is also relatively stable across models. MGTO consistently achieves low RMSE values with minimal variability, suggesting accurate and reliable predictions. Notably, its runtime is consistent across models. IAOA displays wider variability in RMSE, particularly in the SDM and DDM models, where it reaches higher maximum values. While its mean RMSE is reasonable, the variability suggests some potential for less accurate predictions. HDE generally maintains low and consistent RMSE values, with minor fluctuations. Its RMSE range remains stable across models, indicating dependable performance. OBGBO stands out with extremely low RMSE values, particularly in the SDM model. However, it comes with higher runtimes, especially in the TDM model. The OBEDO, OBGBO, and MGTO algorithms consistently exhibit accurate and reliable performance across the three models. OBMPA, EDO, ADHHO, IAOA, and HDE also show reasonable performance but with some variability. OBGBO demonstrates good accuracy, though at the cost of longer runtimes.

**Figure 12:**
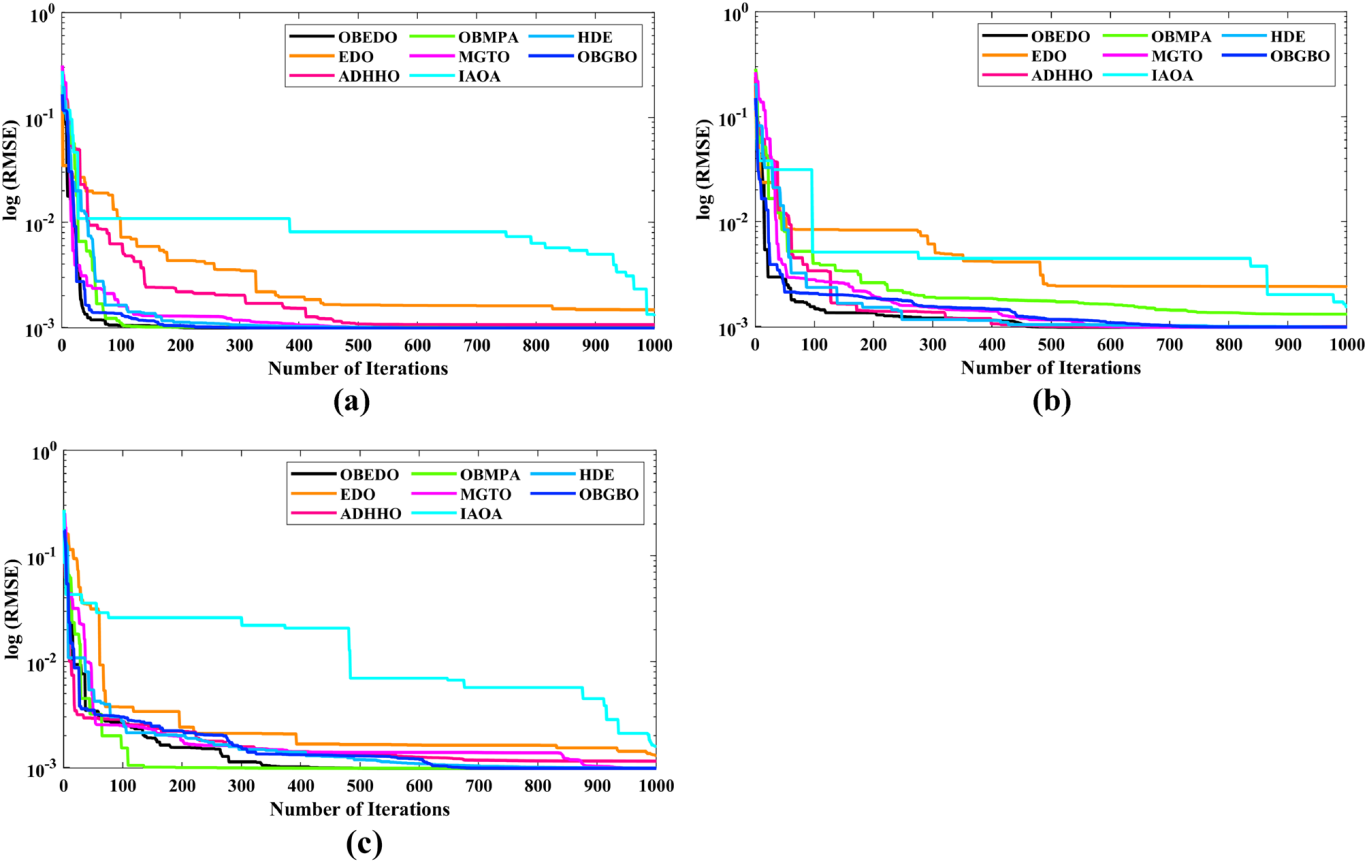
Convergence curves (Scenario 1); (**a**) SDM, (**b**) DDM, (**c**) TDM.

**Figure 13:**
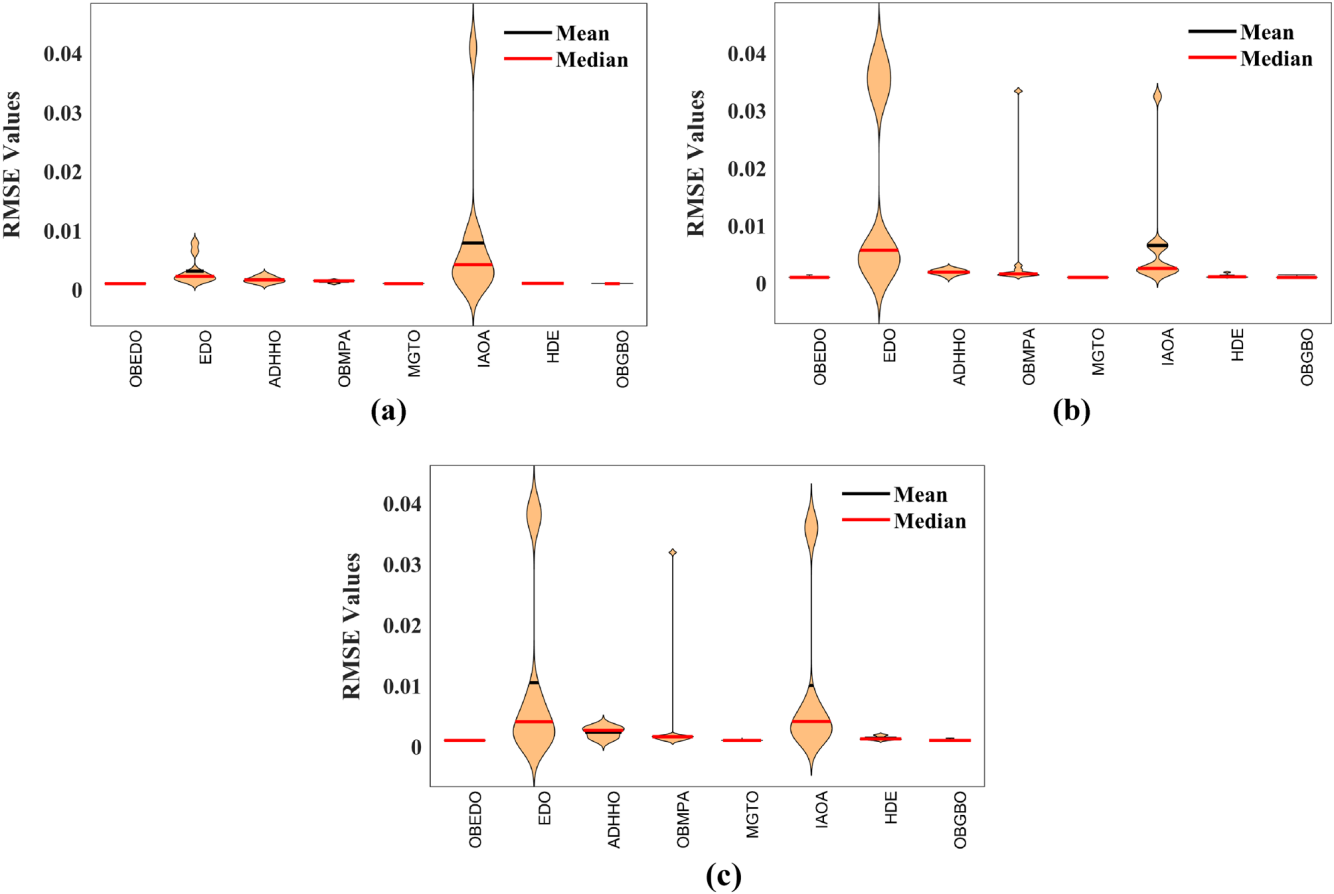
Violin plots (Scenario 1); (**a**) SDM, (**b**) DDM, (**c**) TDM.

The progression trajectories, characterizing the convergence behaviour, intricately unravel the dynamics of the SDM, DDM, and TDM for Scenario 1. These complex patterns of convergence are portrayed in the comprehensive Fig. 12. As the gaze fixes upon this graphical representation, the remarkably accelerated convergence of the OBEDO becomes noticeably apparent. This swift trajectory toward convergence, markedly outpacing its algorithmic counterparts, is a testament to the superior efficiency of the OBEDO. The unfolding narrative takes an artistic turn as we venture into Fig. 13, which, rather than opting for the conventional boxplot approach, ingeniously employs the visually captivating violin plot technique. Within this illustration, the distinct strategies outlined for the SDM, DDM, and TDM emerge as leading roles, each contributing its unique melodic note to the symphony of data visualization. In the symphony of violin plots, a striking revelation comes to the fore—OBEDO's reliability emerges as a true virtuoso. A harmonious refrain of low STD values resonates across all the photovoltaic models in Scenario 1.

This explicit stability display strengthens the OBEDO's position as an unmatched performer, standing high amidst its algorithmic companions. Interestingly, the plot's narrative takes a dramatic twist as a few algorithms stumble upon the snares of local minima, their quest for optimal outcomes thwarted by the intricate labyrinth of possibilities. Yet, amidst this unfolding drama emerges the OBEDO as the triumphant protagonist. It directs the complex optimization landscape, ascending to the peak of achievement with incomparable accuracy.

### Scenario 2—PVM752 GaAs PV Cell

**Figure 14:**
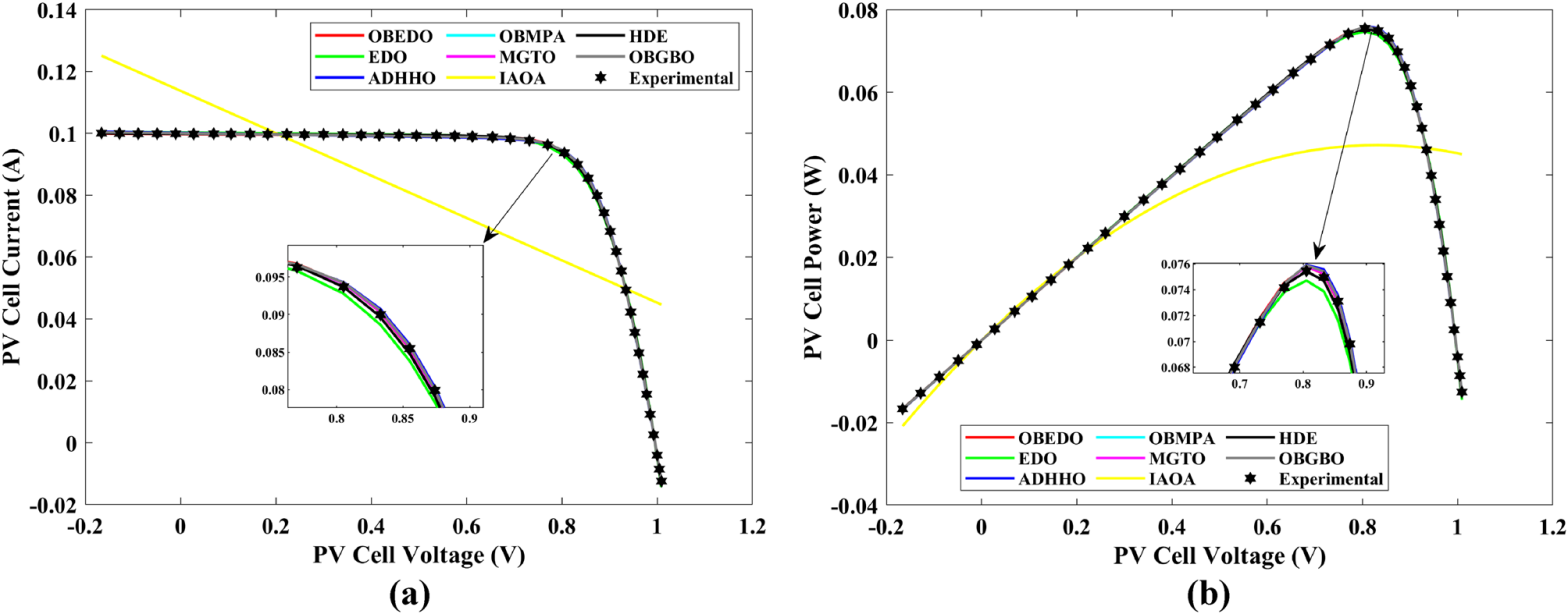
Characteristics curves obtained by algorithms for Scenario 2 (SDM); (**a**) I–V curves, (**b**) P–V curves.

**Figure 15:**
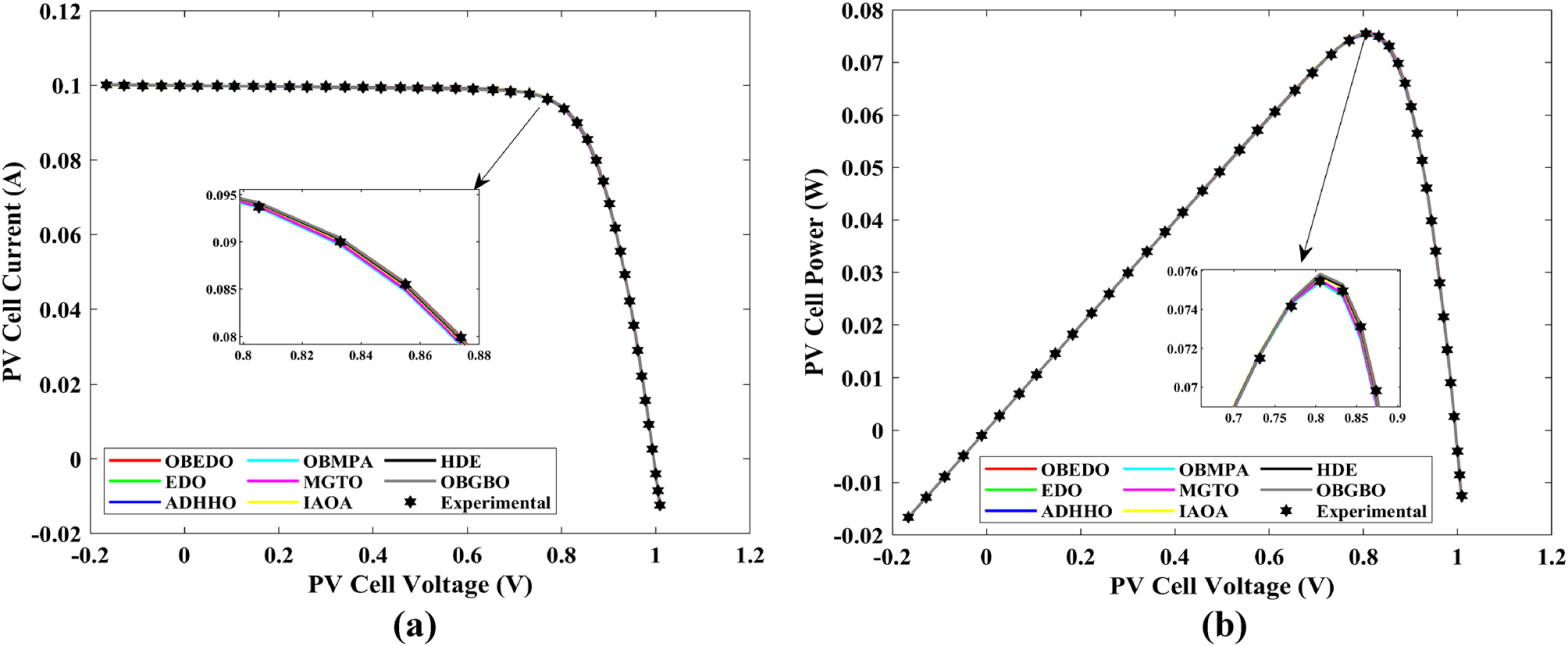
Characteristics curves obtained by algorithms for Scenario 2 (DDM); (**a**) I–V curves, (**b**) P–V curves.

**Figure 16:**
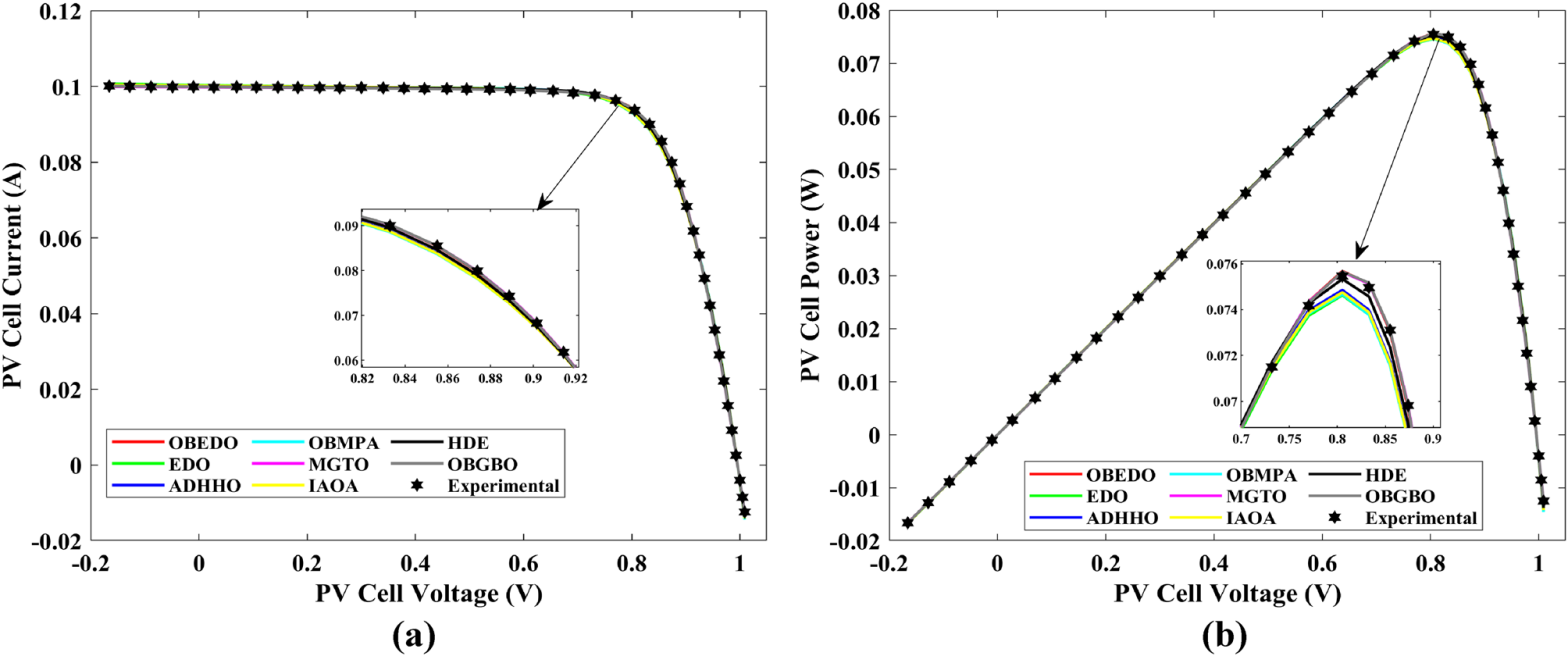
Characteristics curves obtained by algorithms for Scenario 2 (TDM); (**a**) I–V curves, (**b**) P–V curves.

**Figure 17:**
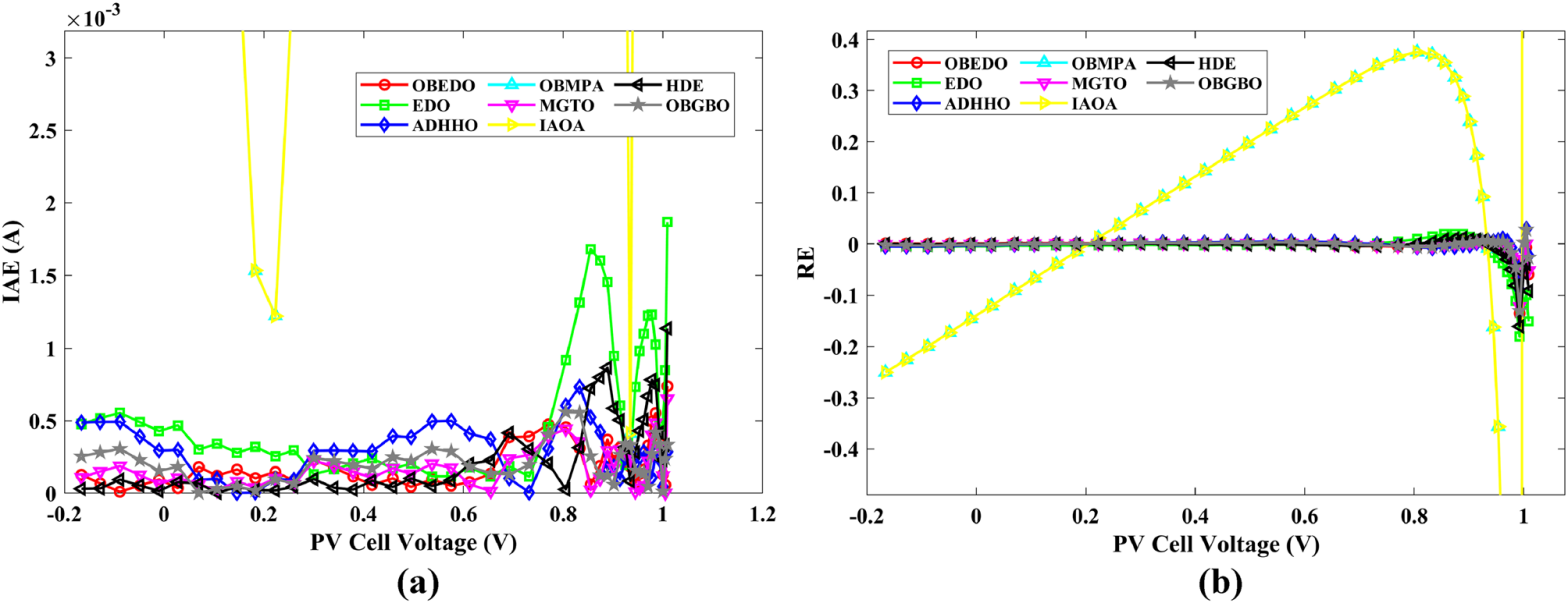
Error curves obtained by algorithms for Scenario 2 (SDM); (**a**) IAE, (**b**) RE.

**Figure 18:**
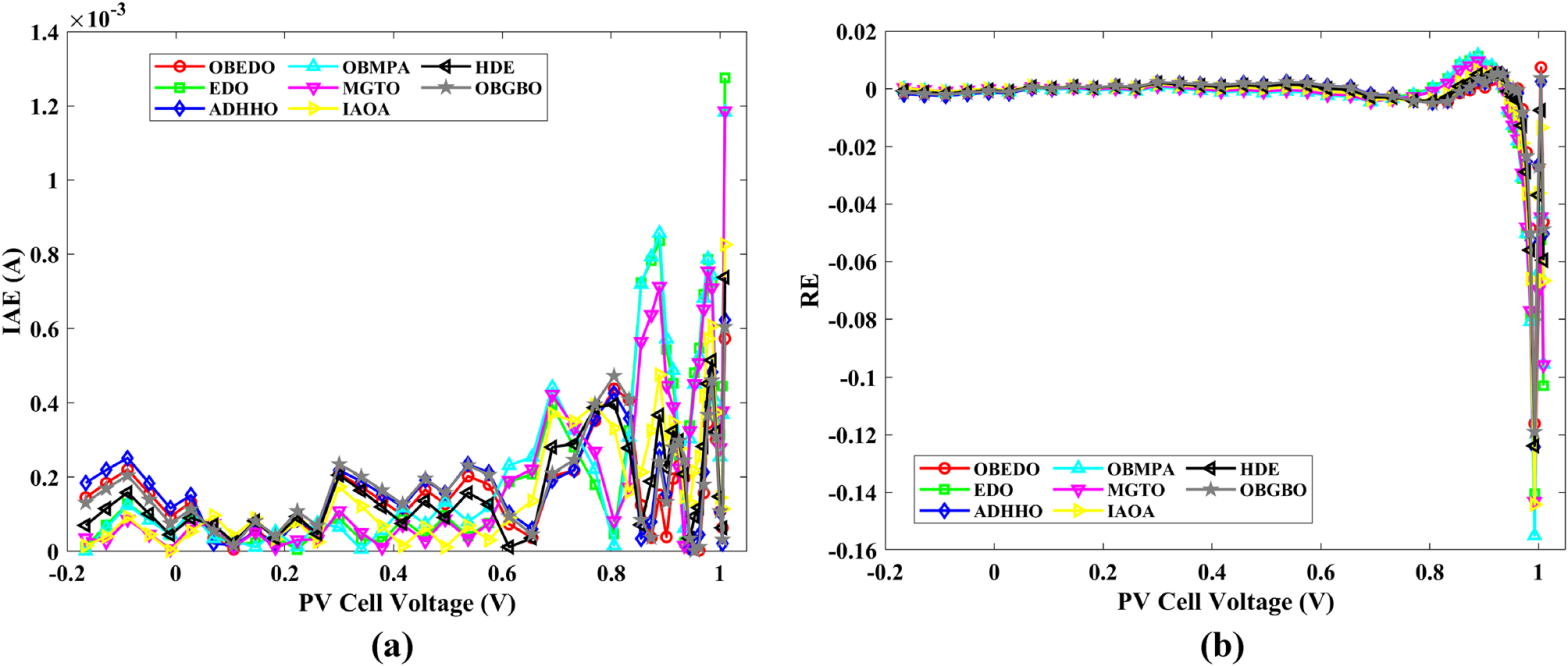
Error curves obtained by algorithms for Scenario 2 (DDM); (**a**) IAE, (**b**) RE.

**Figure 19:**
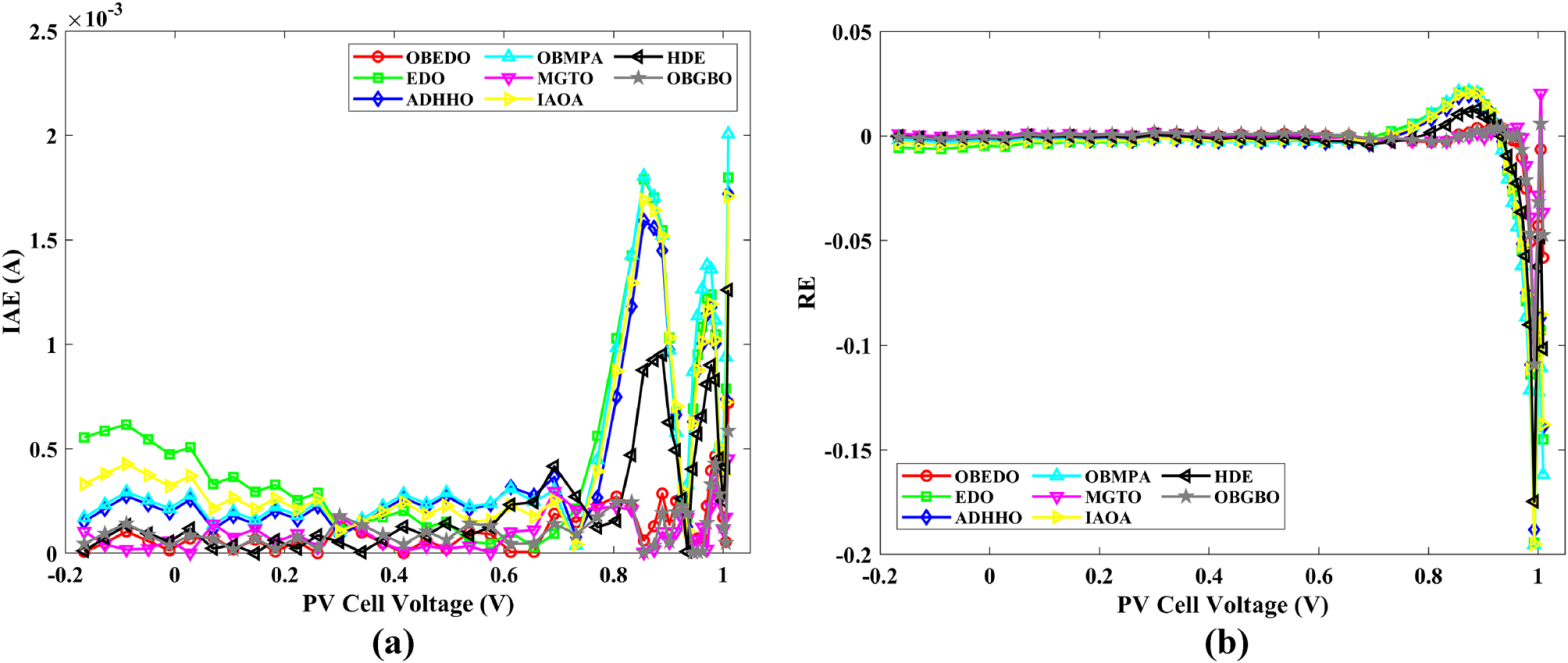
Error curves obtained by algorithms for Scenario 2 (TDM); (**a**) IAE, (**b**) RE.

This sub-section details the results obtained by the proposed algorithm and other algorithms for scenario 2, i.e., SDM, DDM, and TDM of the PVM752 GaAs PV cell. The bounds for all the PV models are provided in Table 2. Tables 9, 10, and 11 display a comprehensive overview of the five, seven, and nine parameters estimated through the SDM, DDM, and TDM employment pertaining to the PVM752 GaAs PV cell. A graphical representation of the I–V and P–V characteristic curves pertinent to the SDM, DDM, and TDM of the PVM752 GaAs PV cell obtained by all algorithms has been expounded in Figs. 14, 15, and 16. The quantitative values summarizing the IAE and the RE must be meticulously documented in Table 12. To visually portray the paramount significance of the RE and IAE values ascertained through the instrumentality of the OBEDO and other algorithms, this study has deviously created error graphs as showcased in Figs. 17, 18, and 19. A cursory examination of Figs. 14, 15, 16, 17, 18, and 19 unfolds a noticeably perfect alignment between the estimated and experimental curves. This alignment strengthens the inference that the curve fitting has yielded a worthy equivalence. To offer a comprehensive assessment, meticulous cataloguing of statistical indicators has been meticulously undertaken. These values have been carefully logged within Table 13 to facilitate in-depth data comprehension.

**Table 9:** Parameters estimated for Scenario 2 (SDM).

Algorithms	${I}_{ph}$(A)	${I}_{sd}$(A)	${R}_{se}$(Ω)	${R}_{sh}$(Ω)	$n$	RMSE
OBEDO	0.10016	1.85E−12	0.6777	519.07	1.5691	2.4818E−04
EDO	0.10039	3.75E−10	0.5173	623.65	1.9985	7.5916E−04
ADHHO	0.10034	5.91E−13	0.6982	394.73	1.5000	3.2683E−04
OBMPA	0.11376	0.00E+00	0.0000	14.59	1.0000	2.5400E−02
MGTO	**0.10005**	**4.73E−12**	**0.6553**	**637.38**	**1.6309**	**2.2951E−04**
IAOA	0.11377	0.00E+00	0.0000	14.59	1.1265	2.5400E−02
HDE	0.09996	4.59E−11	0.5954	998.48	1.8029	3.9734E−04
OBGBO	0.09986	8.34E−12	0.6428	925.40	1.6706	2.5694E−04

Significant values are in [bold].

**Table 10:** Parameters estimated for Scenario 2 (DDM).

Algorithms	${I}_{ph}$(A)	${I}_{sd1}$(A)	${R}_{se}$(Ω)	${R}_{sh}$(Ω)	${n}_{1}$	${I}_{sd2}$(A)	${n}_{2}$	RMSE
OBEDO	**0.10009**	**1.89E−14**	**0.6786**	**618.21**	**1.3561**	**2.43E−11**	**1.8068**	**2.0777E−04**
EDO	0.10000	4.77E−11	0.5922	933.01	1.8062	0.00E+00	1.0823	4.0637E−04
ADHHO	0.10011	0.00E+00	0.6579	580.01	1.5289	4.13E−12	1.6217	2.2965E−04
OBMPA	0.09999	4.85E−11	0.5934	999.51	1.8076	0.00E+00	1.0061	4.0415E−04
MGTO	0.09996	3.50E−11	0.6015	984.06	1.7806	0.00E+00	1.0000	3.6952E−04
IAOA	0.09996	0.00E+00	0.6328	833.80	1.4533	1.19E−11	1.6965	2.7060E−04
HDE	0.10002	6.65E−12	0.6471	689.52	1.6546	0.00E+00	1.8345	2.3856E−04
OBGBO	0.10007	0.00E+00	0.6605	607.97	1.9995	3.78E−12	1.6157	2.2780E−04

Significant values are in [bold].

**Table 11:** Parameters estimated for Scenario 2 (TDM).

Algorithms	${I}_{ph}$(A)	${I}_{sd1}$(A)	${R}_{se}$(Ω)	${R}_{sh}$(Ω)	${n}_{1}$	${I}_{sd2}$(A)	${n}_{2}$	${I}_{sd3}$(A)	${n}_{3}$	RMSE
OBEDO	**0.09998**	**1.81E−12**	**0.6632**	**874.65**	**1.5696**	**6.65E−18**	**1.2262**	**2.99E−07**	**4.1442**	1.9516E−04
EDO	0.10045	0.00E+00	0.5170	555.19	1.3771	0.00E+00	1.1643	3.79E−10	2.0000	7.8463E−04
ADHHO	0.10014	2.50E−16	0.5225	1000.00	1.4996	0.00E+00	1.5000	3.81E−10	2.0000	6.9917E−04
OBMPA	0.10015	0.00E+00	0.4940	999.40	1.0055	0.00E+00	1.0151	7.14E−10	2.0667	8.0321E−04
MGTO	0.09990	0.00E+00	0.7314	998.41	1.0000	0.00E+00	1.0596	1.86E−10	2.0000	**1.5093E−04**
IAOA	0.10028	0.00E+00	0.5212	743.48	1.1178	3.80E−10	1.9997	0.00E+00	4.4637	7.3145E−04
HDE	0.10000	0.00E+00	0.5804	1000.00	1.9303	7.19E−11	1.8415	3.32E−11	4.5533	4.5492E−04
OBGBO	0.10002	2.42E−13	0.6828	745.81	1.4562	0.00E+00	1.0011	8.28E−09	2.7386	1.7128E−04

Significant values are in [bold].

**Table 12:** RE and IAE values attained by OBEDO for Scenario 2.

${V}_{ex}$	${I}_{ex}$	SDM	DDM	TDM	SDM	DDM	TDM	SDM	DDM	TDM
		${I}_{es}$(A)			$RE$			$IAE$(A)		
− 0.1659	0.1001	0.1000	0.1002	0.1001	1.26E−03	− 1.45E−03	7.37E−05	1.26E−04	1.45E−04	7.38E−06
− 0.1281	0.1000	0.0999	0.1002	0.1000	6.67E−04	− 1.84E−03	− 4.94E−04	6.67E−05	1.84E−04	4.94E−05
− 0.0888	0.0999	0.0999	0.1001	0.1000	9.15E−05	− 2.21E−03	− 1.05E−03	9.14E−06	2.20E−04	1.05E−04
− 0.0490	0.0999	0.0998	0.1001	0.1000	5.22E−04	− 1.56E−03	− 5.89E−04	5.22E−05	1.56E−04	5.89E−05
− 0.0102	0.0999	0.0998	0.1000	0.0999	9.42E−04	− 9.34E−04	− 1.44E−04	9.41E−05	9.34E−05	1.44E−05
0.0275	0.0998	0.0998	0.0999	0.0999	3.48E−04	− 1.33E−03	− 7.13E−04	3.47E−05	1.32E−04	7.11E−05
0.0695	0.0999	0.0997	0.0999	0.0998	1.80E−03	3.56E−04	7.74E−04	1.80E−04	3.56E−05	7.73E−05
0.1061	0.0998	0.0997	0.0998	0.0998	1.20E−03	− 5.35E−05	1.96E−04	1.20E−04	5.34E−06	1.95E−05
0.1460	0.0998	0.0996	0.0997	0.0997	1.63E−03	5.93E−04	6.60E−04	1.63E−04	5.92E−05	6.58E−05
0.1828	0.0997	0.0996	0.0997	0.0997	1.03E−03	1.87E−04	8.76E−05	1.03E−04	1.86E−05	8.73E−06
0.2230	0.0997	0.0996	0.0996	0.0996	1.46E−03	8.39E−04	5.63E−04	1.46E−04	8.37E−05	5.61E−05
0.2600	0.0996	0.0995	0.0996	0.0996	8.62E−04	4.36E−04	2.26E−06	8.59E−05	4.34E−05	2.25E−07
0.3001	0.0997	0.0995	0.0995	0.0996	2.30E−03	2.09E−03	1.50E−03	2.29E−04	2.08E−04	1.49E−04
0.3406	0.0996	0.0994	0.0994	0.0995	1.74E−03	1.75E−03	1.00E−03	1.73E−04	1.74E−04	9.97E−05
0.3789	0.0995	0.0994	0.0994	0.0995	1.15E−03	1.37E−03	4.96E−04	1.15E−04	1.36E−04	4.93E−05
0.4168	0.0994	0.0993	0.0993	0.0994	5.61E−04	9.82E−04	1.12E−05	5.58E−05	9.76E−05	1.11E−06
0.4583	0.0994	0.0993	0.0992	0.0993	1.02E−03	1.67E−03	6.27E−04	1.02E−04	1.66E−04	6.24E−05
0.4949	0.0993	0.0993	0.0992	0.0993	4.35E−04	1.29E−03	2.23E−04	4.32E−05	1.28E−04	2.22E−05
0.5370	0.0993	0.0992	0.0991	0.0992	9.57E−04	2.04E−03	1.03E−03	9.50E−05	2.03E−04	1.02E−04
0.5753	0.0992	0.0991	0.0990	0.0991	5.11E−04	1.81E−03	9.27E−04	5.07E−05	1.80E−04	9.20E−05
0.6123	0.0990	0.0991	0.0989	0.0990	− 7.70E−04	7.41E−04	6.97E−05	7.63E−05	7.34E−05	6.90E−06
0.6546	0.0988	0.0989	0.0988	0.0988	− 1.36E−03	3.68E−04	6.88E−05	1.34E−04	3.64E−05	6.80E−06
0.6918	0.0983	0.0987	0.0985	0.0985	− 3.92E−03	− 2.06E−03	− 1.93E−03	3.85E−04	2.03E−04	1.90E−04
0.7318	0.0977	0.0981	0.0979	0.0978	− 4.00E−03	− 2.22E−03	− 1.53E−03	3.91E−04	2.17E−04	1.49E−04
0.7702	0.0963	0.0968	0.0967	0.0965	− 4.92E−03	− 3.65E−03	− 2.38E−03	4.74E−04	3.52E−04	2.29E−04
0.8053	0.0937	0.0942	0.0941	0.0940	− 4.86E−03	− 4.68E−03	− 2.90E−03	4.55E−04	4.38E−04	2.72E−04
0.8329	0.0900	0.0903	0.0904	0.0902	− 3.44E−03	− 4.55E−03	− 2.46E−03	3.10E−04	4.09E−04	2.22E−04
0.8550	0.0855	0.0854	0.0856	0.0854	7.02E−04	− 1.46E−03	7.18E−04	6.00E−05	1.24E−04	6.14E−05
0.8738	0.0799	0.0797	0.0799	0.0798	2.36E−03	− 4.57E−04	1.62E−03	1.88E−04	3.65E−05	1.30E−04
0.8887	0.0743	0.0739	0.0741	0.0740	4.97E−03	2.05E−03	3.85E−03	3.69E−04	1.52E−04	2.86E−04
0.9016	0.0683	0.0681	0.0683	0.0682	3.28E−03	5.66E−04	2.02E−03	2.24E−04	3.87E−05	1.38E−04
0.9141	0.0618	0.0615	0.0616	0.0615	5.11E−03	3.16E−03	4.05E−03	3.16E−04	1.96E−04	2.50E−04
0.9248	0.0555	0.0552	0.0553	0.0553	5.09E−03	4.17E−03	4.45E−03	2.82E−04	2.32E−04	2.47E−04
0.9344	0.0493	0.0491	0.0491	0.0491	3.71E−03	4.03E−03	3.66E−03	1.83E−04	1.98E−04	1.80E−04
0.9445	0.0422	0.0423	0.0422	0.0422	− 1.62E−03	1.83E−04	− 8.86E−04	6.83E−05	7.71E−06	3.74E−05
0.9533	0.0357	0.0358	0.0357	0.0358	− 3.76E−03	− 1.85E−04	− 2.00E−03	1.34E−04	6.59E−06	7.15E−05
0.9618	0.0291	0.0293	0.0291	0.0292	− 5.58E−03	8.80E−05	− 2.51E−03	1.62E−04	2.56E−06	7.30E−05
0.9702	0.0222	0.0225	0.0224	0.0224	− 1.48E−02	− 7.09E−03	− 1.02E−02	3.28E−04	1.57E−04	2.27E−04
0.9778	0.0157	0.0162	0.0160	0.0161	− 3.15E−02	− 2.20E−02	− 2.52E−02	4.95E−04	3.46E−04	3.96E−04
0.9852	0.0092	0.0098	0.0096	0.0097	− 6.00E−02	− 4.85E−02	− 5.06E−02	5.52E−04	4.46E−04	4.66E−04
0.9926	0.0026	0.0030	0.0029	0.0029	− 1.35E−01	− 1.16E−01	− 1.09E−01	3.51E−04	3.02E−04	2.83E−04
0.9999	− 0.0040	− 0.0041	− 0.0041	− 0.0042	− 3.21E−02	− 2.67E−02	− 4.29E−02	1.29E−04	1.07E−04	1.71E−04
1.0046	− 0.0085	− 0.0086	− 0.0084	− 0.0086	− 6.70E−03	7.47E−03	− 6.44E−03	5.69E−05	6.35E−05	5.47E−05
1.0089	− 0.0124	− 0.0131	− 0.0130	− 0.0131	− 5.95E−02	− 4.62E−02	− 5.82E−02	7.38E−04	5.73E−04	7.21E−04
Mean values					− 7.46E−03	− 5.84E−03	− 6.67E−03	2.02E−04	1.63E−04	1.36E−04

**Table 13:** Performance Comparison for Scenario 2.

Model	Algorithms	$Min$	$Max$	$Mean$	$Median$	$STD$	$RT$
SDM	**OBEDO**	2.4818E−04	**2.2921E−02**	**2.5343E−03**	**2.6537E−04**	7.1631E−03	7.12
	EDO	7.5916E−04	2.5401E−02	1.9005E−02	2.5400E−02	1.0602E−02	**6.79**
	ADHHO	3.2683E−04	2.5400E−02	9.7083E−03	5.8528E−04	1.2161E−02	7.44
	OBMPA	2.5400E−02	2.5400E−02	2.5400E−02	2.5400E−02	2.0031E−18	7.44
	MGTO	**2.2951E−04**	2.4865E−02	5.6162E−03	5.7538E−04	8.4651E−03	7.96
	IAOA	2.5400E−02	2.5402E−02	2.5401E−02	2.5401E−02	**9.8394E−07**	7.18
	HDE	3.9734E−04	2.5400E−02	1.5414E−02	2.5400E−02	1.2891E−02	8.98
	OBGBO	2.5694E−04	2.5400E−02	7.8244E−03	3.0311E−04	1.2128E−02	67.83
DDM	**OBEDO**	**2.0777E−04**	**2.5400E−02**	**2.7714E−03**	**2.4537E−04**	7.9509E−03	6.79
	EDO	4.0637E−04	2.5400E−02	1.7291E−02	2.5400E−02	1.0696E−02	**6.65**
	ADHHO	2.2965E−04	2.5400E−02	4.8199E−03	5.1218E−04	8.5350E−03	11.61
	OBMPA	4.0415E−04	2.5400E−02	2.2900E−02	2.5400E−02	**7.9042E−03**	6.90
	MGTO	3.6952E−04	2.5400E−02	1.4631E−02	1.2183E−02	9.8463E−03	7.07
	IAOA	2.7060E−04	2.5405E−02	1.3110E−02	1.3450E−02	1.2960E−02	6.99
	HDE	2.3856E−04	2.5400E−02	2.2883E−02	2.5400E−02	7.9566E−03	6.85
	OBGBO	2.2780E−04	2.5400E−02	3.2032E−03	2.7817E−04	7.9050E−03	61.42
TDM	**OBEDO**	1.9516E−04	1.3192E−03	7.2246E−04	7.0912E−04	4.4160E−04	6.64
	EDO	7.8463E−04	2.5400E−02	9.0979E−03	2.4189E−03	1.1263E−02	**6.31**
	ADHHO	6.9917E−04	2.1651E−03	1.7327E−03	2.0094E−03	5.6225E−04	6.70
	OBMPA	8.0321E−04	2.2026E−03	1.5454E−03	1.5933E−03	3.4303E−04	6.77
	MGTO	**1.5093E−04**	**6.9936E−04**	**5.3367E−04**	**6.9936E−04**	**2.3021E−04**	6.89
	IAOA	7.3145E−04	2.5691E−03	1.6820E−03	2.1165E−03	7.8815E−04	6.88
	HDE	4.5492E−04	1.5908E−03	1.3879E−03	1.4810E−03	3.3089E−04	7.82
	OBGBO	1.7128E−04	2.2026E−03	7.4740E−04	6.9947E−04	5.6100E−04	63.99

Significant values are in [bold].

The IAE value resulting from using the OBEDO in the context of the SDM, DDM, and TDM for scenario 2 is notably below the threshold of 9.01E−04. This indicates a remarkably precise alignment between the predicted and observed values. Similarly, the RE value, which gauges the degree of dissimilarity between the predicted and actual values, demonstrates significant conformity as it remains under the stringent limit of 1.51E−02. The average RE and IAE values of SDM, DDM, and TDM are − 7.46E−03, − 5.84E−03, − 6.67E−03, 2.02E−04, 1.63E−04, and 1.36E−04, respectively, further reinforcing the efficacy of the optimization algorithm in achieving a robust convergence between the simulated and empirical outcomes. The comprehensive data showcased in Table 12 and Figs. 17, 18, and 19 corroborates the capability of the OBEDO to precisely extract the intricate characteristics of the PV cell within the specific framework. This affirmation of accuracy underscores the algorithm's competence in capturing the nuanced behaviour and performance attributes of the PV cell, thereby contributing to a deeper and more comprehensive understanding of its indefinite characteristics.

Table 13 summarizes the performance of various algorithms across three models: SDM, DDM, and TDM. Across all the PV models, the algorithms exhibit notable variations in their capabilities. The OBEDO algorithm consistently demonstrates commendable accuracy, as reflected by its frequently low IAE values, ensuring precise predictions. Contrasting this, the EDO algorithm presents a broader spectrum of RMSE values, potentially indicating instances where predictions may be less precise. The ADHHO algorithm maintains consistent and moderate RMSE values, reflecting stable performance across the PV models. Similarly, the OBMPA algorithm displays uniform RMSE values, suggesting reliable outcomes across different scenarios. The MGTO algorithm consistently produces low RMSE values with minimal variability, indicating its potential for reliable predictions. Meanwhile, the IAOA algorithm exhibits more variability in RMSE, especially in the SDM and DDM models. Remarkably, the HDE algorithm consistently maintains low and consistent IAE values across all PV models. The OBGBO algorithm stands out for its exceptional accuracy, albeit with longer runtimes. In summary, the algorithms' performance reveals a diverse landscape of accuracy, convergence, and efficiency. While some algorithms shine in specific models, the OBEDO, MGTO, and HDE algorithms consistently exhibit robust and dependable performance across various photovoltaic models. The findings highlight the crucial interplay between accuracy and efficiency, underscoring the necessity for a comprehensive evaluation of optimization algorithms in photovoltaic modelling. The IAOA and OBMPA failed to trace the I–V and curves for the SDM because both get trapped by the local optima.

**Figure 20:**
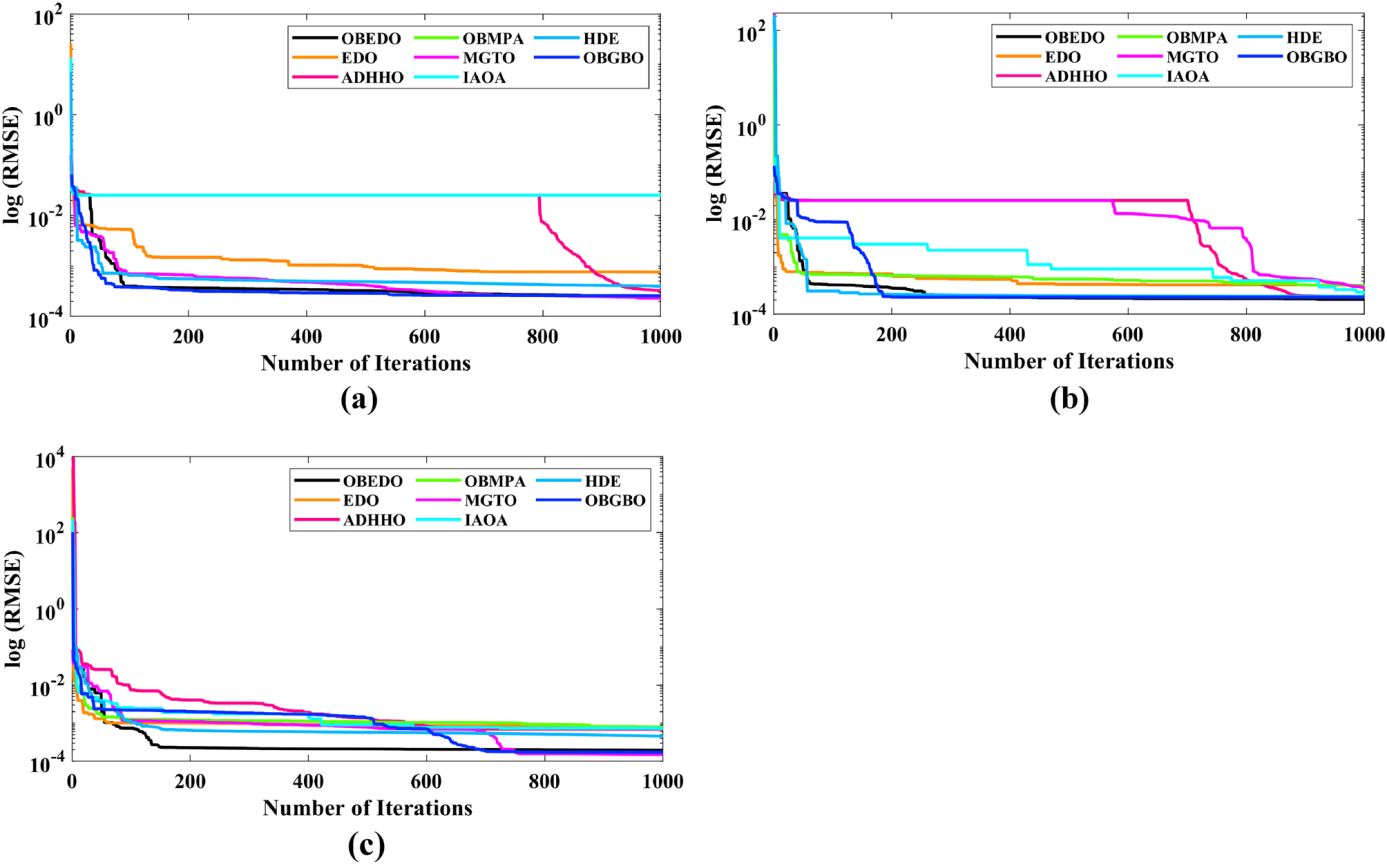
Convergence curves (Scenario 2); (**a**) SDM, (**b**) DDM, (**c**) TDM.

**Figure 21:**
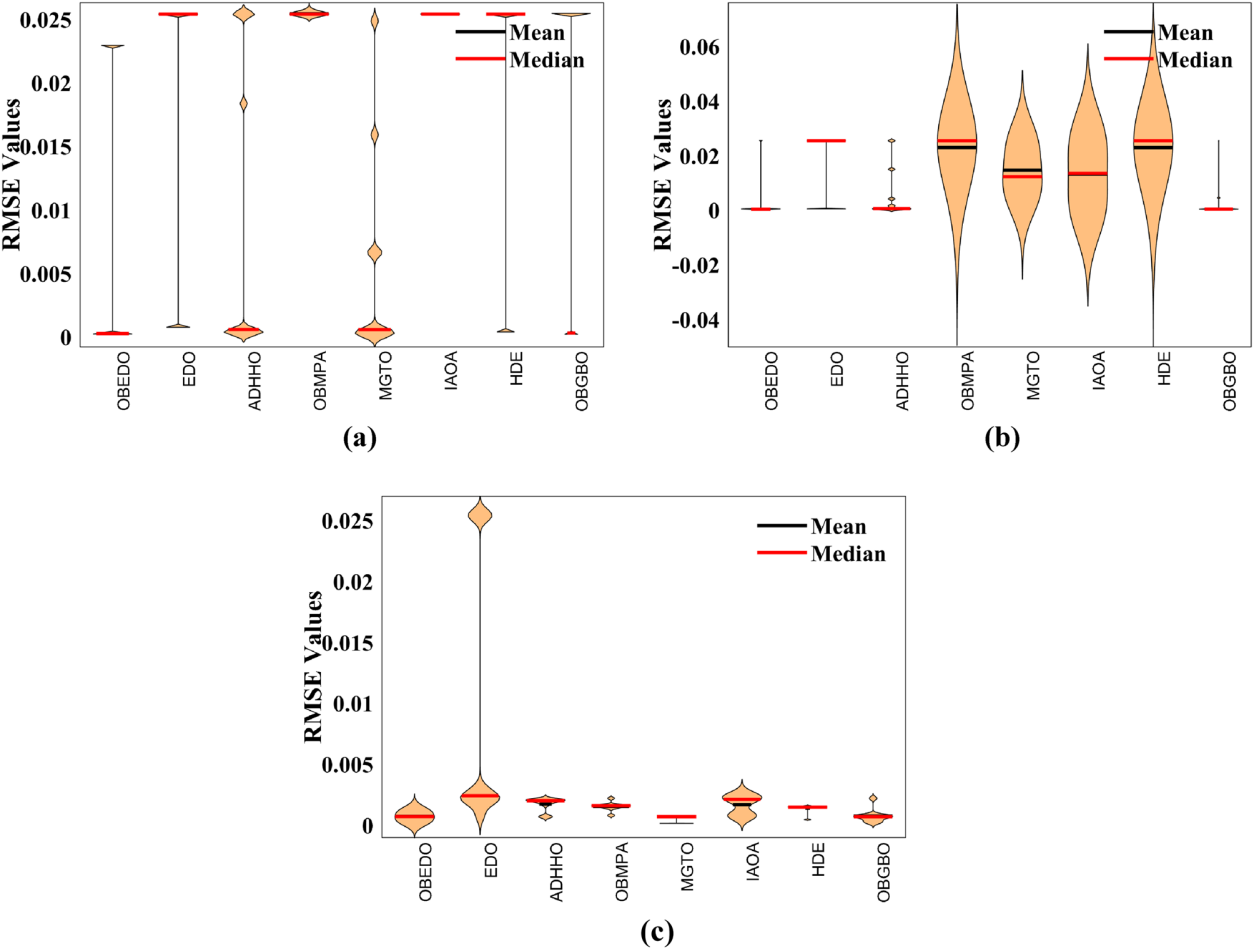
Violin plots (Scenario 2); (**a**) SDM, (**b**) DDM, (**c**) TDM.

The convergence dynamics of the SDM, DDM, and TDM for Scenario 2 are meticulously unveiled through intricate progression trajectories, portrayed vividly in Fig. 20. Upon inspecting this visual depiction, the rapid and impressive convergence of OBEDO becomes clearly evident. This accelerated trajectory surpasses its algorithmic counterparts, underscoring OBEDO's exceptional efficiency. The narrative takes an artistic turn with Fig. 21, which employs the visually captivating violin plot.

The strategies defined for SDM, DDM, and TDM play leading roles, each contributing a distinct note to the symphony of data visualization. Within the realm of the symphony of violin plots, a remarkable revelation unfolds—OBEDO emerges as a virtuoso of reliability. The attentive observer discerns the delicate undercurrents of STD values. The low STD values resonate across all photovoltaic models in Scenario 2. This steadfast stability further solidifies OBEDO's stature as an unmatched performer, towering among its algorithmic peers.

### Scenario 3—Photowatt PWP-201 PV module

**Figure 22:**
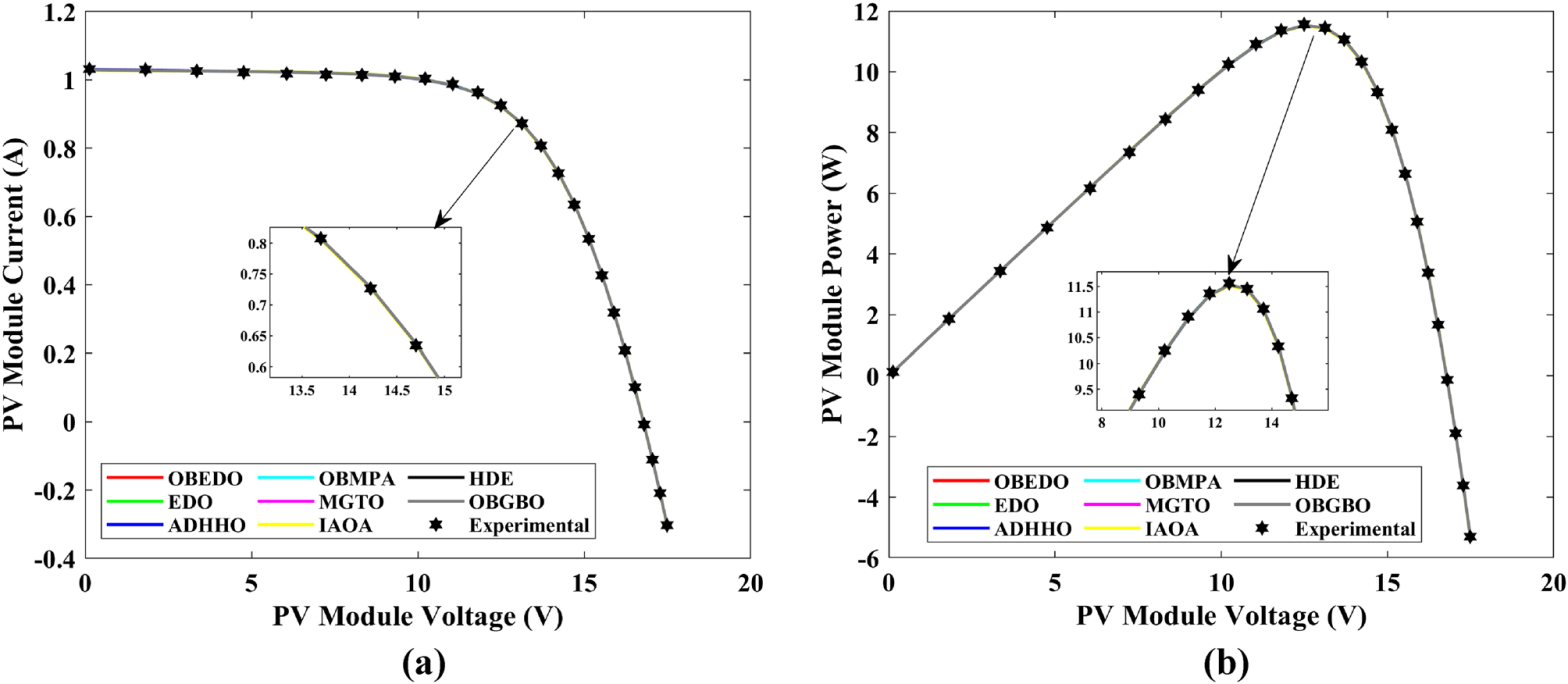
Characteristics curves obtained by algorithms for Scenario 3 (SDM); (**a**) I–V curves, (**b**) P–V curves.

**Figure 23:**
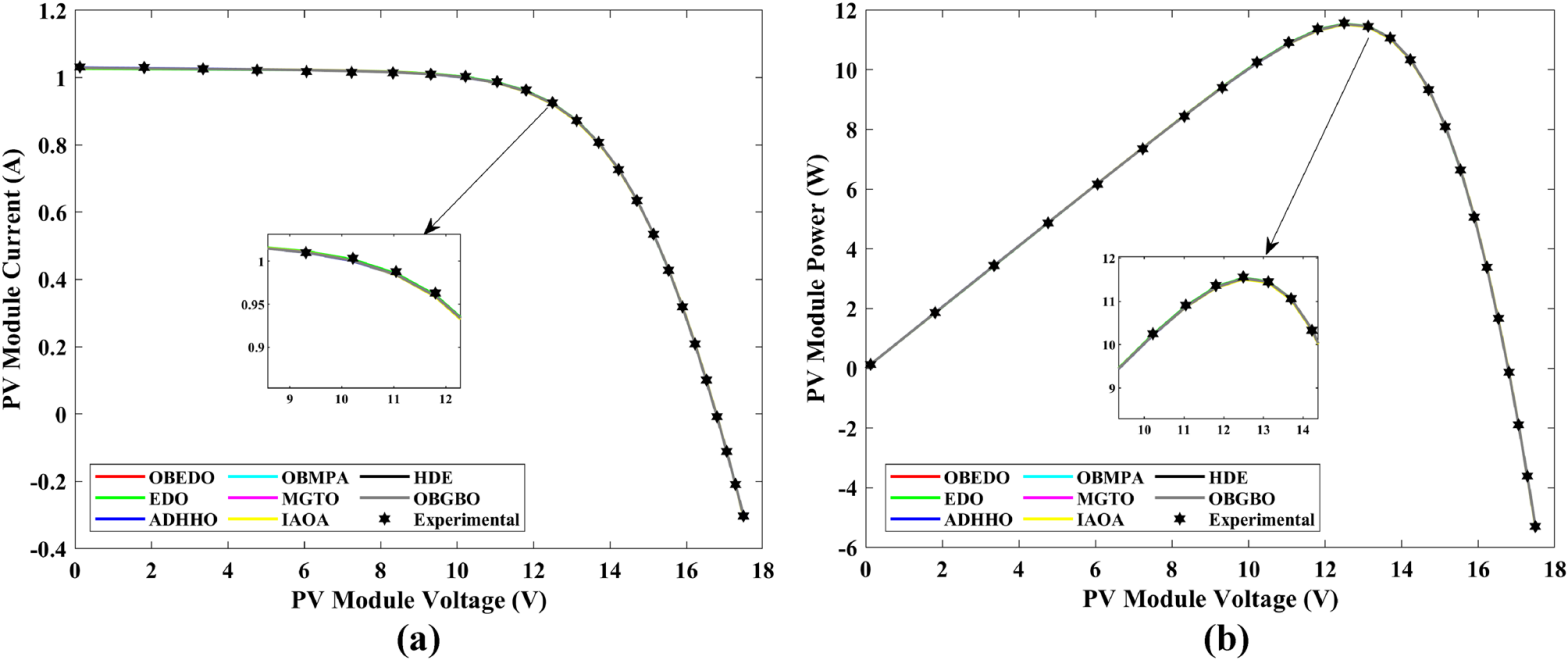
Characteristics curves obtained by algorithms for Scenario 3 (DDM); (**a**) I–V curves, (**b**) P–V curves.

**Figure 24:**
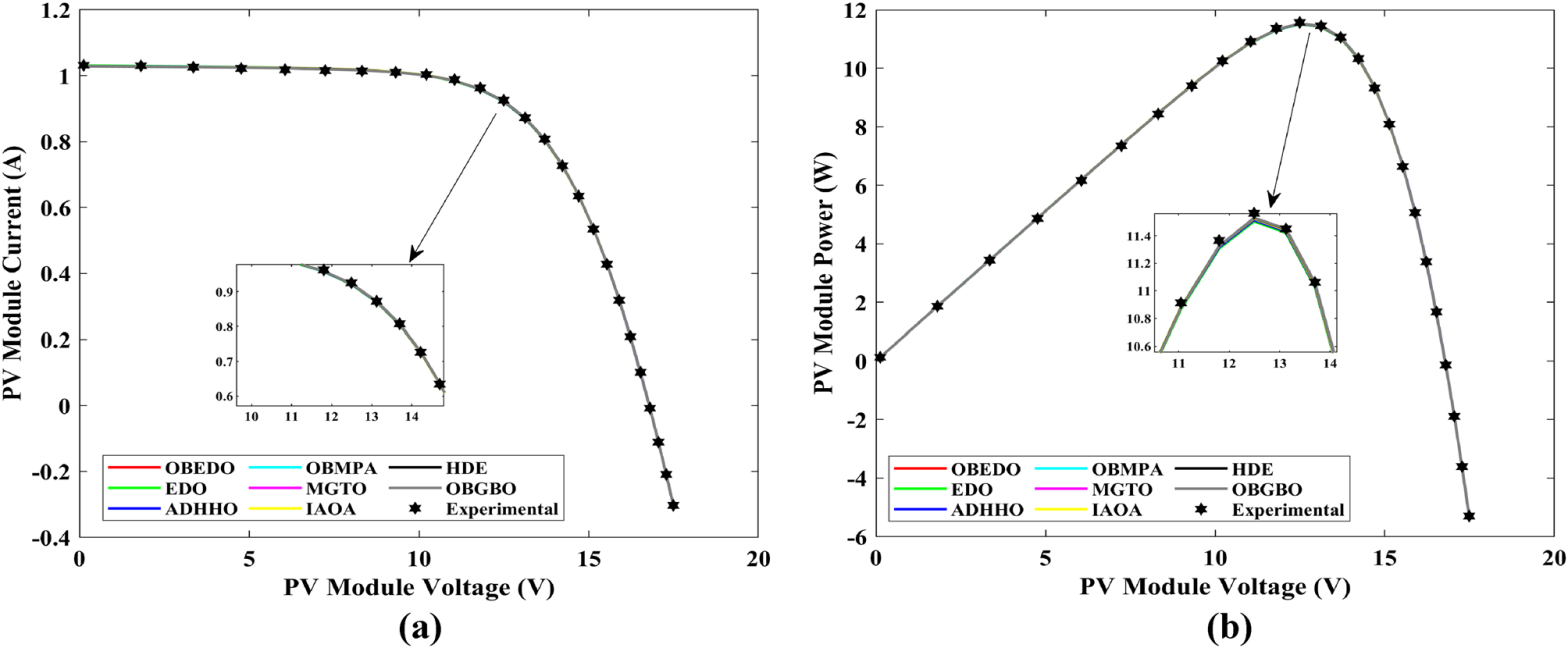
Characteristics curves obtained by algorithms for Scenario 3 (TDM); (**a**) I–V curves, (**b**) P–V curves.

**Figure 25:**
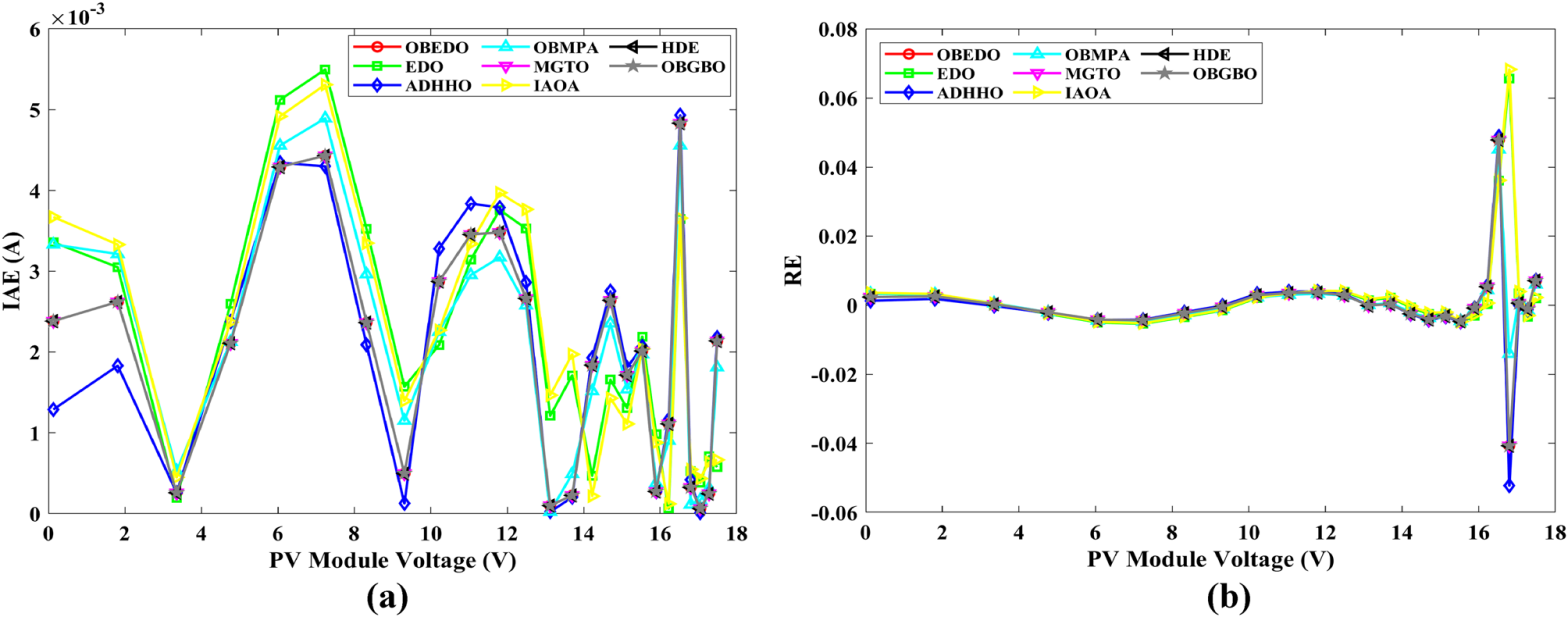
Error curves obtained by algorithms for Scenario 3 (SDM); (**a**) IAE, (**b**) RE.

**Figure 26:**
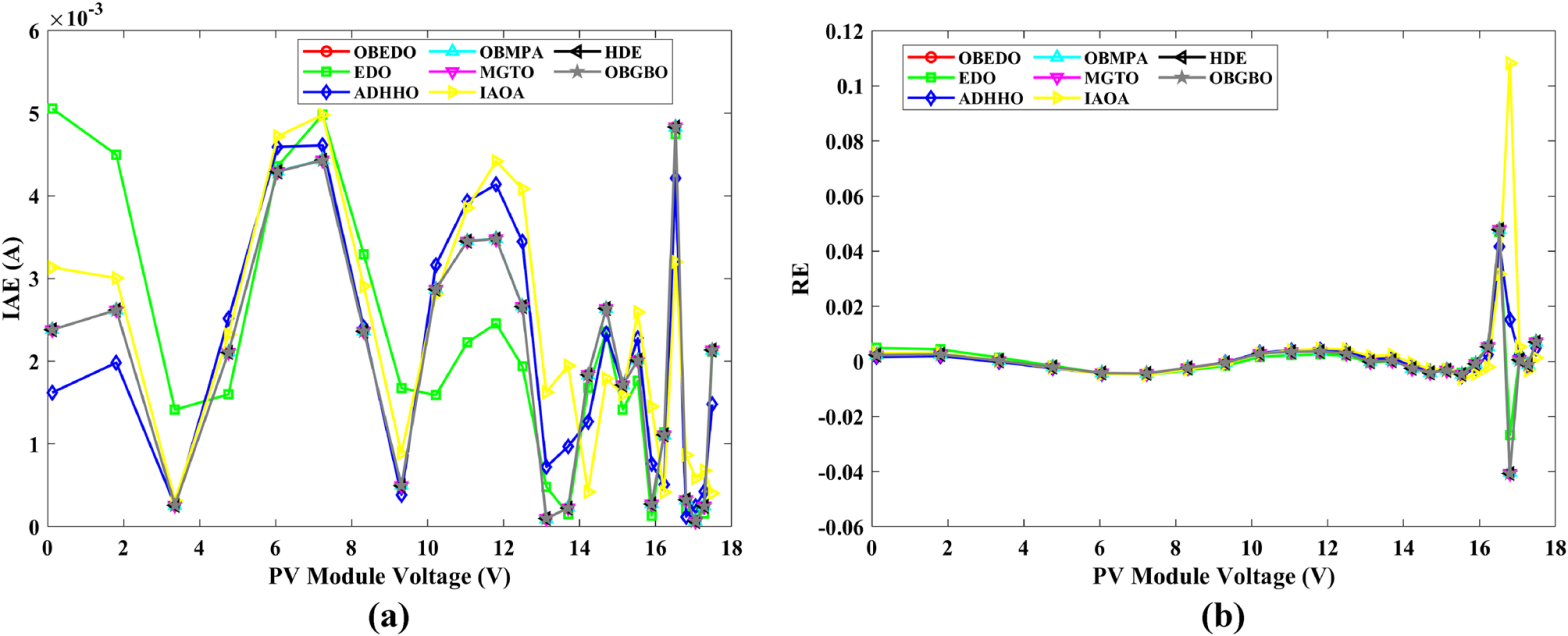
Error curves obtained by algorithms for Scenario 3 (DDM); (**a**) IAE, (**b**) RE.

**Figure 27:**
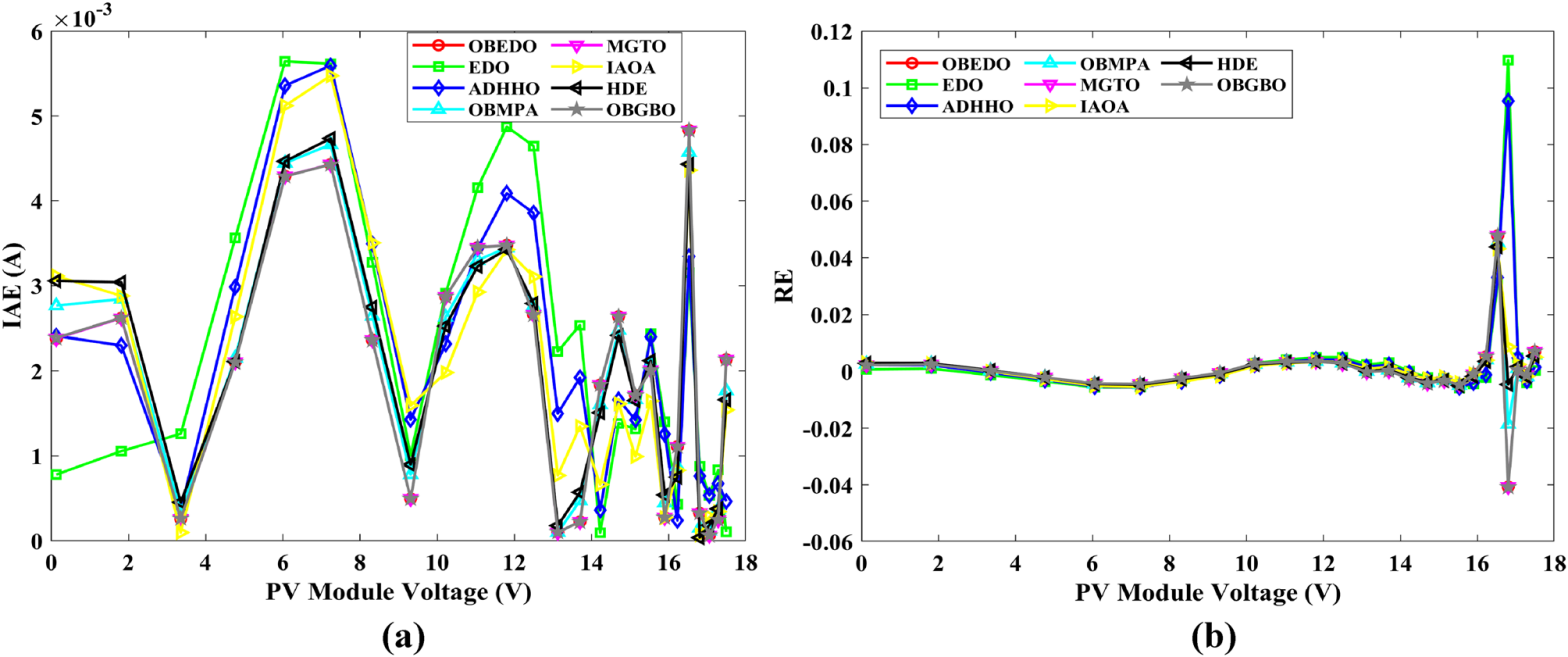
Error curves obtained by algorithms for Scenario 3 (TDM); (**a**) IAE, (**b**) RE.

This sub-section details the results obtained by the proposed algorithm and other algorithms for scenario 3, i.e., SDM, DDM, and TDM of the Photowatt PWP-201 PV module. The bounds for all the PV models are provided in Table 2. Tables 14, 15, and 16 display a comprehensive overview of the five, seven, and nine parameters estimated through the SDM, DDM, and TDM employment pertaining to the Photowatt PWP-201 PV module. A graphical representation of the I−V and P–V characteristic curves pertinent to the SDM, DDM, and TDM of the Photowatt PWP-201 PV module obtained by all algorithms has been expounded in Figs. 22, 23, and 24. The quantitative values summarizing the IAE and the RE must be meticulously documented in Table 17. To visually portray the paramount significance of the RE and IAE values ascertained through the instrumentality of the OBEDO and other algorithms, this study has deviously created error graphs as showcased in Figs. 25, 26, and 27. A cursory examination of Figs. 22, 23, 24, 25, 26, and 27 unfolds a noticeably perfect alignment between the estimated and experimental curves. This alignment strengthens the inference that the curve fitting has yielded a worthy equivalence. To offer a comprehensive assessment, meticulous cataloguing of statistical indicators has been meticulously undertaken. These values have been carefully logged within Table 18 to facilitate in-depth data comprehension.

**Table 14:** Parameters estimated for Scenario 3 (SDM).

Algorithms	${I}_{ph}$(A)	${I}_{sd}$(A)	${R}_{se}$(Ω)	${R}_{sh}$(Ω)	$n$	RMSE
OBEDO	1.0305	3.48E−06	1.2013	981.98	48.6428	2.4251E−03
EDO	1.0291	4.85E−06	1.1652	1450.93	49.9469	2.5976E−03
ADHHO	1.0319	3.27E−06	1.2058	834.29	48.4106	2.4492E−03
OBMPA	1.0293	3.92E−06	1.1907	1242.43	49.0939	2.4567E−03
MGTO	1.0305	3.48E−06	1.2013	981.98	48.6428	2.4251E−03
IAOA	1.0287	4.91E−06	1.1642	1492.62	49.9974	2.6154E−03
HDE	1.0305	3.48E−06	1.2013	981.98	48.6428	2.4251E−03
OBGBO	1.0305	3.48E−06	1.2013	981.99	48.6428	2.4251E−03

**Table 15:** Parameters estimated for Scenario 3 (DDM).

Algorithms	${I}_{ph}$(A)	${I}_{sd1}$(A)	${R}_{se}$(Ω)	${R}_{sh}$(Ω)	${n}_{1}$	${I}_{sd2}$(A)	${n}_{2}$	RMSE
OBEDO	1.0305	3.48E−06	1.2013	981.98	48.6428	2.57E−26	6.4861	2.4251E−03
EDO	1.0272	0.00E+00	1.1914	1839.12	9.1993	4.05E−06	49.2168	2.5866E−03
ADHHO	1.0314	0.00E+00	1.1889	916.71	23.4768	3.79E−06	48.9785	2.4770E−03
OBMPA	1.0305	2.44E−06	1.2012	983.01	48.4677	1.06E−06	49.1394	2.4256E−03
MGTO	1.0305	3.48E−06	1.2013	981.98	48.6428	0.00E+00	47.5131	2.4251E−03
IAOA	1.0294	3.62E−07	1.1628	1256.00	49.0419	4.44E−06	49.9928	2.6305E−03
HDE	1.0305	3.48E−06	1.2013	982.00	48.6428	0.00E+00	40.6739	2.4251E−03
OBGBO	1.0305	3.48E−06	1.2013	981.98	48.6428	0.00E+00	1.0000	2.4251E−03

**Table 16:** Parameters estimated for Scenario 3 (TDM).

Algorithms	${I}_{ph}$(A)	${I}_{sd1}$(A)	${R}_{se}$(Ω)	${R}_{sh}$(Ω)	${n}_{1}$	${I}_{sd2}$(A)	${n}_{2}$	${I}_{sd3}$(A)	${n}_{3}$	RMSE
OBEDO	1.0305	1.43E−13	1.2013	982.49	46.764	2.21E−15	49.728	3.48E−06	48.642	2.4251E−03
EDO	1.0321	4.88E−06	1.1586	964.59	49.987	0.00E+00	13.143	0.00E+00	5.306	2.8079E−03
ADHHO	1.0302	4.87E−06	1.1624	1235.76	49.972	7.97E−10	45.552	0.00E+00	1.614	2.6396E−03
OBMPA	1.0300	3.75E−06	1.1935	1084.28	48.928	0.00E+00	35.330	0.00E+00	1.000	2.4342E−03
MGTO	1.0305	3.48E−06	1.2013	981.98	48.643	0.00E+00	49.768	0.00E+00	1.000	2.4251E−03
IAOA	1.0294	0.00E+00	1.1806	1361.22	45.239	1.03E−07	44.828	4.49E−06	50.000	2.5408E−03
HDE	1.0296	3.87E−06	1.1901	1153.42	49.058	8.86E−09	50.000	0.00E+00	36.367	2.4454E−03
OBGBO	1.0305	0.00E+00	1.2013	981.98	49.999	0.00E+00	46.419	3.48E−06	48.643	2.4251E−03

**Table 17:** RE and IAE values attained by OBEDO for Scenario 3.

${V}_{ex}$	${I}_{ex}$	SDM	DDM	TDM	SDM	DDM	TDM	SDM	DDM	TDM
		${I}_{es}$(A)			$RE$			$IAE$(A)		
0.1248	1.0315	1.0291	1.0291	1.0291	2.308E−03	2.308E−03	2.308E−03	2.381E−03	2.381E−03	2.381E−03
1.8093	1.0300	1.0274	1.0274	1.0274	2.543E−03	2.543E−03	2.543E−03	2.619E−03	2.619E−03	2.619E−03
3.3511	1.0260	1.0257	1.0257	1.0257	2.517E−04	2.517E−04	2.517E−04	2.582E−04	2.582E−04	2.582E−04
4.7622	1.0220	1.0241	1.0241	1.0241	− 2.062E−03	− 2.062E−03	− 2.062E−03	2.107E−03	2.107E−03	2.107E−03
6.0538	1.0180	1.0223	1.0223	1.0223	− 4.216E−03	− 4.216E−03	− 4.216E−03	4.292E−03	4.292E−03	4.292E−03
7.2364	1.0155	1.0199	1.0199	1.0199	− 4.363E−03	− 4.363E−03	− 4.363E−03	4.431E−03	4.431E−03	4.431E−03
8.3189	1.0140	1.0164	1.0164	1.0164	− 2.330E−03	− 2.330E−03	− 2.330E−03	2.363E−03	2.363E−03	2.363E−03
9.3097	1.0100	1.0105	1.0105	1.0105	− 4.912E−04	− 4.912E−04	− 4.912E−04	4.962E−04	4.962E−04	4.962E−04
10.2163	1.0035	1.0006	1.0006	1.0006	2.861E−03	2.861E−03	2.861E−03	2.871E−03	2.871E−03	2.871E−03
11.0449	0.9880	0.9845	0.9845	0.9845	3.494E−03	3.494E−03	3.494E−03	3.452E−03	3.452E−03	3.452E−03
11.8018	0.9630	0.9595	0.9595	0.9595	3.612E−03	3.612E−03	3.612E−03	3.478E−03	3.478E−03	3.478E−03
12.4929	0.9255	0.9228	0.9228	0.9228	2.875E−03	2.875E−03	2.875E−03	2.661E−03	2.661E−03	2.661E−03
13.1231	0.8725	0.8726	0.8726	0.8726	− 1.142E−04	− 1.142E−04	− 1.142E−04	9.966E−05	9.966E−05	9.966E−05
13.6983	0.8075	0.8073	0.8073	0.8073	2.795E−04	2.795E−04	2.796E−04	2.257E−04	2.257E−04	2.257E−04
14.2221	0.7265	0.7283	0.7283	0.7283	− 2.528E−03	− 2.528E−03	− 2.528E−03	1.836E−03	1.836E−03	1.836E−03
14.6995	0.6345	0.6371	0.6371	0.6371	− 4.158E−03	− 4.158E−03	− 4.158E−03	2.638E−03	2.638E−03	2.638E−03
15.1346	0.5345	0.5362	0.5362	0.5362	− 3.205E−03	− 3.205E−03	− 3.205E−03	1.713E−03	1.713E−03	1.713E−03
15.5311	0.4275	0.4295	0.4295	0.4295	− 4.705E−03	− 4.705E−03	− 4.705E−03	2.011E−03	2.011E−03	2.011E−03
15.8929	0.3185	0.3188	0.3188	0.3188	− 8.618E−04	− 8.618E−04	− 8.618E−04	2.745E−04	2.745E−04	2.745E−04
16.2229	0.2085	0.2074	0.2074	0.2074	5.326E−03	5.326E−03	5.326E−03	1.110E−03	1.110E−03	1.110E−03
16.5241	0.1010	0.0962	0.0962	0.0962	4.785E−02	4.785E−02	4.785E−02	4.833E−03	4.833E−03	4.833E−03
16.7987	− 0.0080	− 0.0083	− 0.0083	− 0.0083	− 4.067E−02	− 4.067E−02	− 4.067E−02	3.254E−04	3.254E−04	3.254E−04
17.0499	− 0.1110	− 0.1109	− 0.1109	− 0.1109	5.722E−04	5.722E−04	5.722E−04	6.352E−05	6.352E−05	6.352E−05
17.2793	− 0.2090	− 0.2092	− 0.2092	− 0.2092	− 1.183E−03	− 1.183E−03	− 1.183E−03	2.473E−04	2.473E−04	2.473E−04
17.4885	− 0.3030	− 0.3009	− 0.3009	− 0.3009	7.051E−03	7.051E−03	7.051E−03	2.136E−03	2.136E−03	2.136E−03
Mean values					3.253E−04	3.253E−04	3.253E−04	1.957E−03	1.957E−03	1.957E−03

**Table 18:** Performance Comparison for Scenario 3.

Model	Algorithms	${\text{Min}}$	${\text{Max}}$	${\text{Mean}}$	${\text{Median}}$	${\text{STD}}$	${\text{RT}}$
SDM	**OBEDO**	**2.4251E−03**	**2.4251E−03**	**2.4251E−03**	**2.4251E−03**	**5.7466E−17**	8.60
	EDO	2.5976E−03	2.7425E−01	7.4780E−02	1.4859E−02	1.0842E−01	**8.42**
	ADHHO	2.4492E−03	2.7425E−01	4.1301E−02	6.1914E−03	8.6647E−02	8.73
	OBMPA	2.4567E−03	2.6620E−03	2.5238E−03	2.5092E−03	7.1505E−05	8.59
	MGTO	2.4251E−03	2.7425E−01	2.9608E−02	2.4251E−03	8.5959E−02	8.75
	IAOA	2.6154E−03	6.8327E−03	3.6133E−03	3.1613E−03	1.3100E−03	8.94
	HDE	2.4251E−03	2.4315E−03	2.4257E−03	2.4251E−03	2.0398E−06	8.72
	OBGBO	2.4251E−03	2.4253E−03	2.4251E−03	2.4251E−03	7.2685E−08	78.86
DDM	**OBEDO**	**2.4251E−03**	**2.4252E−03**	**2.4251E−03**	**2.4251E−03**	**4.6067E−08**	8.09
	EDO	2.5866E−03	2.7425E−01	1.0649E−01	7.2955E−02	1.1961E−01	**7.98**
	ADHHO	2.4770E−03	2.7425E−01	3.6645E−02	3.4096E−03	8.4686E−02	8.18
	OBMPA	2.4256E−03	1.0282E−02	3.2765E−03	2.4984E−03	2.4620E−03	8.20
	MGTO	2.4251E−03	2.7425E−01	8.4172E−02	2.4281E−03	1.3117E−01	8.42
	IAOA	2.6305E−03	1.0384E−02	4.9402E−03	3.5194E−03	2.6403E−03	8.18
	HDE	2.4251E−03	2.5321E−03	2.4554E−03	2.4283E−03	4.3904E−05	8.19
	OBGBO	2.4251E−03	2.5443E−03	2.4484E−03	2.4252E−03	4.8421E−05	75.52
TDM	**OBEDO**	**2.4251E−03**	**2.4271E−03**	**2.4253E−03**	**2.4251E−03**	**6.3319E−07**	**8.09**
	EDO	2.8079E−03	2.7427E−01	1.5276E−01	1.7360E−01	1.3053E−01	7.91
	ADHHO	2.6396E−03	1.4802E−01	2.4883E−02	1.1029E−02	4.4208E−02	8.25
	OBMPA	2.4342E−03	5.0936E−03	2.7775E−03	2.5012E−03	8.1712E−04	8.13
	MGTO	2.4251E−03	2.7425E−01	1.1134E−01	3.3245E−03	1.4022E−01	8.60
	IAOA	2.5408E−03	8.7220E−03	3.9698E−03	3.5730E−03	1.8878E−03	8.21
	HDE	2.4454E−03	2.5653E−03	2.5183E−03	2.5245E−03	3.5846E−05	8.26
	OBGBO	2.4251E−03	1.1247E−01	1.3448E−02	2.4388E−03	3.4794E−02	75.26

Significant values are in [bold].

The IAE value resulting from using the OBEDO in the context of the SDM, DDM, and TDM for scenario 2 is notably below the threshold of 5.12E−04. This indicates a remarkably precise alignment between the predicted and observed values. Similarly, the RE value, which gauges the degree of dissimilarity between the predicted and actual values, demonstrates significant conformity as it remains under the stringent limit of 2.41E−02. The average RE and IAE values of SDM, DDM, and TDM are − 3.253E−04, 3.253E−04, 3.253E−04, 1.957E−03, 1.957E−03, and 1.957E−03, respectively, further reinforcing the efficacy of the optimization algorithm in achieving a robust convergence between the simulated and empirical outcomes. The comprehensive data is showcased in Table 17 and Figs. 25, 26, and 27 corroborates the capability of the OBEDO to precisely extract the intricate characteristics of the PV module within the specific framework. This affirmation of accuracy underscores the algorithm's competence in capturing the nuanced behaviour and performance attributes of the PV module, thereby contributing to a deeper and more comprehensive understanding of its indefinite characteristics.

Table 18 provides a comprehensive overview of algorithmic performance across three photovoltaic models: SDM, DDM, and TDM. Key metrics are presented for each algorithm within these models. Across the PV models, a diverse array of algorithmic behaviour is evident. OBEDO consistently demonstrates remarkable accuracy with low RMSE values, indicating precise predictions. EDO shows broader variability in RMSE values, suggesting varying predictive precision. ADHHO and OBMPA both exhibit stable RMSE values, implying reliable performance. MGTO's consistently low RMSE values suggest accurate predictions, while IAOA displays variability, particularly in SDM and DDM. HDE consistently maintains low RMSE values across models, reflecting dependable performance. Notably, OBGBO presents low RMSE values, albeit with higher runtimes. Within the DDM model, OBEDO stands out with its low RMSE values and efficient runtime. In TDM, OBEDO showcases impressive accuracy, while EDO displays variability. ADHHO and OBMPA offer stable and reliable performance. MGTO and IAOA present varying precision, while HDE demonstrates consistent accuracy. OBGBO maintains its performance pattern with higher runtimes. Overall, OBEDO, MGTO, and HDE consistently exhibit robust performance across various PV models, with OBEDO excelling in accuracy and efficiency. The findings emphasize the intricate interplay between accuracy and runtime, underscoring the significance of a comprehensive evaluation of algorithmic efficacy in PV modelling.

**Figure 28:**
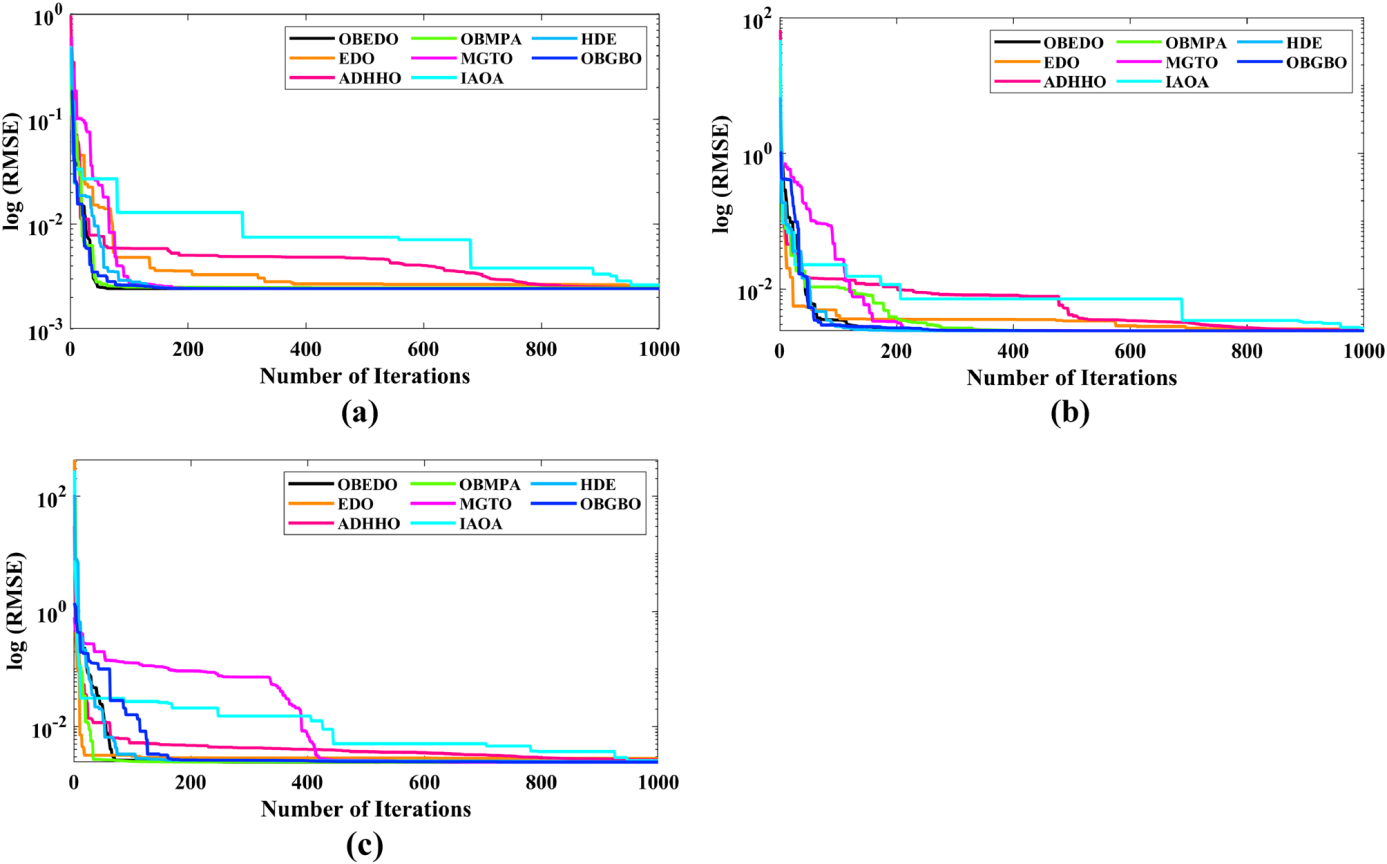
Convergence curves (Scenario 3); (**a**) SDM, (**b**) DDM, (**c**) TDM.

**Figure 29:**
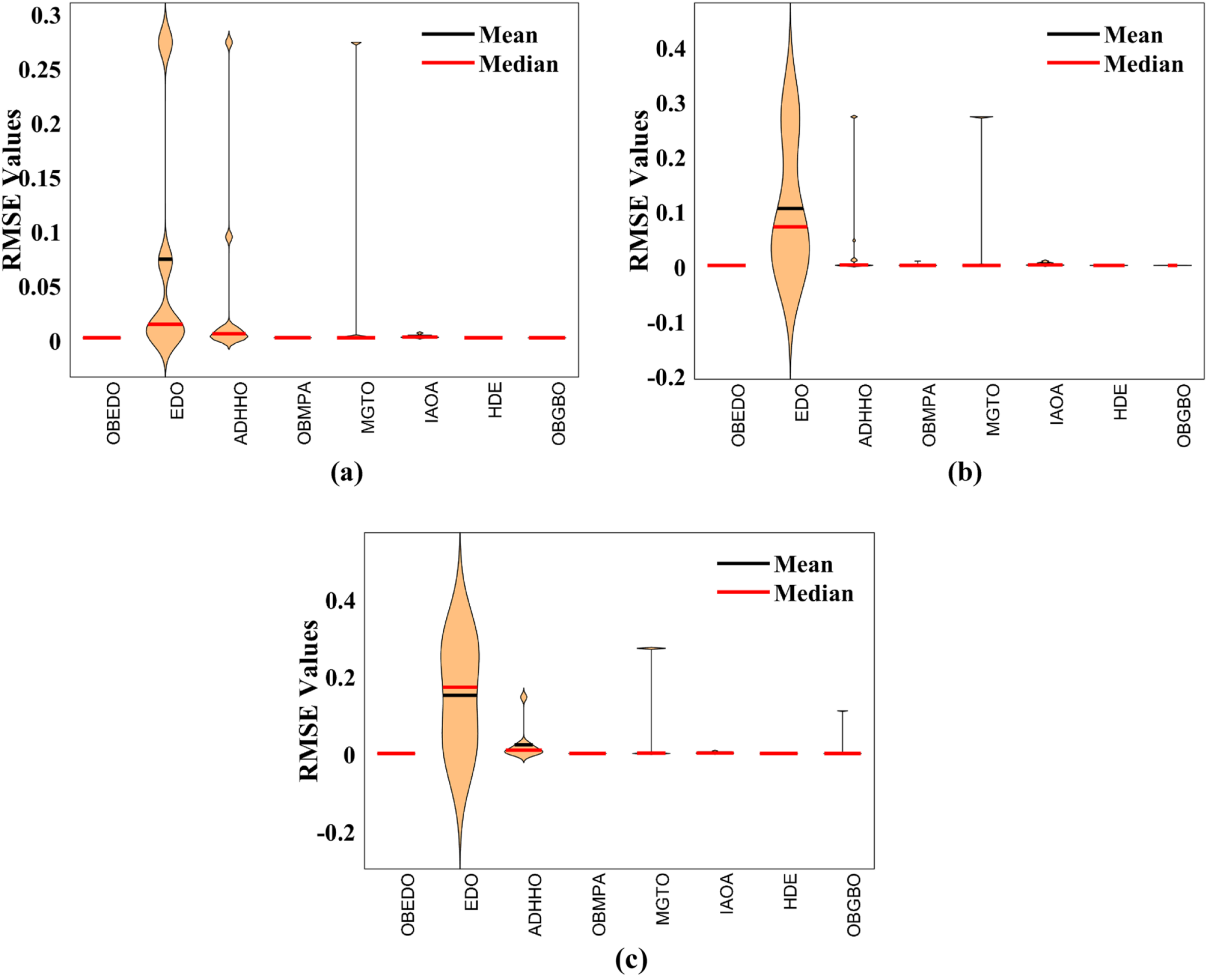
Violin plots (Scenario 3); (**a**) SDM, (**b**) DDM, (**c**) TDM.

The convergence dynamics of the SDM, DDM, and TDM for Scenario 3 are meticulously unveiled through intricate progression trajectories, portrayed vividly in Fig. 28. Upon inspecting this visual depiction, the rapid and impressive convergence of OBEDO becomes clearly evident. This accelerated trajectory surpasses its algorithmic counterparts, underscoring OBEDO's exceptional efficiency. The narrative takes an artistic turn with Fig. 29, which employs the visually captivating violin plot.

The strategies defined for SDM, DDM, and TDM play leading roles, each contributing a distinct note to the symphony of data visualization. Within the realm of the symphony of violin plots, a remarkable revelation unfolds—OBEDO emerges as a virtuoso of reliability. The attentive observer discerns the delicate undercurrents of STD values. The low STD values resonate across all photovoltaic models in Scenario 3. This steadfast stability further solidifies OBEDO's stature as an unmatched performer, towering among its algorithmic peers.

### Scenario 4: commercial sharp ND− R250A5 polycrystalline PV module

**Figure 30:**
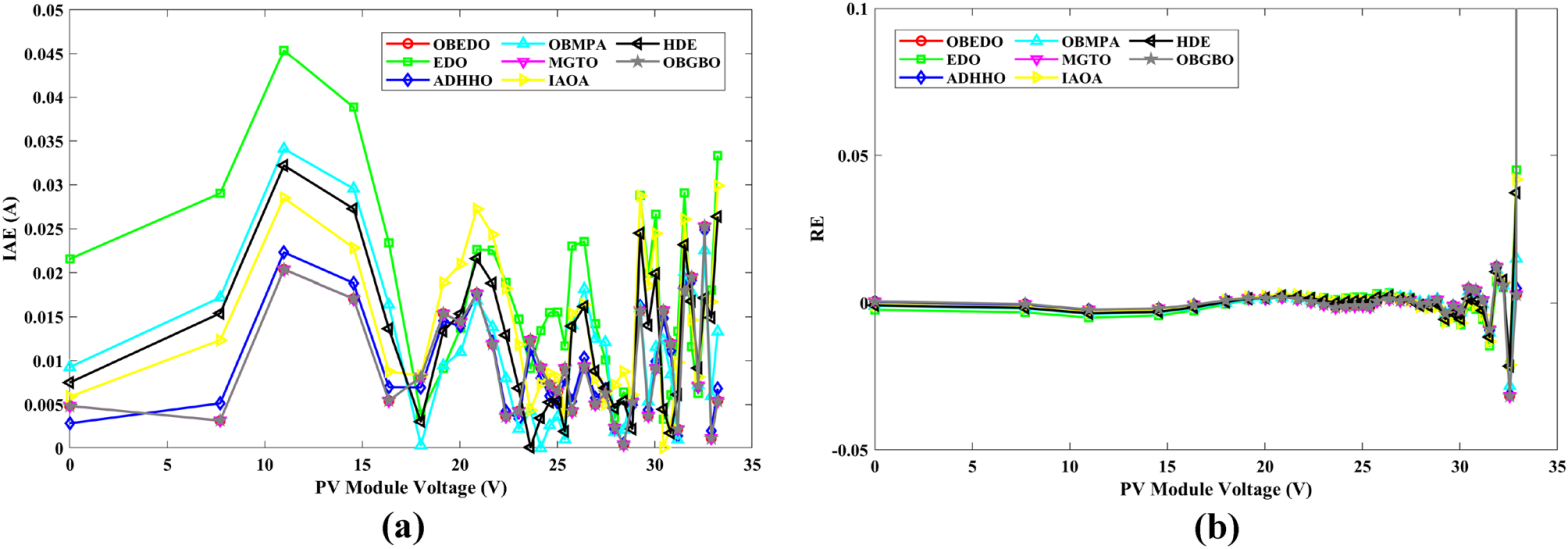
Error characteristics of Sharp ND-R250A5 PV module; (**a**) IAE, (**b**) RE.

**Figure 31:**
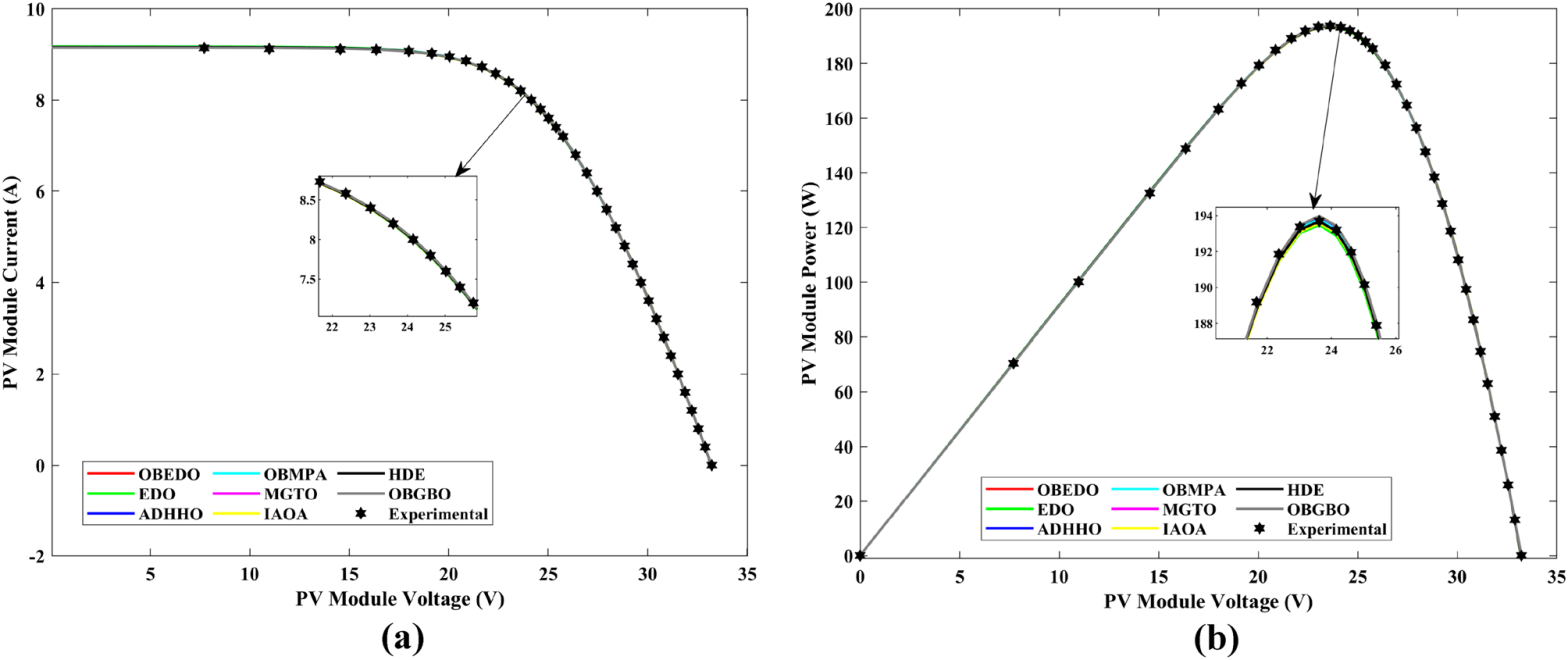
Characteristics of Sharp ND-R250A5 PV module; (**a**) I–V characteristics, (**b**) P–V characteristics.

**Figure 32:**
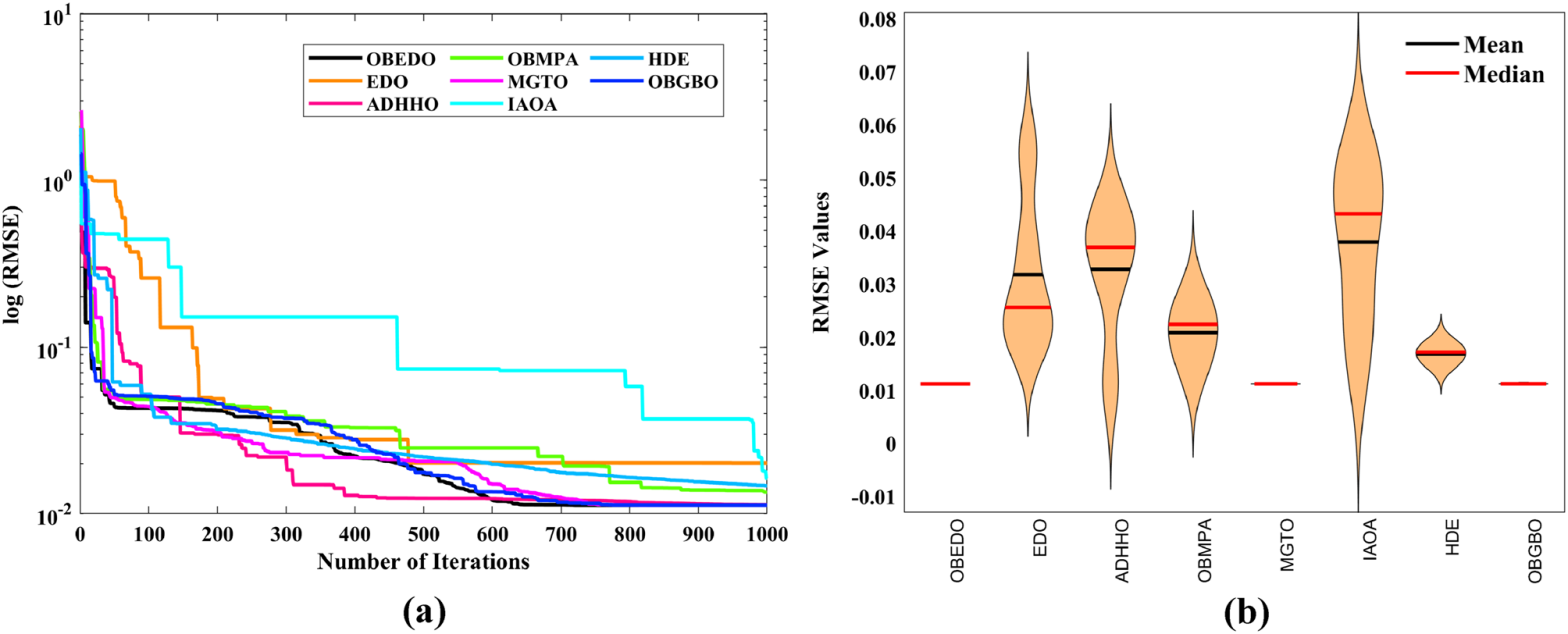
Statistical metrics of Sharp ND-R250A5 PV module; (**a**) convergence curves, (**b**) violin plots.

The I–V characteristic of this panel has been measured at a temperature of 59 °C and an irradiation of 1040 W/m^2^. The results obtained for SDM of the commercial Sharp ND-R250A5 polycrystalline PV module are discussed in this sub-section. This PV panel consists of 60 polycrystalline silicon PV cells that are connected in series. Table 19 presents the optimized parameters of the commercial Sharp ND-R250A5 polycrystalline PV module by all selected algorithms. Table 20 presents the recorded data points, estimated data points using the OBEDO, and the corresponding errors. The average values for IAE and RE are stated as 9.41E−03 and − 3.93e−04. Table 2 displays the boundary values, and Table 19 displays the estimated optimal parameters of the PV module calculated by the OBEDO. The statistical measures for the SDM of this PV panel are presented in Table 21. The optimal RMSE is 1.12E−02, while the IAE is 9.41e−03. The results of five parameters derived by OBEDO were given in Table 19 and were compared with the other statE−of-thE−art algorithms. When comparing the outputs of the SDM, it is evident that the SDM obtained by OBEDO has lower RMSE values compared to the other algorithms. The error characteristics of this case are visualized in Fig. 30. Figure 30a illustrates the IAE with respect to the measured voltage, and Fig. 30b illustrates the RE with respect to the measured voltage. The measured and estimated I–V characteristics for the SDM of this PV panel are depicted in Fig. 31a. The P–V characteristics of this PV panel are shown in Fig. 31b, both measured and computed. The convergence of the objective function and violin plot analysis for OBEDO and seven other optimisation strategies is shown in Fig. 32. The convergence speed of the OBEDO surpasses that of the other algorithms, as demonstrated by Fig. 32a. The reliability of all the algorithms is visualized in Fig. 32b.

**Table 19:** Parameters estimated for Scenario 4.

Algorithms	${I}_{ph}$(A)	${I}_{sd}$(A)	${R}_{se}$(Ω)	${R}_{sh}$(Ω)	$n$	RMSE
OBEDO	**9.1461**	**1.09E−06**	**0.5895**	**5500.00**	**72.8007**	**1.12E−02**
EDO	9.1727	2.32E−06	0.5725	4564.75	76.3866	2.01E−02
ADHHO	9.1481	1.14E−06	0.5886	5499.78	73.0083	1.13E−02
OBMPA	9.1603	1.48E−06	0.5836	5499.97	74.2254	1.34E−02
MGTO	9.1461	1.09E−06	0.5895	5500.00	72.8007	1.12E−02
IAOA	9.1579	1.73E−06	0.5779	2712.83	74.9546	1.63E−02
HDE	9.1585	1.67E−06	0.5796	5500.00	74.7775	1.47E−02
OBGBO	9.1462	1.09E−06	0.5895	5500.00	72.8006	1.12E−02

Significant values are in [bold].

**Table 20:** RE and IAE values attained by OBEDO for Scenario 4.

${V}_{ex}$	${I}_{ex}$	${I}_{es}$(A)	$RE$	$IAE$(A)
0.00	9.15	9.1451	5.31E−04	4.86E−03
7.71	9.14	9.1432	− 3.47E−04	3.17E−03
10.98	9.12	9.1404	− 2.23E−03	2.04E−02
14.55	9.11	9.1271	− 1.87E−03	1.71E−02
16.36	9.10	9.1054	− 5.98E−04	5.44E−03
18.00	9.07	9.0618	9.00E−04	8.16E−03
19.15	9.02	9.0046	1.70E−03	1.54E−02
20.04	8.95	8.9356	1.61E−03	1.44E−02
20.87	8.86	8.8423	1.99E−03	1.77E−02
21.67	8.73	8.7181	1.36E−03	1.19E−02
22.36	8.58	8.5763	4.27E−04	3.66E−03
23.02	8.40	8.4043	− 5.07E−04	4.26E−03
23.62	8.20	8.2124	− 1.51E−03	1.24E−02
24.15	8.00	8.0092	− 1.15E−03	9.22E−03
24.61	7.80	7.8072	− 9.29E−04	7.24E−03
25.02	7.60	7.6065	− 8.56E−04	6.51E−03
25.39	7.40	7.4091	− 1.24E−03	9.14E−03
25.75	7.20	7.1958	5.89E−04	4.24E−03
26.38	6.80	6.7906	1.38E−03	9.37E−03
26.94	6.40	6.3950	7.87E−04	5.04E−03
27.46	6.00	5.9937	1.05E−03	6.30E−03
27.94	5.60	5.6024	− 4.24E−04	2.38E−03
28.40	5.20	5.2004	− 7.89E−05	4.10E−04
28.84	4.80	4.7947	1.10E−03	5.30E−03
29.25	4.40	4.4157	− 3.58E−03	1.57E−02
29.66	4.00	4.0037	− 9.34E−04	3.74E−03
30.05	3.60	3.6092	− 2.54E−03	9.16E−03
30.44	3.20	3.1843	4.91E−03	1.57E−02
30.81	2.80	2.7880	4.28E−03	1.20E−02
31.17	2.40	2.3978	8.96E−04	2.15E−03
31.52	2.00	2.0180	− 8.99E−03	1.80E−02
31.88	1.60	1.5805	1.22E−02	1.95E−02
32.22	1.20	1.1928	5.98E−03	7.17E−03
32.55	0.80	0.8253	− 3.16E−02	2.53E−02
32.89	0.40	0.3989	2.87E−03	1.15E−03
33.22	0.00	− 0.0054	6.55E−04	5.43E−03
Average values			− 3.93e−04	9.41E−03

**Table 21:** Performance comparison for Scenario 4.

Algorithms	${\text{Min}}$	${\text{Max}}$	${\text{Mean}}$	${\text{Median}}$	${\text{STD}}$	${\text{RT}}$
**OBEDO**	**1.1245E−02**	**1.1245E−02**	**1.1245E−02**	**1.1245E−02**	**2.3551E−10**	7.275
EDO	2.0110E−02	5.5021E−02	3.1794E−02	2.5610E−02	1.4637E−02	**7.125**
ADHHO	1.1291E−02	4.4144E−02	3.2799E−02	3.6944E−02	1.2950E−02	7.875
OBMPA	1.3433E−02	2.7885E−02	2.0930E−02	2.2408E−02	5.5314E−03	7.319
MGTO	1.1245E−02	1.1441E−02	1.1317E−02	1.1254E−02	9.5422E−05	7.409
IAOA	1.6290E−02	5.1827E−02	3.7945E−02	4.3235E−02	1.5270E−02	7.306
HDE	1.4721E−02	1.8955E−02	1.6927E−02	1.7218E−02	1.7462E−03	7.734
OBGBO	1.1245E−02	1.1468E−02	1.1298E−02	1.1266E−02	9.5245E−05	63.741

Significant values are in [bold].

### Scenario 5—commercial SM55 PV module

This sub-section details the results obtained by the proposed algorithm and other algorithms for scenario 5, i.e., the SDM of the SM55 PV module. The bounds for this scenario are provided in Table 2. To extract the parameters of scenario 5, a comprehensive analysis is conducted using experimental I–V samples. These samples are gathered under specific conditions, encompassing constant irradiance (G) of 1000 W/m^2^ and varying temperatures (T) of 25 °C, 50 °C, and 75 °C, as well as a constant temperature of 25 °C and diverse irradiation levels including 1000 W/m^2^, 800 W/m^2^, 600 W/m^2^, 400 W/m^2^, and 200 W/m^2^. The evaluation process encompasses various statistical measures and other pertinent performance indicators. Equation (30) is pivotal in this context, as it calculates the specific photovoltaic modules short-circuit current I_sc_ across various operational scenarios.

30 $$I_{sc} \left( {G,T} \right) = I_{{sc\left( {STC} \right)}} \times \frac{G}{{G_{STC} }} + \alpha \times \left( {T - T_{STC} } \right)$$

In the context of this study, the variables T and G hold significance as they denote the prevailing temperature and irradiation levels. The parameter α signifies the temperature coefficient, while T_STC_ represents the temperature at STC, and G_STC_ corresponds to the irradiance at STC. Additionally, I_(sc(STC))_ represents the short-circuit current at STC. Through this meticulous examination, insights are gained into the behaviour and adaptability of the PV module model, facilitating a nuanced understanding of its performance under varying temperature and irradiance conditions. This analysis contributes to a comprehensive comprehension of the model's capabilities, aiding in optimizing and deploying photovoltaic systems across a spectrum of real-world scenarios.

Within this study, Tables 22 and 23 play a pivotal role in offering a comprehensive insight into the parameters estimated and optimized using the OBEDO, alongside several alternative methodologies employed to investigate the PV module model. Delving into the specifics, Table 22 meticulously catalogues the estimated variables associated with the SM55 photovoltaic module. The entries in this table pertain to scenarios where the irradiance remains constant at 1000 W/m^2^ while the temperature fluctuates across three distinct levels: 25 °C, 50 °C, and 75 °C. Meanwhile, Table 23 presents a similar compilation of estimated parameters for the SM55 PV module under differing conditions. In this instance, the temperature is maintained at a constant 25 °C while the irradiance levels are varied, spanning the range of 1000–200 W/m^2^.

**Table 22:** Parameters estimated by the algorithms (Scenario 4) under various temperature conditions.

Temperature	Algorithm	${I}_{ph}$(A)	${I}_{sd}$(A)	${R}_{se}$(Ω)	${R}_{sh}$(Ω)	$n$	RMSE
60 °C	**OBEDO**	**3.4946**	**6.91E−06**	**0.3187**	**484.88**	**1.4051**	**3.7804E−03**
	EDO	3.4785	5.75E−06	0.3335	4811.41	1.3853	8.6436E−03
	ADHHO	3.4903	3.82E−05	0.2248	5000.00	1.6138	1.2592E−02
	OBMPA	3.4829	1.03E−05	0.3064	4980.33	1.4486	6.0617E−03
	MGTO	3.4946	6.91E−06	0.3187	484.88	1.4051	3.7804E−03
	IAOA	3.4933	3.22E−05	0.2400	1863.55	1.5905	1.1925E−02
	HDE	3.4905	1.15E−05	0.2954	875.64	1.4609	4.9433E−03
	OBGBO	3.4946	6.91E−06	0.3187	484.88	1.4051	3.7804E−03
40 °C	**OBEDO**	**3.4691**	**1.15**E−**06**	**0.3131**	**533.07**	**1.4178**	**3.7888E−03**
	EDO	3.4674	9.19E−06	0.2120	1577.15	1.6465	1.4522E−02
	ADHHO	3.4649	1.08E−05	0.2048	5000.00	1.6668	1.5092E−02
	OBMPA	3.4596	3.88E−06	0.2602	4987.71	1.5429	8.2396E−03
	MGTO	3.4686	1.20E−06	0.3113	548.28	1.4221	3.7986E−03
	IAOA	3.4752	1.09E−05	0.1825	604.29	1.6684	1.9305E−02
	HDE	3.4593	3.39E−06	0.2696	5000.00	1.5282	7.5246E−03
	OBGBO	3.4664	1.23E−06	0.3110	629.96	1.4244	3.9181E−03
25 °C	**OBEDO**	**3.4501**	**1.71**E−**07**	**0.3291**	**483.90**	**1.3958**	**1.1462E−03**
	EDO	3.4496	1.18E−05	0.1258	3571.99	1.8625	2.5065E−02
	ADHHO	3.4459	6.94E−06	0.1566	5000.00	1.7871	2.0923E−02
	OBMPA	3.4404	1.80E−06	0.2347	5000.00	1.6210	1.2037E−02
	MGTO	3.4435	5.91E−07	0.2834	1320.00	1.5055	5.8714E−03
	IAOA	3.4644	4.28E−06	0.1941	431.22	1.7257	2.4107E−02
	HDE	3.4400	1.81E−06	0.2285	5000.00	1.6218	1.2356E−02
	OBGBO	3.4457	2.26E−07	0.3207	658.48	1.4191	2.2319E−03

Significant values are in [bold].

**Table 23:** Parameters estimated by the algorithms (Scenario 4) under irradiation conditions.

Temperature	Algorithm	${I}_{ph}$(A)	${I}_{sd}$(A)	${R}_{se}$(Ω)	${R}_{sh}$(Ω)	$n$	RMSE
1000 W/m^2^	**OBEDO**	**3.4501**	**1.71E−07**	**0.3291**	**483.90**	**1.3958**	**1.1462E−03**
	EDO	3.4478	1.61E−05	0.0789	2976.81	1.9075	2.8393E−02
	ADHHO	3.4492	1.46E−05	0.1056	5000.00	1.8940	2.6396E−02
	OBMPA	3.4420	2.61E−06	0.2154	4999.82	1.6637	1.4319E−02
	MGTO	3.4384	5.10E−07	0.2864	1732.88	1.4915	5.4408E−03
	IAOA	3.4457	5.71E−06	0.1567	1577.93	1.7608	2.0753E−02
	HDE	3.4409	1.96E−06	0.2315	4998.04	1.6310	1.2598E−02
	OBGBO	3.4483	1.89E−07	0.3263	537.09	1.4040	1.3549E−03
800 W/m^2^	**OBEDO**	**2.7604**	**1.44E−07**	**0.3376**	**459.88**	**1.3811**	**6.6858E−04**
	EDO	2.7494	4.68E−06	0.0781	4851.15	1.7330	1.0357E−02
	ADHHO	2.7504	5.62E−06	0.0693	5000.00	1.7575	1.0853E−02
	OBMPA	2.7491	3.41E−06	0.1169	4958.55	1.6946	9.2702E−03
	MGTO	2.7531	4.82E−07	0.2663	757.15	1.4862	2.6706E−03
	IAOA	2.7570	1.37E−05	0.0103	2888.19	1.8858	1.5824E−02
	HDE	2.7518	1.52E−06	0.1859	1351.23	1.6023	6.8257E−03
	OBGBO	2.0709	1.56E−07	0.3305	450.07	1.3875	8.2395E−04
600 W/m^2^	**OBEDO**	**2.0676**	**2.99E−07**	**0.2873**	**571.22**	**1.4449**	**1.8937E−03**
	EDO	2.0613	4.80E−06	0.0635	3611.34	1.7502	9.4736E−03
	ADHHO	2.0655	1.03E−06	0.1882	785.35	1.5666	4.9593E−03
	OBMPA	2.0578	1.69E−06	0.1621	4650.37	1.6209	7.1003E−03
	MGTO	2.7584	1.69E−07	0.3306	521.22	1.3945	1.1001E−03
	IAOA	2.0654	8.33E−06	0.0636	3891.52	1.8302	1.5062E−02
	HDE	2.0654	1.30E−06	0.1645	851.54	1.5908	5.6085E−03
	OBGBO	2.0699	1.66E−07	0.3271	472.82	1.3931	9.1622E−04
400 W/m^2^	**OBEDO**	**1.3828**	**1.00E−07**	**0.3966**	**427.07**	**1.3520**	**7.0761E−04**
	EDO	1.3710	4.28E−06	0.0286	4968.53	1.7486	8.4576E−03
	ADHHO	1.3776	1.71E−06	0.0000	687.21	1.6270	3.7836E−03
	OBMPA	1.3789	1.25E−06	0.0001	574.57	1.5892	3.3034E−03
	MGTO	1.3828	1.02E−07	0.3945	428.48	1.3533	7.0860E−04
	IAOA	1.3738	2.28E−06	0.0013	1140.29	1.6624	4.9593E−03
	HDE	1.3797	6.08E−07	0.1437	535.90	1.5138	2.3359E−03
	OBGBO	1.3811	1.51E−07	0.3447	462.22	1.3856	1.0206E−03
200 W/m^2^	**OBEDO**	**0.6920**	**1.31E−07**	**0.3124**	**438.04**	**1.3709**	**5.2054E−04**
	EDO	0.6808	2.13E−06	0.0262	2031.57	1.6735	5.9319E−03
	ADHHO	0.6904	4.83E−07	0.0000	495.17	1.4954	1.1563E−03
	OBMPA	0.6907	3.54E−07	0.0000	471.10	1.4622	8.0751E−04
	MGTO	0.6920	1.31E−07	0.3124	438.05	1.3709	5.2054E−04
	IAOA	0.6894	2.11E−06	0.0000	637.22	1.6768	4.9016E−03
	HDE	0.6912	2.62E−07	0.0944	456.08	1.4334	6.7018E−04
	OBGBO	0.6920	1.32E−07	0.3107	438.11	1.3714	5.2055E−04

Significant values are in [bold].

**Figure 33:**
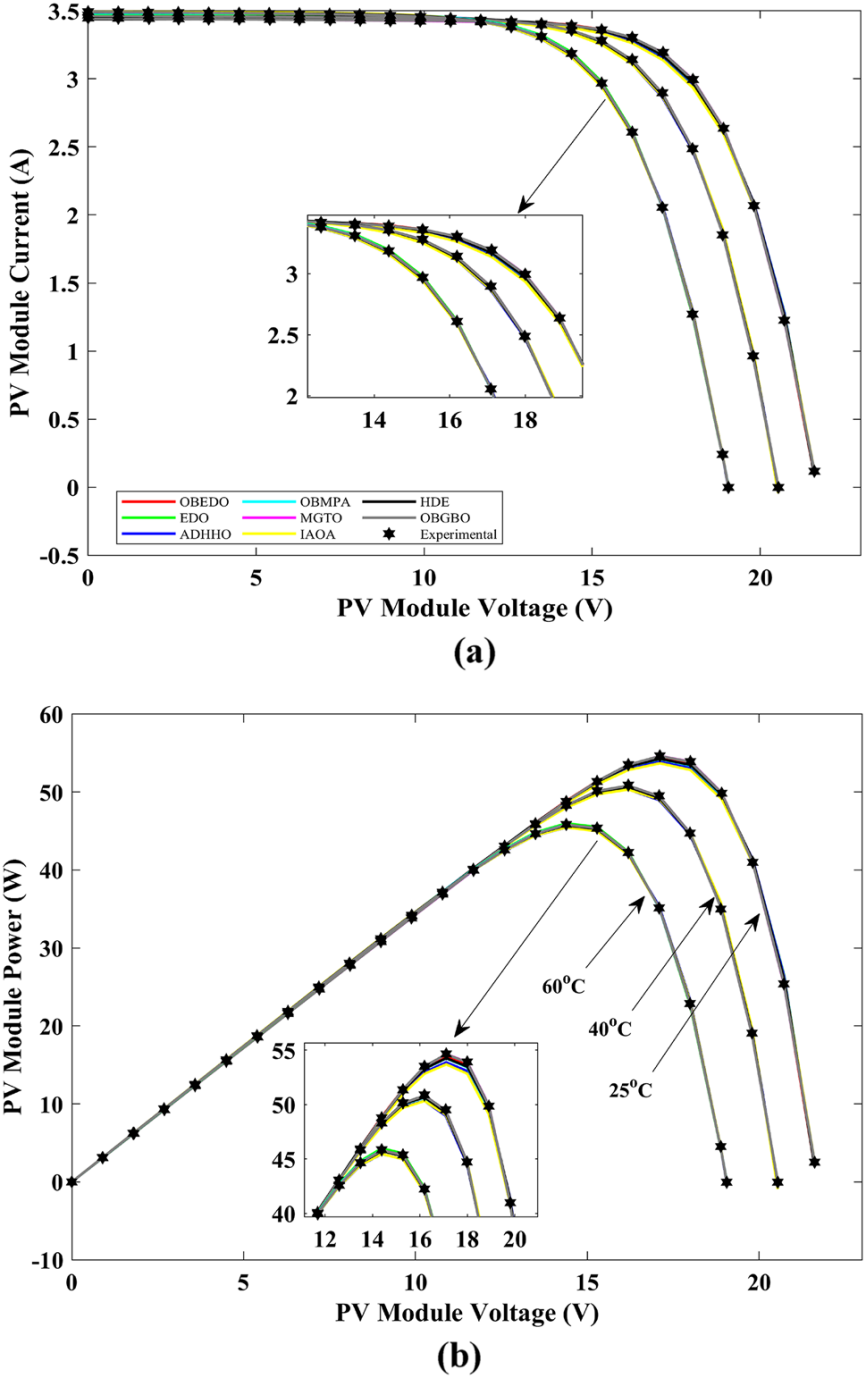
Characteristics of the SM55 PV module under different temperature conditions obtained by all algorithms; (**a**) I–V curves, (**b**) P–V curves.

**Figure 34:**
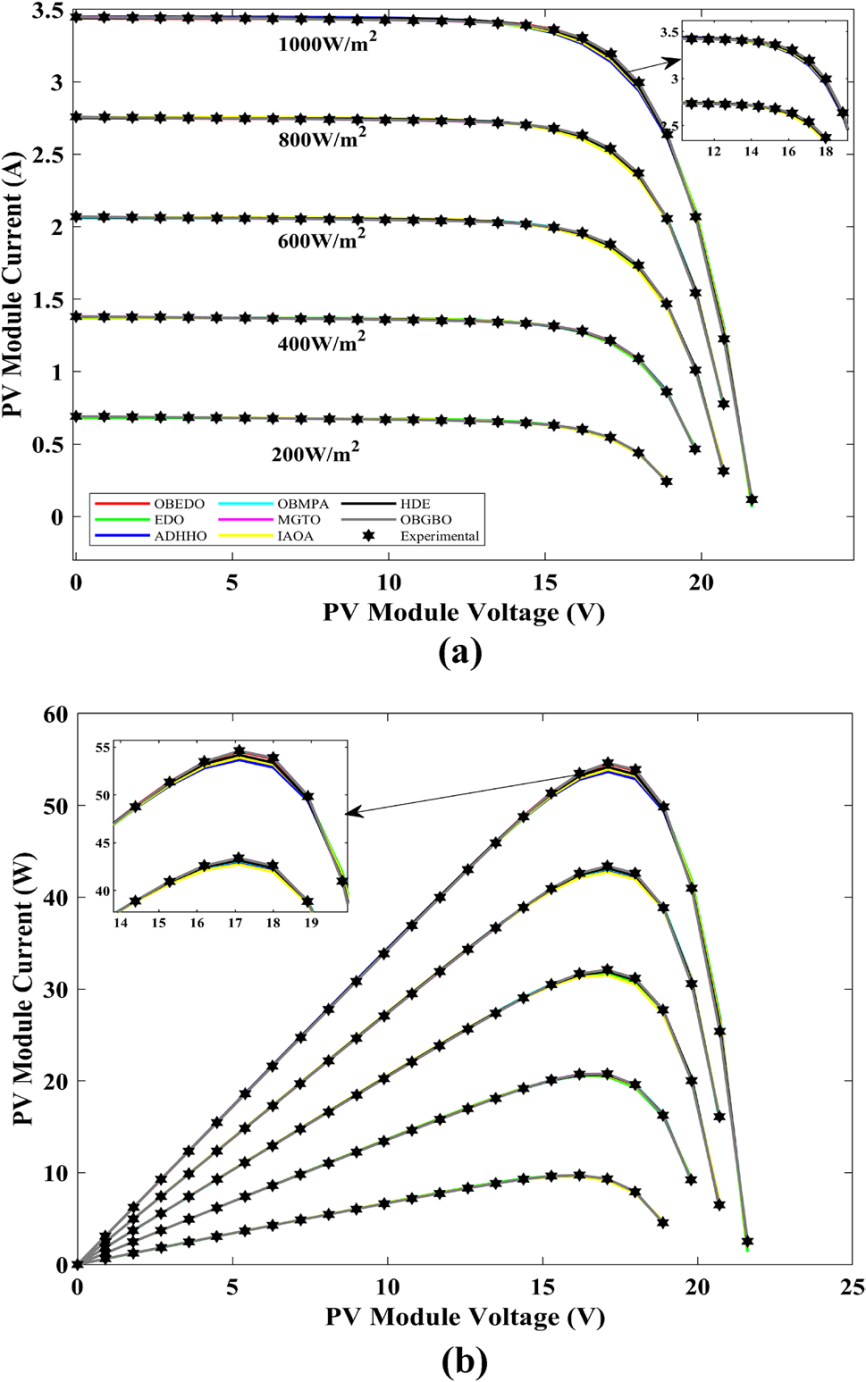
Characteristics of the SM55 PV module under different irradiance conditions obtained by all algorithms; (**a**) I–V curves, (**b**) P–V curves.

The outcomes in Tables 22 and 23 witness the superior predictive prowess of the proposed OBEDO algorithm. These assertions are substantiated by observing significant RMSE values. This quantifiable measure of accuracy underscores the algorithm's efficacy in determining and predicting the parameters intrinsic to a PV system with enhanced precision. Augmenting the tabular data, Figs. 33 and 34 provide a graphical representation of the I–V and P–V curves, depicting the behaviour of the SM55 PV module as extracted through the OBEDO and other algorithms. These curves offer a tangible means to observe the alignment and coherence between the curves generated from the algorithm's predicted data and the actual empirical data. This visual correlation elucidates how much the algorithmic predictions harmonize with real-world observations, enhancing our understanding of the model's performance and predictive capabilities.

In order to conduct a more comprehensive evaluation of the performance of the newly introduced OBEDO, a detailed analysis of its statistical measures was carried out. This analysis specifically focused on Scenario 5 and involved the computation of various key metrics, including the Min, Max, Mean, Median and STD values. These statistical results were subsequently organized and presented in a tabular format, with Tables 24 and 25 dedicated to showcasing these values. The investigation was undertaken across various environmental factors, encompassing different temperature settings and varying irradiation conditions. The purpose of this exploration was to measure the algorithm's robustness and effectiveness under a variety of scenarios. Notably, the outcomes yielded by the OBEDO exhibited remarkable performance in terms of the computed statistical measures, specifically the Min, Max, Mean, and STD values. This notable achievement can be attributed to incorporating a unique enhancement technique, namely integrating OBL within the algorithmic framework. By infusing the algorithm with the principles of OBL, the inherent capacity of the OBEDO to explore and exploit the solution space was significantly augmented. This enhancement played a crucial role in the algorithm's ability to search for optimal PV module parameters under varying conditions efficiently.

**Table 24:** Statistical analysis of Scenario 4 at different temperatures.

Temperature	Algorithm	$Min$	$Max$	$Mean$	$Median$	$STD$	$RT$
25 °C	**OBEDO**	**1.1462E−03**	**1.8005E−03**	**1.2771E−03**	**1.1462E−03**	**2.9262E−04**	8.85
	EDO	2.5065E−02	3.7995E−02	3.2204E−02	3.1091E−02	5.0967E−03	8.69
	ADHHO	2.0923E−02	3.6679E−02	3.1508E−02	3.3408E−02	6.2750E−03	8.98
	OBMPA	1.2037E−02	3.5555E−02	2.3128E−02	1.9586E−02	1.1714E−02	9.13
	MGTO	5.8714E−03	7.6867E−03	7.0764E−03	7.2310E−03	7.4682E−04	8.98
	IAOA	2.4107E−02	3.9259E−02	3.2205E−02	3.3638E−02	5.5939E−03	8.88
	HDE	1.2356E−02	1.5187E−02	1.3720E−02	1.3832E−02	1.0441E−03	9.36
	OBGBO	2.2319E−03	6.4832E−03	5.4702E−03	6.3865E−03	1.8339E−03	79.80
40 °C	**OBEDO**	**3.7888E−03**	**3.7929E−03**	**3.7896E−03**	**3.7888E−03**	**1.8064E−06**	8.00
	EDO	1.4522E−02	5.0768E−02	3.4681E−02	3.6563E−02	1.4167E−02	**7.92**
	ADHHO	1.5092E−02	2.9463E−02	2.2617E−02	2.2168E−02	6.1953E−03	8.30
	OBMPA	8.2396E−03	1.6600E−02	1.2395E−02	1.2379E−02	3.6711E−03	8.21
	MGTO	3.7986E−03	7.2755E−03	5.3643E−03	4.9493E−03	1.4198E−03	8.14
	IAOA	1.9305E−02	3.8263E−02	3.0184E−02	3.3354E−02	8.4063E−03	8.17
	HDE	7.5246E−03	1.1804E−02	9.8422E−03	9.5700E−03	1.7323E−03	8.41
	OBGBO	3.9181E−03	6.3215E−03	4.7535E−03	4.3643E−03	1.0245E−03	76.47
60 °C	**OBEDO**	**3.7804E−03**	**3.7804E−03**	**3.7804E−03**	**3.7804E−03**	**2.1119E−09**	8.02
	EDO	8.6436E−03	4.4082E−02	2.2492E−02	1.9587E−02	1.3062E−02	**7.92**
	ADHHO	1.2592E−02	1.6298E−02	1.4632E−02	1.4330E−02	1.5458E−03	8.32
	OBMPA	6.0617E−03	9.3656E−03	7.3994E−03	7.4895E−03	1.3805E−03	8.30
	MGTO	3.7804E−03	4.6768E−03	3.9599E−03	3.7804E−03	4.0073E−04	8.40
	IAOA	1.1925E−02	6.9245E−02	4.0929E−02	4.9126E−02	2.5394E−02	8.27
	HDE	4.9433E−03	6.4997E−03	5.9688E−03	6.0547E−03	6.2993E−04	8.37
	OBGBO	3.7804E−03	3.8283E−03	3.7974E−03	3.7866E−03	2.1416E−05	76.31

Significant values are in [bold].

**Table 25:** Statistical analysis of Scenario 4 at different irradiances.

Irradiance	Algorithm	${\text{Min}}$	${\text{Max}}$	${\text{Mean}}$	${\text{Median}}$	${\text{STD}}$	${\text{RT}}$
1000 W/m^**2**^	**OBEDO**	**1.1462E−03**	**1.8801E−03**	**1.2990E−03**	**1.1465E−03**	**3.2506E−04**	8.32
	EDO	2.8393E−02	4.0383E−02	3.4843E−02	3.4925E−02	5.2988E−03	**7.87**
	ADHHO	2.6396E−02	3.6016E−02	3.1989E−02	3.4647E−02	4.7752E−03	8.32
	OBMPA	1.4319E−02	3.5555E−02	2.5853E−02	2.5350E−02	9.6933E−03	8.34
	MGTO	5.4408E−03	7.4378E−03	6.4914E−03	6.6208E−03	7.4834E−04	8.35
	IAOA	2.0753E−02	4.3751E−02	3.2781E−02	3.4879E−02	9.2212E−03	8.43
	HDE	1.2598E−02	1.6981E−02	1.5053E−02	1.5731E−02	1.7620E−03	8.54
	OBGBO	1.3549E−03	6.4825E−03	3.9939E−03	3.5462E−03	2.3104E−03	76.43
800 W/m^2^	**OBEDO**	**6.6858E−04**	**6.7014E−04**	**6.6890E−04**	**6.6860E−04**	**6.9355E−07**	8.19
	EDO	1.0357E−02	1.6213E−02	1.2227E−02	1.0605E−02	2.5825E−03	**7.67**
	ADHHO	1.0853E−02	1.5562E−02	1.2889E−02	1.2635E−02	1.9349E−03	8.28
	OBMPA	9.2702E−03	1.2831E−02	1.2119E−02	1.2831E−02	1.5924E−03	8.30
	MGTO	2.6706E−03	6.5733E−03	5.4675E−03	6.4783E−03	1.6925E−03	8.45
	IAOA	1.5824E−02	3.2148E−02	2.6286E−02	2.8032E−02	6.1850E−03	8.21
	HDE	6.8257E−03	7.9927E−03	7.4311E−03	7.6286E−03	5.5411E−04	8.58
	OBGBO	1.1001E−03	6.1838E−03	3.3585E−03	2.8579E−03	1.8971E−03	76.63
600 W/m^2^	**OBEDO**	**8.2395E−04**	**8.2901E−04**	**8.2527E−04**	**8.2395E−04**	**2.1953E−06**	8.12
	EDO	9.4736E−03	2.0551E−02	1.3030E−02	1.0812E−02	4.4525E−03	**7.72**
	ADHHO	4.9593E−03	9.9034E−03	8.4160E−03	8.9680E−03	1.9708E−03	8.32
	OBMPA	7.1003E−03	9.9642E−03	8.9004E−03	8.9159E−03	1.1778E−03	8.18
	MGTO	1.8937E−03	6.5632E−03	3.5364E−03	3.3995E−03	1.8240E−03	8.42
	IAOA	1.5062E−02	3.0464E−02	2.3971E−02	2.3236E−02	6.1079E−03	8.17
	HDE	5.6085E−03	9.9377E−03	7.6870E−03	7.2188E−03	2.1501E−03	8.49
	OBGBO	9.1622E−04	2.6642E−03	1.2932E−03	9.2806E−04	7.6779E−04	76.09
400 W/m^2^	**OBEDO**	**7.0761E−04**	**7.2169E−04**	**7.1144E−04**	**7.0772E−04**	**6.1134E−06**	8.16
	EDO	8.4576E−03	1.3343E−02	1.1252E−02	1.1374E−02	2.0143E−03	**7.98**
	ADHHO	3.7836E−03	6.7379E−03	4.8276E−03	4.5287E−03	1.1994E−03	8.17
	OBMPA	3.3034E−03	6.1513E−03	4.0450E−03	3.4791E−03	1.2095E−03	8.26
	MGTO	7.0860E−04	1.2310E−03	9.1996E−04	8.4686E−04	2.1193E−04	8.46
	IAOA	4.9593E−03	2.0606E−02	1.4812E−02	1.5614E−02	6.1086E−03	8.24
	HDE	2.3359E−03	3.2976E−03	3.0121E−03	3.0699E−03	3.9553E−04	8.19
	OBGBO	1.0206E−03	2.0961E−03	1.5361E−03	1.4958E−03	3.8367E−04	75.65
200 W/m^2^	**OBEDO**	**5.2054E−04**	**5.2108E−04**	**5.2065E−04**	**5.2054E−04**	**2.4090E−07**	7.98
	EDO	5.9319E−03	8.7368E−03	7.4880E−03	7.6239E−03	1.1204E−03	**7.43**
	ADHHO	1.1563E−03	4.2376E−03	2.4974E−03	1.8621E−03	1.4811E−03	8.22
	OBMPA	8.0751E−04	2.6493E−03	1.2709E−03	9.6697E−04	7.7821E−04	8.18
	MGTO	5.2054E−04	5.2272E−04	5.2126E−04	5.2055E−04	1.0148E−06	8.18
	IAOA	4.9016E−03	1.0825E−02	8.5011E−03	8.3110E−03	2.3066E−03	7.99
	HDE	6.7018E−04	7.8064E−04	7.4434E−04	7.5528E−04	4.3716E−05	8.13
	OBGBO	5.2055E−04	6.8215E−04	5.6703E−04	5.3965E−04	6.6348E−05	74.87

Significant values are in [bold].

The synergistic effect of the OBL strategy, alongside other optimization techniques embedded within the OBEDO, contributed to the algorithm's exceptional performance across different environmental contexts. In summary, the meticulous evaluation of the proposed OBEDO's performance in Scenario 5, through the assessment of statistical measures across distinct temperatures and irradiation conditions, underscored its effectiveness in achieving outstanding results. This success can be attributed to the successful integration of OBL, which bolstered both exploration and exploitation capabilities, ultimately identifying optimal PV module parameters across various conditions.

However, it is worth noting that the RT values acquired through utilizing the proposed algorithm across all operational conditions exhibit a marginal increase compared to those attained by the fundamental EDO. This observed difference in RT values can be attributed to amalgamating two distinct optimization strategies within the proposed algorithmic framework. Despite the slightly elevated RT values, the overall performance of the proposed algorithm surpasses that of the basic EDO by a notable margin, showcasing an improvement of over 75%. This significant enhancement can be attributed to the synergistic effect generated by the fusion of these diverse optimization strategies. The results obtained from the evaluation demonstrate a remarkable alignment between the I–V and P–V characteristics deduced from the estimated parameters and the experimental data, even under varying temperature and irradiance conditions. This alignment underscores the precision of the proposed algorithm in capturing the intricate nuances of the photovoltaic system's behaviour. Furthermore, the outcomes of the experimentation reveal an additional advantage of the OBEDO, namely its ability to attain a lower RMSE value. This improvement in accuracy highlights the algorithm's efficacy in modelling and predicting the behaviour of the photovoltaic module, thereby facilitating more reliable parameter estimations. The comprehensive discussions and analyses led to the clear conclusion that the efficiency of the OBEDO remains robust and dependable when confronted with dynamic shifts in environmental conditions. The amalgamation of optimization strategies, while contributing to a slight increase in RT values, offers substantial gains in performance, as evident from the substantial enhancement over the basic EDO. The alignment between simulated I–V and P–V characteristics and experimental data, coupled with the lower RMSE value achieved, attests to the algorithm's capability to handle the details of dynamic environmental variations.

### Statistical performance

Milton Friedman, a renowned figure in statistics, is credited with conceiving a significant non-parametric statistical test known as the Friedman ranking test (FRT). This test holds a distinct purpose in the realm of statistical analysis—it serves as a valuable tool for detecting variations in treatments across multiple experimental runs, akin to the parameterized repeated measures ANOVA commonly employed in research. A comprehensive application of the FRT was conducted to establish the superiority of the OBEDO in terms of overall performance. In this endeavour, the objective was to substantiate that the OBEDO exhibits superior efficacy in generating aggregate results compared to a range of other algorithms. Among the algorithms subjected to analysis were not only the OBEDO and EDO but also an assortment of others, namely the ADHHO, OBMPA, MGTO, IAOA, HDE, and OBGBO. The process involved meticulously examining the FRT outcomes, which were carefully obtained by considering each algorithm's RMSE standard deviation values. These values, extracted and compiled in Table 26, provided a foundation for an extensive comparative analysis of the various algorithms under investigation. The resultant observations from this comparison laid bare a noteworthy revelation – the OBEDO emerged as a standout performer. By assessing the mean FRT values across four distinct case studies and comparing them against the benchmark set by preceding algorithms, the authors concluded that the recommended OBEDO showcases an exceptional ability to manage diverse scenarios with the lowest mean FRT values. In light of the compelling evidence amassed through this comprehensive analysis, it becomes evident that the OBEDO outshines its counterparts. The OBEDO's consistent superiority across multiple case studies firmly establishes its prowess as a premier algorithmic solution, further reinforcing its position as a robust and proficient tool for addressing various optimization challenges.

**Table 26:** Statistical performance comparison.

Scenario	Models	FRT Values							
		OBEDO	EDO	ADHHO	OBMPA	MGTO	IAOA	HDE	OBGBO
1	SDM	1.10	7.00	6.10	5.40	1.90	7.30	4.20	3.00
	DDM	1.90	7.70	5.70	5.50	2.10	6.80	3.90	2.40
	TDM	1.60	6.40	5.90	4.80	2.50	7.40	4.70	2.70
2	SDM	1.80	6.40	4.10	5.75	2.70	7.80	4.35	3.10
	DDM	2.20	6.10	3.40	6.00	4.95	5.90	5.30	2.15
	TDM	2.50	7.30	5.60	5.40	2.00	5.60	4.70	2.90
3	SDM	1.00	7.50	6.70	5.00	2.90	6.50	3.20	3.20
	DDM	1.60	7.30	6.20	4.50	4.30	6.10	3.50	2.50
	TDM	1.20	7.50	6.30	3.80	4.80	5.60	3.70	3.10
4	SDM	1.20	6.20	6.40	5.60	2.60	7.00	4.60	2.40
5	25 °C	1.00	6.40	6.80	5.80	3.00	6.60	4.40	2.00
	40 °C	1.00	7.40	6.40	4.60	2.60	7.20	4.40	2.40
	60 °C	1.40	7.00	6.40	4.80	1.80	7.60	4.20	2.80
	1000W/m^2^	1.00	7.00	6.60	5.80	3.00	6.60	4.00	2.00
	800W/m^2^	1.00	6.20	6.20	5.80	2.80	7.80	4.00	2.20
	600W/m^2^	1.00	6.60	4.80	5.60	3.00	8.00	4.80	2.20
	400W/m^2^	1.00	7.20	6.00	5.20	2.00	7.60	4.00	3.00
	200W/m^2^	1.40	7.40	5.80	5.20	1.60	7.60	4.00	3.00
Mean FRT		**1.38**	6.92	5.86	5.25	2.81	6.94	4.22	2.61
Rank		**1**	7	6	5	3	8	4	2

Significant values are in [bold].

Table 26 presents a comparative analysis using the FRT across various scenarios and optimization models. The FRT aims to determine the relative performance of different optimization algorithms. The models evaluated in this study include OBEDO, EDO, ADHHO, OBMPA, MGTO, IAOA, HDE, and OBGBO. In Scenario 1, involving different data models (SDM, DDM, TDM), the FRT values indicate the algorithms' performance. The OBEDO algorithm achieved the lowest FRT value, followed by MGTO and OBGBO. Similar trends are observed in Scenario 2, which considers another set of data models, with OBEDO outperforming other models. Scenario 3 explores algorithms' performance across diverse conditions. Once again, OBEDO takes the lead in achieving the lowest FRT value, followed by ADHHO and IAOA. Scenario 4 explores algorithms' performance across diverse conditions. Once again, OBEDO takes the lead in achieving the lowest FRT value, followed by OBGBO and MGTO. In the fifth scenario, different temperature and irradiance conditions are analyzed. OBEDO consistently demonstrates the most favourable performance across varying environmental conditions. The mean FRT provides the average FRT values for each model, and the rank presents their ranking based on performance. Notably, OBEDO maintains the top rank with the lowest mean FRT value, emphasizing its consistent superiority. In summary, the FRT analysis across multiple scenarios and models underscores the exceptional performance of the OBEDO algorithm, which consistently achieves the lowest FRT values and the highest ranking. This suggests that OBEDO outperforms the other optimization models across diverse conditions and demonstrates its robustness and efficacy in optimization tasks.

## Conclusions and future extension

The proposed OBEDO has demonstrated its prowess in efficiently, accurately, and rapidly extracting undefined parameters from diverse PV system models, as evidenced by the proposed study. The comprehensive analysis undertaken further substantiates the efficacy and potential of this framework in extrapolating parameters for commercial PV models. The evaluation sought to ascertain if the proposed methodology could successfully deduce the parameters of such intricate models, and the obtained results provide compelling evidence of the OBEDO's capability to precisely and effectively determine optimal parameters. Furthermore, it is noteworthy that the TDM exhibits the lowest model features in terms of RMSE, IAE, and RE, underscoring its accuracy. Additionally, the convergence speed of the OBEDO outperforms its counterparts across all test cases, indicating its superior efficiency. These attributes collectively position the proposed OBEDO as a cutting-edge, competitive, and advanced approach for parameter identification in PV models. The OBEDO attains stability and diversity, achieved through meticulous parameter tuning, which positions it favourably compared to its basic version. Nonetheless, as per the NFL theorem, it is crucial to recognize that while the OBEDO exhibits enhanced search results for parameter identification problems, its success might vary in problems with distinct characteristics. As a result, ongoing research and development are necessary to tailor meta-heuristic search approaches to specific problem domains.

In forthcoming research, the proposed algorithm can be evaluated within contemporary techniques for PV parameter identification and subsequently extended to real-world scenarios for various PV cells/modules. Moreover, the versatile applicability of the OBEDO can be harnessed to address challenging problems spanning water pollution prediction, disease diagnosis, binary optimization, feature selection/extraction, image processing tasks, scheduling optimization, wireless sensor networks, and medical image categorization. This underscores the algorithm's potential as a multifaceted solution with far-reaching implications across diverse domains.

## Data Availability

The datasets used during the current study are available from the corresponding author upon reasonable request.
